# Laminin 511-E8, an autoantigen in IgG4-related cholangitis, contributes to cholangiocyte protection

**DOI:** 10.1016/j.jhepr.2024.101015

**Published:** 2024-01-23

**Authors:** David C. Trampert, Remco Kersten, Dagmar Tolenaars, Aldo Jongejan, Stan F.J. van de Graaf, Ulrich Beuers

**Affiliations:** 1 Department of Gastroenterology and Hepatology, Tytgat Institute for Liver and Intestinal Research, Amsterdam Gastroenterology Endocrinology Metabolism (AGEM), Amsterdam UMC, Location AMC, University of Amsterdam, Amsterdam, The Netherlands; 2 Department of Epidemiology & Data Science, Bioinformatics Laboratory, Amsterdam Public Health Research Institute, Amsterdam UMC, Location AMC, University of Amsterdam, Amsterdam, The Netherlands

**Keywords:** Activated T lymphocytes, Biliary bicarbonate umbrella, Cholangiopathy, Epithelial barrier function, GCDC, Glycochenodeoxycholate, Hydrophobic bile acids, IgG4-related disease, IgG4-RD, Immune-mediated disease, Laminin

## Abstract

**Background & Aims:**

IgG4-related cholangitis (IRC) is the hepatobiliary manifestation of IgG4-related disease. Anti-laminin 511-E8 autoantibodies have been identified in its pancreatic manifestation. Laminin 511-E8 promotes endothelial barrier function, lymphocyte recruitment, and cholangiocyte differentiation. Here, we investigate anti-laminin 511-E8 autoantibody presence in IRC, and mechanisms via which laminin 511 may contribute to cholangiocyte protection.

**Methods:**

Anti-laminin 511-E8 serum autoantibody positivity was assessed by ELISA. RNA sequencing and RT-qPCR were performed on human H69 cholangiocytes treated with recombinant laminin 511-E8. H69 cholangiocytes were subjected to shRNA knockdown targeting genes encoding laminin 511 (*LAMA5*, *LAMB1*, *LAMC1*) or treated with recombinant laminin 511-E8. Cholangiocellular bile acid influx was quantified radiochemically using 22,23-^3^H-glycochenodeoxycholic acid (GCDC). GCDC-induced apoptosis was determined by Caspase-3/7 assays. Cholangiocellular barrier function was assessed by FITC-Dextran permeability assays. Immunofluorescent staining of laminin 511 and claudin 1 was performed on extrahepatic bile duct tissue of control and anti-laminin 511-E8 positive individuals with IRC.

**Results:**

Seven out of 52 individuals with IRC had autoantibodies against laminin 511-E8. Recombinant laminin 511-E8 led to differential expression of genes involved in secretion, barrier function, and inflammation. Knockdown of laminin 511 constituents increased toxic bile acid permeation and GCDC-induced apoptosis. Laminin 511-E8 treatment decreased toxic bile acid permeation and dose-dependently alleviated GCDC-induced apoptosis. *LAMA5* and *LAMC1* knockdown increased transepithelial permeability. Laminin 511-E8 treatment reduced transepithelial permeability and prevented T lymphocyte-induced barrier dysfunction. Laminin 511 and claudin 1 staining patterns appeared altered in anti-laminin 511-E8 positive individuals with IRC.

**Conclusions:**

Laminin 511-E8 is an autoantigen in subsets of individuals with IRC. Laminin 511 enhances cholangiocellular barrier function and protects cholangiocytes against T lymphocyte-induced barrier dysfunction, toxic bile acid permeation and bile acid-induced apoptosis.

**Impact and implications:**

A subset of patients with IgG4-related cholangitis (IRC) has autoantibodies against laminin 511-E8. In human cholangiocytes, laminin 511 protects against (T lymphocyte-induced) epithelial barrier dysfunction and hydrophobic bile acids. Laminin 511 and claudin 1 staining may be altered in extrahepatic bile ducts of patients with IRC who are anti-laminin 511-E8 positive. This makes it tempting to speculate that a decreased epithelial barrier function with attraction of immune cells and impaired bicarbonate secretion as a result of dysfunction of laminin 511 by autoantibody binding could potentially be a common systemic pathogenic mechanism in a subset of patients with IgG4-RD.

## Highlights

• Laminin 511-E8 is an autoantigen in a subset of individuals with IRC.
• Laminin 511 protects cholangiocytes against toxic bile acids.
• Laminin 511 improves cholangiocellular barrier function.
• Laminin 511 protects the cholangiocyte barrier from T lymphocyte-induced damage.
• Laminin 511 and claudin 1 expression may be altered in individuals with IRC.

## Graphical abstract

**Figure:**
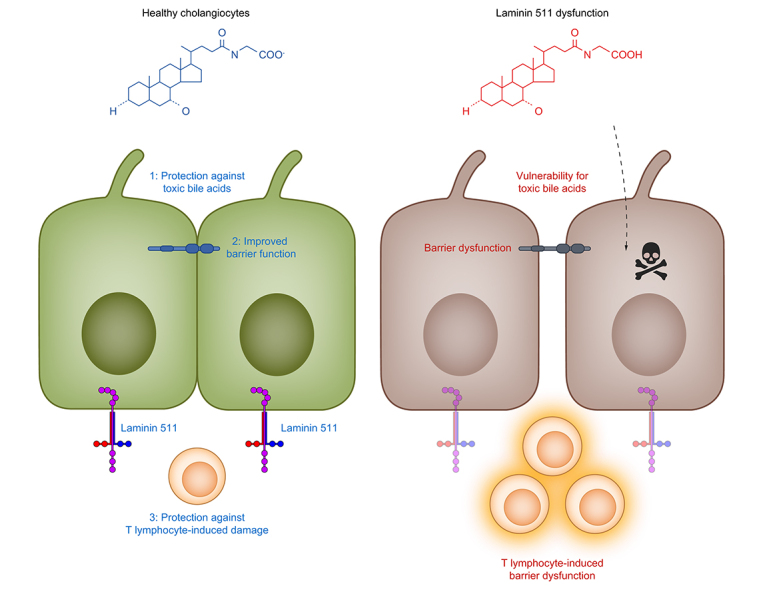

## Introduction

IgG4-related disease (IgG4-RD) is a systemic, lymphocyte driven, fibroinflammatory disorder characterized by elevated IgG4 serum levels and typical histopathological findings in affected organs. The most common abdominal organ manifestations in IgG4-RD are autoimmune pancreatitis (type I AIP) and IgG4-related cholangitis (IRC).^1^^,^^2^ The fibroinflammatory nature of IRC leads to thickening of the bile duct wall and stenosis formation, both of which are features overlapping with hallmark signs of primary sclerosing cholangitis (PSC) and cholangiocarcinoma (CCA). Currently, no single accurate diagnostic test is available to distinguish IRC from these conditions. IRC therefore presents a major diagnostic challenge, with misdiagnosis and unjustified major surgical resections being a relatively common occurrence (Table S1).^3^

In light of this clinical problem, understanding the pathogenesis of IRC is of great importance.

Previous studies have demonstrated the presence of affinity-maturated dominant B cell receptor clones in peripheral blood and affected tissue of individuals with IgG4-RD.[4], [5], [6] This raised the suspicion that specific autoantibodies are present in patients with IgG4-RD and led to the discovery of the autoantigens annexin A11 (ANXA11), galectin-3, laminin 511-E8, and prohibitin 1.[7], [8], [9] The pathogenicity of autoantibodies was strongly suggested after injection of patient-derived IgG1 or IgG4 autoantibodies into mice led to the development of typical organ lesions in salivary glands and pancreas which were more intense after IgG1 than IgG4 injection.^10^ Simultaneous injection showed that IgG4 alleviated the more pathogenic effects of IgG1. In the same line, IgG4 autoantibodies directed against annexin A11 blocked binding of IgG1 autoantibodies to annexin A11 supporting an anti-inflammatory role of IgG4 in IgG4-RD.^7^ Additional support for the pathogenicity of autoantibodies in IgG4-RD comes from the observation that individuals with IgG4-RD who are positive for multiple autoantibodies have increased disease severity^11^ and that autoantibody levels decrease after successful treatment.^8^ Autoantibodies could potentially contribute to the pathogenesis of IgG4-RD either by directly interfering with the endogenous function of the targeted autoantigen or by eliciting an excessive immune response after binding to the autoantigen. To address the potential direct pathogenicity of these autoantibodies, an improved understanding of the physiological role of the targeted autoantigens is pivotal.

To this end, we have previously investigated the role of the autoantigen annexin A11 in cholangiocytes,^12^ which has a critical role in trafficking anoctamin 1 (ANO1), a calcium-activated chloride channel, to the apical cholangiocyte membrane. Patient serum containing autoantibodies directed against annexin A11 inhibited the membrane trafficking of ANO1, thereby potentially preventing its function at the plasma membrane of establishing a chloride gradient to facilitate biliary bicarbonate secretion. Biliary bicarbonate secretion is thought to keep millimolar levels of bile salts within the bile duct lumen in their deprotonated, membrane-impermeable and thus non-toxic state.^13^^,^^14^ These findings are of interest as the autoantigen laminin 511-E8 has been shown to promote the differentiation of induced pluripotent stem cells (iPSCs) towards cholangiocytes with upregulation of secretory components such as the cystic fibrosis transmembrane conductance regulator (*CFTR*), the G protein-coupled bile acid receptor 1 (*GPBAR1*, *TGR5*), and secretin receptor (*SCTR*), which are all instrumental in adequate apical bicarbonate secretion.^15^ Laminin 511 is a heterotrimeric extracellular matrix protein composed of the subunits alpha-5, beta-1, and gamma-1.^16^ Anti-laminin 511 autoantibodies are directed against the cell-binding proteolytic laminin 511-E8 fragment in type 1 AIP.^8^

In addition to potential secretory defects in IRC, an impaired cholangiocellular barrier function has been proposed to play a role in IRC. Bile and brush cytology samples taken upon endoscopic retrograde cholangiopancreaticography (ERCP) from individuals with IRC demonstrated an increase in the T cell cytokines IL-4 and IL-13.^17^ These cytokines negatively impacted cholangiocellular barrier function *in vitro* through differential expression of tight junction-associated claudin genes *CLDN1* and *CLDN2*. Numerous T cell populations are involved in the pathogenesis of IgG4-RD including regulatory T cells, follicular T helper 2, T peripheral helper, and cytotoxic CD8^+^ and CD4^+^ T cells.^2^^,^^18^ Notably in endothelial cells, laminin 511 has been shown to strengthen endothelial barrier function by stabilizing vascular endothelial-cadherin and increasing the expression of tight junction proteins.^19^ At the endothelial barrier, laminin 511 prevents the extravasation of leukocytes^20^^,^^21^ and activation of T cells.^22^

In the present study, we investigate whether laminin 511-E8 is an autoantigen in IgG4-related cholangitis and study the role of laminin 511 in human cholangiocytes. Based on our own studies and the current literature, we hypothesize that laminin 511 protects cholangiocytes against toxic bile acids and prevents T lymphocyte-induced barrier dysfunction.

## Patients and methods

### Human ethics statement

The use of serum samples and peripheral blood mononuclear cells (PBMCs) was approved by the local medical ethical committee in Amsterdam (MEC 10/007 and MEC 2020/081). All participants gave written informed consent before inclusion in the study. Resection specimens of extrahepatic bile duct tissue from control and anti-laminin 511-E8 positive individuals with IRC were obtained from the Amsterdam UMC HPB Pathology biobank (TcB2018-063).

### Participants

Serum samples were obtained from individuals with IRC, PSC, CCA, and healthy volunteers. Diagnosis of IRC was made according to the HISORt criteria (histology, imaging, serology, other organ involvement, response to therapy),^23^ whereas diagnosis of PSC was made according to the EASL Clinical Practice Guidelines on sclerosing cholangitis.^24^ Diagnosis of CCA was made based on histopathology.

### ELISA

Alternating rows of 96-well ELISA plates were coated with 100 μl human recombinant laminin 511-E8 (2 μg/ml) or bovine serum albumin (2 μg/ml) in coating buffer (50 mM carbonate/bicarbonate pH 9.6 and incubated at 37 °C for 1 h.^11^ Wells were washed with 50 mM Tris-buffered saline (TBS) with 0.05% Tween (TBST) pH 8.0 and subsequently blocked for 1 h with 1% bovine serum albumin (BSA)/TBS pH 8.0 at room temperature. Wells were again washed and incubated with diluted sera from patients with IRC, PSC, CCA, or healthy controls (1:20 in 1% BSA/TBS pH 8.0) for 30 min at room temperature. After washing, wells were incubated for 1 h at room temperature with 0.65 μg/ml of rabbit anti-human IgG horseradish peroxidase (HRP) secondary antibody. After washing, bound reactants were detected after a 5-min incubation with 100 μl of the chromogenic substrate 3,3′,5,5′-tetramethylbenzidine. The reaction was stopped with 100 μl of stop solution and absorbance was determined at 450 nm using the CLARIOstar (BMG LABTECH, Ortenberg, Germany). Samples were considered positive for autoantibodies against laminin 511-E8 when absorbance values were greater than the mean plus two standard deviations of the healthy control group as previously described.^9^^,^^11^

### Cell cultures

H69 cholangiocytes were kindly provided by Dr Douglas Jefferson (Tufts University, Boston, MS, USA) and were cultured as previously described.^25^ Composition of H69 culture medium can be found in Table S2. Culture details on LX2 cells can be found in the Supplementary methods. *Mycoplasma* contamination tests were carried out every 3 months with negative results throughout.

### RNA sequencing

H69 cholangiocytes were treated with recombinant human laminin 511-E8 0.25 μg/cm^2^ for 48 h. After RNA isolation by spin column, concentration, and quality were assessed using the RNA ScreenTape. Library preparation by poly(A) mRNA capture was performed using KAPA mRNA Hyperprep. Samples were pair-end sequenced (150 bp) on the NovaSeq (Illumina, San Diego, CA, USA) with a sequencing depth of 40 million reads per sample. Raw data were submitted to NCBI Gene Expression Omnibus (GEO) and are freely available under GEO accession number GSE221746. For the RNA sequencing data analysis pipeline used, see the Supplementary methods.

### Human PBMCs isolation and activation of T lymphocytes

Isolation of PBMCs, depletion of monocytes/macrophages by the adherence method, and activation of T lymphocytes was performed as previously described.[26], [27], [28] Peripheral blood was obtained from healthy volunteers and transferred to 50-ml conical tubes in an ML-2/BSL-2 facility. Ficoll-Paque Plus was added to the bottom of the 50-ml conical tube and centrifuged to generate a gradient. The PBMC layer was collected in a new tube, diluted with sterile filtered 0.2% BSA 2 mM EDTA in PBS and centrifuged. Supernatant was discarded and PBMCs were resuspended in Iscove’s modified Dulbecco’s medium (IMDM) supplemented with 10% fetal bovine serum and 37.5 U/ml (1%) penicillin, 37.5 μg/ml (1%) streptomycin. PBMCs were seeded in culture plates for 2 h to deplete monocytes/macrophages by adherence and T lymphocytes were stimulated using phorbol-12-myristate-13-acetate (PMA) 50 ng/ml and ionomycin 1000 ng/ml.^28^ Non-adherent activated T lymphocytes were collected and centrifuged. Pelleted cells were resuspended in supplemented IMDM and counted for co-culture plating.

### Fluorescein isothiocyanate–Dextran permeability assay in a Transwell co-culture system

On Day 0, 12-well Transwell inserts were coated with 10 μg/ml human recombinant laminin 511-E8 in sterile PBS or 10 μg/ml BSA control coating, and left to dry for 1 h at 37 °C. After coating, H69 cholangiocytes were seeded in the apical compartment at 100,000 cells per insert. On Day 4, PBMCs were isolated from healthy volunteers, and T lymphocytes were activated with PMA and ionomycin, after which 200,000 cells were added to the basolateral compartment. The apical compartment was refreshed with H69 culture medium and the basolateral compartment contained supplemented IMDM with or without activated T lymphocytes. Co-culture was left overnight. On Day 5, 4 kDa fluorescein isothiocyanate (FITC)-Dextran permeability assays were performed. For this, medium was refreshed with 800 μl DMEM supplemented with 10% FBS and 37.5 U/ml (1%) penicillin, 37.5 μg/ml (1%) streptomycin on the basolateral side, and 250 μl of supplemented DMEM containing 1 mg/ml 4 kDa FITC–Dextran on the apical side. At timepoint 0, 100 μl of medium was transferred to a black 96-well plate to determine potential background fluorescence. In addition, an empty unseeded Transwell insert was included to determine maximal permeability of the insert itself. At t = 60, 120, 180, and 240 min, 100 μl of basolateral medium was collected per experimental condition and transferred to the black 96-well plate. FITC–Dextran fluorescence (excitation 490 nm, emission 520 nm) was measured using the CLARIOstar apparatus.

### Lentivirus generation and transduction for shRNA-mediated knockdown

Lentiviral short hairpin RNA (shRNA) constructs were purchased from the MISSION® TRC version 1 shRNA library (Sigma-Aldrich, St. Louis, MO, USA): *LAMA5* (TCRN0000119152), *LAMB1* (TRCN0000083431), *LAMC1* (TRCN0000119110) and the non-targeting shRNA control (SHC002). Lentivirus was produced as previously described in an ML-2/BSL-2 facility.^29^ Transduced H69 cholangiocytes were selected with 1 μg/ml puromycin to obtain stable *LAMA5*, *LAMB1,* and *LAMC1* knockdown cholangiocytes.

### Western blotting

Cells were lysed in radioimmunoprecipitation assay buffer and lysate samples were prepared in lithium dodecyl sulfate sample buffer. After running the samples on SDS-PAGE gels (3–15% Tris-acetate for LAMA5, 4–10% Tris-glycine for LAMB1 and LAMC1) in running buffer (50 mM tricine, 50 mM Tris, 0.1% SDS, and 1.3 mM sodium bisulfite at pH 8.2), samples were transferred by wet-transfer in ethanolamine/glycine transfer buffer to polyvinylidene difluoride membranes, blocked for 2 h at room temperature in 5% non-fat milk/TBST and probed overnight at 4 °C with the respective primary antibody (see CTAT methods). Immune complexes were detected with HRP-conjugated secondary antibodies and visualized using enhanced chemiluminescence detection reagent (Lumi-light, Roche Diagnostics, Rotkreuz, Switzerland) and ImageQuant LAS 4000 (GE Healthcare, Chicago, IL, USA). Protein bands were quantified using ImageJ 1.50i (Wayne Rasband, National Institutes of Health, Bethesda, MD, USA). Results were presented as fold changes of control (shRNA control; SHC002) transduced H69 cells, normalized per experiment.

### RNA isolation, cDNA synthesis, and real-time quantitative PCR

For RNA isolation, cDNA synthesis, primer design, RT-qPCR and data analysis, see Supplementary methods.

### Bile acid permeation assay

Bile acid permeation assays were performed as previously described.^12^

### Caspase-3/7 assay

Apoptosis was determined with Caspase-3/7 assays in H69 cholangiocytes exposed to 1,500 μM glycochenodeoxycholic acid (GCDC) or 10 μM Raptinal as a positive control as described previously.^13^^,^^30^

### Immunofluorescent staining and confocal microscopy

Paraffin-embedded extrahepatic bile duct tissue of control and anti-laminin 511-E8 positive individuals with IRC were sectioned at 4 μm thickness. Slides were deparaffinized, re-hydrated and unmasked in a steamed Trilogy (Cell Marque) solution. Additional antigen retrieval was done in steamed 10 mM sodium citrate (Sigma) solution. Autofluorescence was quenched using 50 mM NH_4_Cl in PBS pH 7.4 at room temperature. Slides were blocked in 5% normal goat serum in 1% BSA/TBST for 1 h. Primary antibodies were added to the tissue sections at a 1:100 dilution in 1% BSA in TBST overnight at 4 °C. Alexa Fluor 488 was used as secondary antibody for laminin 511-E8 and claudin 1, whereas Alexa Fluor 568 and 594 were used for cytokeratin 7 alongside Hoechst 33324 for nuclear staining. See CTAT methods for all antibodies used. Tissue sections were mounted with Prolong Gold antifade (Invitrogen) and 1.5 mm cover glasses. Stainings were imaged using the SP8-X DLS LightSheet confocal microscope by a researcher blinded to patient conditions. Three randomly selected fields were captured for each condition.

### Statistical analysis

Data are presented as means with standard deviations. Statistical analyses were performed with GraphPad Prism 9 (GraphPad, La Jolla, CA, USA). Results of two groups were compared with a paired or unpaired *t* test where appropriate. One-way ANOVA was used when comparing multiple groups. Values of *p* <0.05 were considered statistically significant.

## Results

### Autoantibodies against laminin 511-E8 are present in a subset of individuals with IRC

**Fig. 1:**
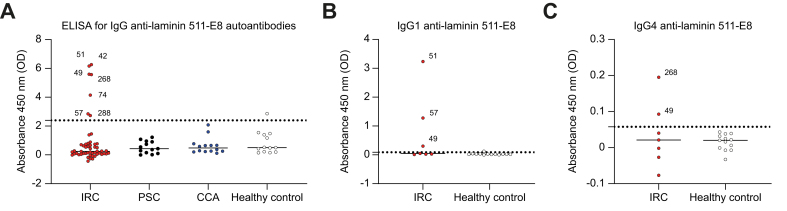
Laminin 511-E8 is an autoantigen in a subset of patients with IRC. (A) Anti-laminin 511-E8 IgG in patients with IRC (7/52), PSC (0/13), CCA (0/14) and healthy controls (1/13). Anti-laminin 511-E8 IgG1 (B) and IgG4 (C) subtype classification in positive patients with IRC. Patients are presented as individual data points and positive patients are annotated. CCA, cholangiocarcinoma; IRC, IgG4-related cholangitis; PSC, primary sclerosing cholangitis.

Seven out of 52 individuals with IRC (13.5%) were found to have autoantibodies against laminin 511-E8, but none of the disease controls with PSC (n = 13) or CCA (n = 14). One of 13 healthy volunteers had autoantibodies against laminin 511-E8 (7.7%) (Fig. 1A). IgG subtype classification showed that two individuals with IRC had autoantibodies of the IgG1 subtype, one had autoantibodies of the IgG4 subtype and one had autoantibodies of both the IgG1 and IgG4 subtype (Fig. 1B and C). There were no differences between individuals with IRC positive or negative for laminin 511-E8 autoantibodies regarding age, sex, multi-organ involvement, the occurrence of malignancies, major hepatobiliary surgeries or serum liver and inflammatory tests at presentation (Table 1). Six out of seven patients had a history of ‘blue-collar work’, that is, skilled trades frequently involving manual labor, and had been exposed to occupational toxins and chemicals (*e.g.* gases, fumes, mineral dusts) for >1 year (Table 2).^31^^,^^32^ Four laminin 511-E8 positive individuals with IgG4-RD had manifestations in other secretory organs, namely the pancreas, salivary glands, kidneys, lungs, and colon. Autoantibodies targeting other autoantigens were found in six IRC-affected individuals and four underwent major hepatobiliary surgeries for the suspicion of CCA (Table S1). Malignancy occurred in four individuals either before or after the diagnosis of IRC. In four individuals there was a considerable delay in the diagnosis of IRC, ranging from 9 to 360 months. Owing to the diagnostic challenge of IRC and accompanying initial misdiagnosis, patients were treated in different ways, with varying rates of clinical and biochemical success (Fig. S1). Thus, in our IRC cohort no relevant occupational, clinical, or biochemical differences were observed between laminin 511-E8-positive and -negative individuals.

**Table 1:** Comparison of IRC patients positive and negative for anti-laminin 511-E8 autoantibodies.

	Positive	Negative	*p* value
IRC patients, n (%)	7 (13.5)	45 (86.5)	
Sex (male/female)	5/2	41/4	0.1859
Age (years)	60 (42–65)	63 (51–69)	0.3344
Multi-organ, n (%)	4 (57)	33 (82.5)	0.3763
Malignancy, n (%)	4 (57)	15 (34)	0.4016
Major HPB surgery, n (%)	4 (57)	13 (29.5)	0.2033
Bilirubin (μmol/L)	92 (29–123)	56 (33–143)	0.7346
ALP (IU/L)	445 (322–560)	354 (242–638)	0.6964
gGT (IU/L)	362 (192–887)	508 (265–816)	0.6207
AST (IU/L)	93 (51–167)	83 (69–195)	0.6058
ALT (IU/L)	148 (97–234)	117 (79–330)	0.5542
CA19.9 (kU/L)	191 (183–220)	73 (24–239)	0.6465
CRP (mg/L)	30 (23–83)	11 (5–31)	0.9766
ESR (mm/h)	69 (47–88)	44 (20–71)	0.2135

Levels of significance: *p* < 0.05 was seen as significant. For categorical data (sex, multi-organ, malignancy, major HPB surgery) Fisher’s exact test was performed. For numerical data (age, bilirubin, ALP, gGT, AST, ALT, CA19.9, CRP, ESR) an unpaired *t* test was performed.

ALP, alkaline phosphatase; ALT, alanine aminotransferase; AST, aspartate aminotransferase; CA19.9, cancer antigen 19.9; CRP, c-reactive protein; ESR, erythrocyte sedimentation rate; gGT, gamma-glutamyl transferase; HPB, hepatopancreatobiliary; IRC, IgG4-related cholangitis.

**Table 2:** Characteristics of individuals with IRC positive for anti-laminin 511-E8 autoantibodies.

	#49	#42	#51	#74	#268	#288	#57
Age at onset (years)	60	52	63	42	74	65	34
Time to diagnosis (months)	3	4	9	2	93	10	360
Sex	M	M	M	F	M	M	F
Profession	Driver petrochemical company	Truck driver	Military service, limousine driver	Beautician, car sales	—	Construction worker	Cleaner
Organ involvement	IRC AIP Salivary glands	IRC AIP Kidney	IRC	IRC	IRC	IRC AIP	IRC AIP Lung, colon
Autoantibody positivity	Laminin 511-E8	Laminin 511-E8 Annexin A11 Prohibitin 1	Laminin 511-E8 Prohibitin 1	Laminin 511-E8 Prohibitin 1	Laminin 511-E8 Galectin-3 Prohibitin 1	Laminin 511-E8 Prohibitin 1	Laminin 511-E8 Prohibitin 1 Annexin A11
Surgery	No	No	Hemihepatectomy	PPPD Hemihepatectomy	Bile duct resection	Bile duct resection	No
Malignancy	No	No	CRC 2 years after IgG4-RD onset	CCA at diagnosis, 2× recurrence after IRC diagnosis	No	Sigmoid carcinoma 6 years prior to IgG4-RD onset	CCA 27 years after first manifestation
Therapy	ERCP with stent placement Prednisolone	Spontaneous improvement	UDCA	Prednisolone Azathioprine/6-Thioguanine UDCA	No therapy	ERCP with stent placement Prednisolone 6-Thioguanine UDCA	Stent placement UDCA Prednisolone

AIP, type 1 autoimmune pancreatitis; CCA, cholangiocarcinoma; CRC, colorectal carcinoma; ERCP, endoscopic retrograde cholangiopancreaticography; IRC, IgG4-related cholangitis; PPPD, pylorus-preserving pancreatoduodenectomy; PSC, primary sclerosing cholangitis; UDCA, ursodeoxycholic acid.

### Laminin 511 constituents are highly expressed in models of human cholangiocytes

**Fig. 2:**
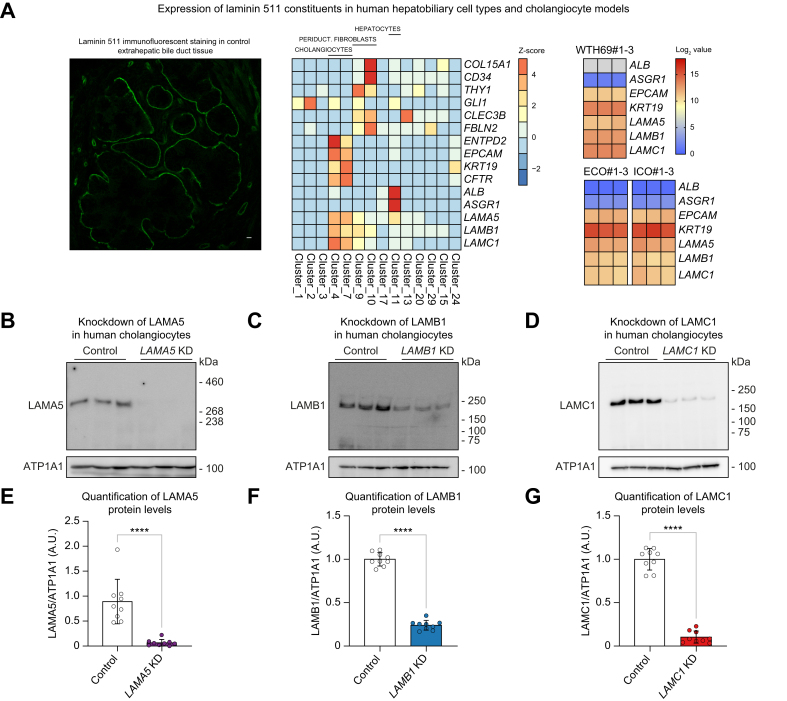
Laminin 511 constituents are expressed in various human cholangiocyte models. (A) Laminin 511 immunofluorescent staining in human hepatobiliary control tissue discloses a sharp peribiliary staining pattern (scale bar = 10 μm). Left heatmap represents laminin 511 gene constituent expression in cholangiocyte, hepatocyte, and periductal fibroblast clusters from human liver. Right heatmaps demonstrate expression levels of laminin 511 constituents in two different human cholangioid models and H69 cholangiocytes. Establishment of (B) LAMA5 (400 kDa), (C) LAMB1 (198 kDa), and (D) LAMC1 (178 kDa) shRNA knockdown H69 cholangiocytes. Quantification of (E) LAMA5, (F) LAMB1, and (G) LAMC1 protein levels normalized by ATP1A1 (112 kDa), (nine cell samples from n = 3 independent experiments). Data are represented as mean with standard deviation. Levels of significance: (E–G) ∗∗∗∗*p* <0.0001; unpaired *t* test. A.U., arbitrary units; Control, shRNA control; ECO/ICO, extra-/intrahepatic cholangiocyte organoids; KD, knockdown; *LAMA5*, laminin alpha 5 chain; *LAMB1*, laminin beta 1 chain; *LAMC1*, laminin gamma 1 chain.

As cholangiocytes are the main cell type affected in IRC, laminin 511 expression was assessed in human control extrahepatic bile duct tissue by immunofluorescence (Fig. 2A). A sharp peribiliary staining pattern was observed. To differentiate the cellular origin of peribiliary laminin staining in cholangiocytes *vs.* periductal fibroblasts, gene constituent expression of laminin was studied in human cholangiocytes and compared with periductal fibroblasts that also could contribute to laminin 511 production. The laminin 511 gene constituent expression appeared highest in the cholangiocyte population (Fig. 2A). Additionally, on a protein level, LAMA5 expression was negligibly low in activated myofibroblasts compared with cholangiocytes (Fig. S2). To further determine the cholangiocellular expression of *LAMA5*, *LAMB1,* and *LAMC1*, two types of human cholangiocyte organoids and human H69 cholangiocytes were assessed. In all datasets, a cholangiocyte phenotype was demonstrated by high expression of cholangiocellular markers *EPCAM* (epithelial cell adhesion molecule) and *KRT19* (cytokeratin 19), whereas expression of the hepatocyte markers *ALB* (albumin) and *ASGR1* (asialoglycoprotein receptor 1) was low. The three genetic laminin 511 constituents *LAMA5, LAMB1, LAMC1* showed high expression across all types of cholangiocyte organoids and human H69 cholangiocytes (Fig. 2A). Thus, laminin is highly expressed in human cholangiocytes, but not periductal fibroblasts.

To further investigate the physiological role of laminin 511 in cholangiocytes, stable knockdown cell lines for *LAMA5*, *LAMB1,* and *LAMC1* were generated in human H69 cholangiocytes by lentiviral shRNA transduction. Expression of laminin 511 constituents and their respective knockdown were demonstrated on the protein level by Western blot; *LAMA5* (83% knockdown), *LAMB1* (76% knockdown), and *LAMC1* (90% knockdown) (Fig. 2B–G) and on mRNA level by RT-qPCR (Fig. S3).

### Recombinant laminin 511-E8 leads to transcriptional changes in cholangiocytes relating to barrier function and inflammation

**Fig. 3:**
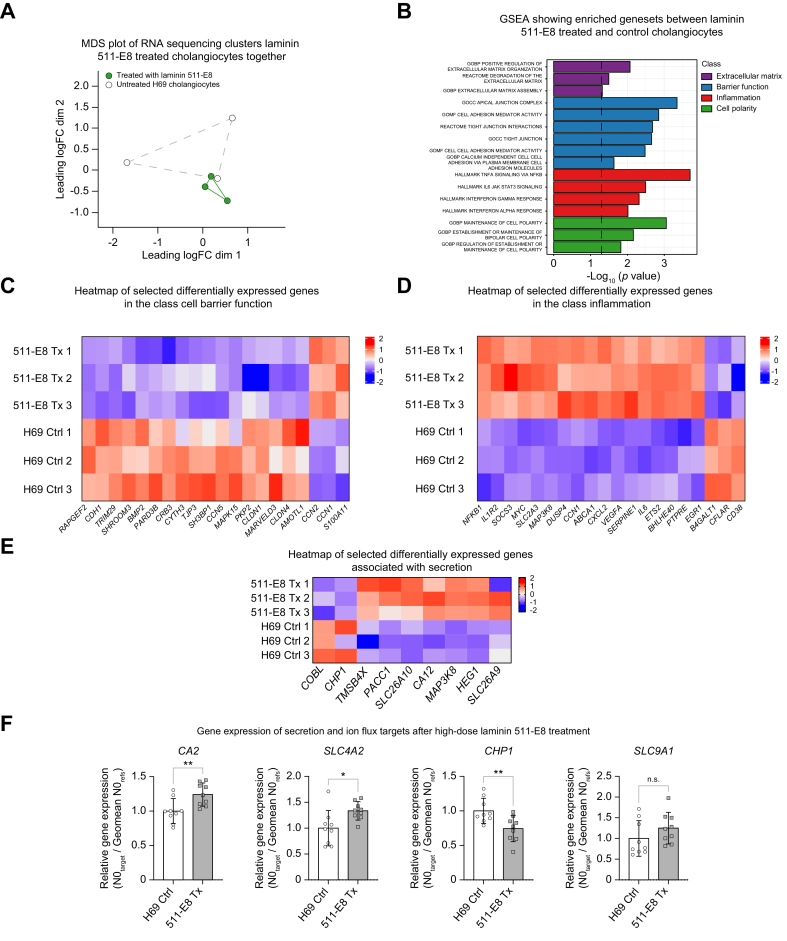
Recombinant laminin 511-E8 treatment transcriptionally alters genes involved in cholangiocellular barrier function, inflammation, and secretion. (A) MDS plot of recombinant laminin 511-E8 (0.25 μg/cm^2^) treated and untreated control cholangiocytes. (B) GSEA displaying enriched gene sets from MSigDB. Heatmaps of (C) differentially expressed genes relating to cell barrier function, (D) inflammation, and (E) secretion. (F) RT-qPCR target genes related to secretion after high-dose (2 μg/cm^2^) recombinant laminin 511-E8. Heatmaps show Log2 transformed z-scored expression values. Levels of significance: (B) -log10 (*p* value) >1.3 was seen as significant. (F) ns = not significant, ∗*p* <0.05, ∗∗*p* <0.01; unpaired *t* test. BP, biological process; CC, cellular component; Ctrl, control; FC, fold change; GO, gene ontology; GSEA, gene set enrichment analysis; MDS, multidimensional scaling; MF, molecular function; MSigDB, molecular signatures database; N0, starting concentration.

To examine the endogenous roles of laminin 511 in human cholangiocytes, RNA sequencing was performed after treatment of human H69 cholangiocytes with recombinant laminin 511-E8. Initial multidimensional scaling (MDS) showed that recombinant laminin 511-E8 treatment clustered the samples together, whereas more spread was observed in the untreated cholangiocytes (Fig. 3A). Gene set enrichment analysis showed that processes related to extracellular matrix, cell polarity, barrier function, and inflammation demonstrated enrichment after recombinant laminin 511-E8 treatment (Fig. 3B–D). Only a limited number of secretory genes were differentially expressed (Fig. 3E). To further investigate a potential relation between laminin 511 and secretion, H69 cholangiocytes were treated with a higher dose (2 μg/cm^2^) of recombinant laminin 511-E8, and gene expression was studied by RT-qPCR. Increased expression was found in the secretory genes *CA2* and *SLC4A2*, while a decrease in *CHP1,* an essential co-factor of *SLC9A1*, was seen (Fig. 3F). Thus, altered gene expression after recombinant laminin 511-E8 treatment implicated roles for laminin 511 in cholangiocellular barrier function, inflammation, and secretory processes.

### Knockdown of laminin 511 constituents increases toxic bile acid permeation and vulnerability to bile acid-induced apoptosis

As secretory proteins are involved in cholangiocellular protection against toxic hydrophobic bile acids, we investigated whether knockdown of the laminin 511 constituents would render cholangiocytes vulnerable to toxic bile acids.

**Fig. 4:**
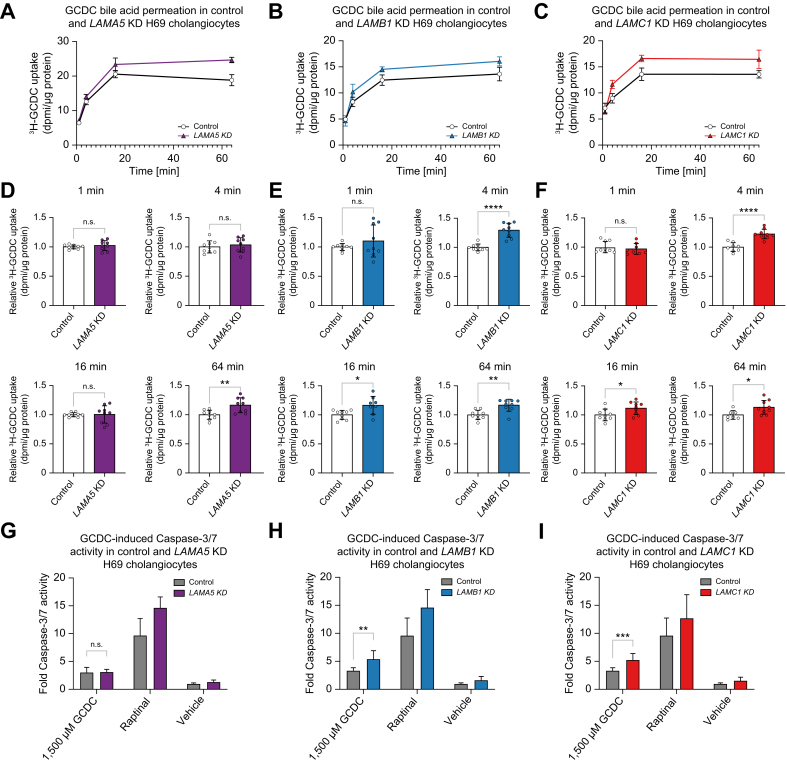
Knockdown of laminin 511 constituents increases toxic bile acid permeation and vulnerability to bile acid-induced apoptosis. ^3^H-GCDC permeation assay in control, (A) *LAMA5*, (B) *LAMB1*, and (C) *LAMC1* knockdown cholangiocytes (representative experiment of n = 3). Relative quantification of ^3^H-GCDC permeation per timepoint in control, (D) *LAMA5*, (E) *LAMB1*, and (F) *LAMC1* knockdown cholangiocytes (seven to nine samples from n = 3 independent experiments). GCDC-induced Caspase-3/7 activity in control (G) *LAMA5*, (H) *LAMB1*, and (I) *LAMC1* knockdown cholangiocytes (nine samples from n = 3 independent experiments). Data are represented as mean with standard deviation. Levels of significance: (D–F) ns = not significant, ∗*p* <0.05, ∗∗*p* <0.01, ∗∗∗∗*p* <0.0001; unpaired t-test. (G–I) ns = not significant, ∗∗*p* <0.01, ∗∗∗*p* <0.001; unpaired *t* test. Control, shRNA control; DPMI, disintegrations per minute; GCDC, glycochenodeoxycholic acid; KD, knockdown; *LAMA5*, laminin alpha 5 chain; *LAMB1*, laminin beta 1 chain; *LAMC1*, laminin gamma 1 chain.

Bile acid permeation assays demonstrated that *LAMB1* and *LAMC1* knockdown cholangiocytes showed an increased GCDC permeation at 4, 16, and 64 min, whereas *LAMA5* knockdown cholangiocytes only showed an increased toxic bile acid permeation at 64 min (Fig. 4A–F). To gain further insight into a potentially altered pH homeostasis induced by the knockdown of laminin 511 constituents, intracellular pH (pHi) measurements were performed. These assays indicated that *LAMA5*, *LAMB1,* and *LAMC1* knockdown cholangiocytes had a lower pHi compared to control H69 cholangiocytes (Fig. S4, Table S3). Baseline intracellular pH tended to decrease in *LAMA5* knockdown cholangiocytes and decreased in *LAMB1* and *LAMC1* cholangiocytes.

The increased permeation of hydrophobic bile acids is expected to result in an increased rate of apoptosis. Caspase-3/7 assays were performed in *LAMA5, LAMB1,* and *LAMC1* knockdown cholangiocytes after exposure to GCDC and Raptinal as a positive control for apoptosis induction. Increased apoptosis was seen in the *LAMB1* and *LAMC1* knockdown cholangiocytes, but not in the *LAMA5* knockdown cholangiocytes (Fig. 4G–I). The *LAMA5* knockdown cholangiocytes also showed the smallest alterations in bile acid permeation and baseline pHi. Thus, knockdown of laminin 511 constituents renders cholangiocytes vulnerable to toxic bile acids.

### Recombinant laminin 511-E8 lowers toxic bile acid permeation and dose-dependently protects against bile acid-induced apoptosis

As a parallel approach to study the role of laminin 511 in human cholangiocytes, cholangiocyte susceptibility to bile acid toxicity was assessed after recombinant laminin 511-E8 treatment.

**Fig. 5:**
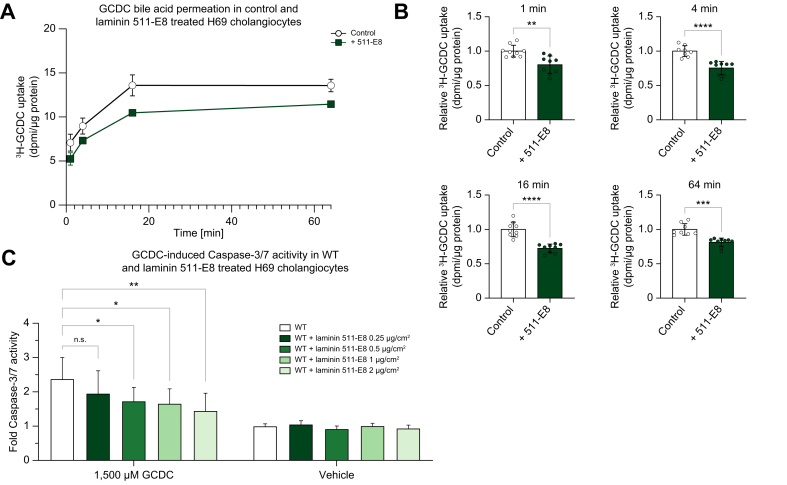
Recombinant laminin 511-E8 lowers toxic bile acid permeation and dose-dependently alleviates bile acid-induced apoptosis. (A) ^3^H-GCDC permeation assay in control and recombinant laminin 511-E8 treated H69 cholangiocytes (representative experiment of n = 3). Relative quantification of ^3^H-GCDC permeation at (B) 1 min, 4 min, 16 min, 64 min in control and recombinant laminin 511-E8 treated H69 cholangiocytes (eight to nine samples from n = 3 independent experiments). (C) GCDC-induced Caspase-3/7 activity in WT and recombinant laminin 511-E8 treated H69 cholangiocytes (nine samples from n = 3 independent experiments). Data are represented as mean with standard deviation. Levels of significance: (B) ∗∗*p* <0.01, ∗∗∗*p* <0.001, ∗∗∗∗*p* <0.0001; unpaired *t* test. (C) ns = not significant, ∗*p* <0.05, ∗∗*p* <0.01; one-way ANOVA. Control, shRNA control; DPMI, disintegrations per minute; GCDC, glycochenodeoxycholic acid; KD, knockdown; WT, wild type.

Recombinant laminin 511-E8 treatment reduced GCDC permeation into H69 cholangiocytes at 1, 4, 16, and 64 min (Fig. 5A and B). Recombinant laminin 511-E8 lowered baseline pHi (Fig. S5, Table S3). Furthermore, recombinant laminin 511-E8 dose-dependently alleviated GCDC-induced apoptosis (Fig. 5C). Thus, recombinant laminin 511-E8 treatment protects cholangiocytes against toxic bile acid permeation and bile acid-induced apoptosis.

### Knockdown of laminin 511 constituents decreases barrier function in human cholangiocytes

**Fig. 6:**
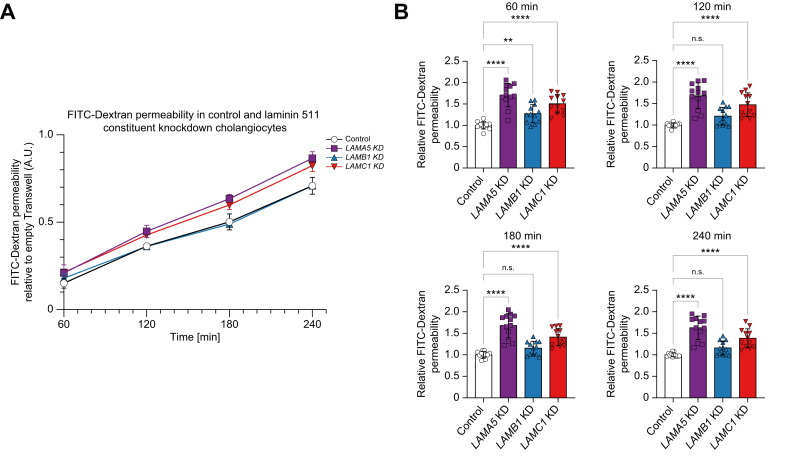
Knockdown of laminin 511 constituents decreases barrier function in human cholangiocytes. (A) Kinetics of FITC–Dextran permeability assay in *LAMA5*, *LAMB1,* and *LAMC1* knockdown compared with control cholangiocytes (representative experiment of n = 4). Relative quantification of FITC–Dextran permeability assay at (B) 60 min, 120 min, 180 min, and 240 min in *LAMA5*, *LAMB1,* and *LAMC1* knockdown compared with control cholangiocytes (12 samples from n = 4 independent experiments). Data are represented as mean with standard deviation. Levels of significance: (B) ns = not significant, ∗∗*p* <0.01, ∗∗∗∗*p* <0.0001; one-way ANOVA. Control, shRNA control; FITC, fluorescein isothiocyanate; KD, knockdown; *LAMB1*, laminin beta 1 chain; *LAMC1*, laminin gamma 1 chain.

To assess whether laminin 511 is involved in cholangiocellular barrier formation, FITC–Dextran permeability assays were performed. *LAMA5* and *LAMC1* KD led to an increase in FITC–Dextran permeability at all time points, whereas *LAMB1* KD only demonstrated a trend towards increased FITC–Dextran permeation (Fig. 6A and B). These data indicate that laminin 511 could play a role in cholangiocellular barrier formation.

### Recombinant laminin 511-E8 enhances barrier function of human cholangiocytes and protects from T lymphocyte-induced barrier dysfunction

**Fig. 7:**
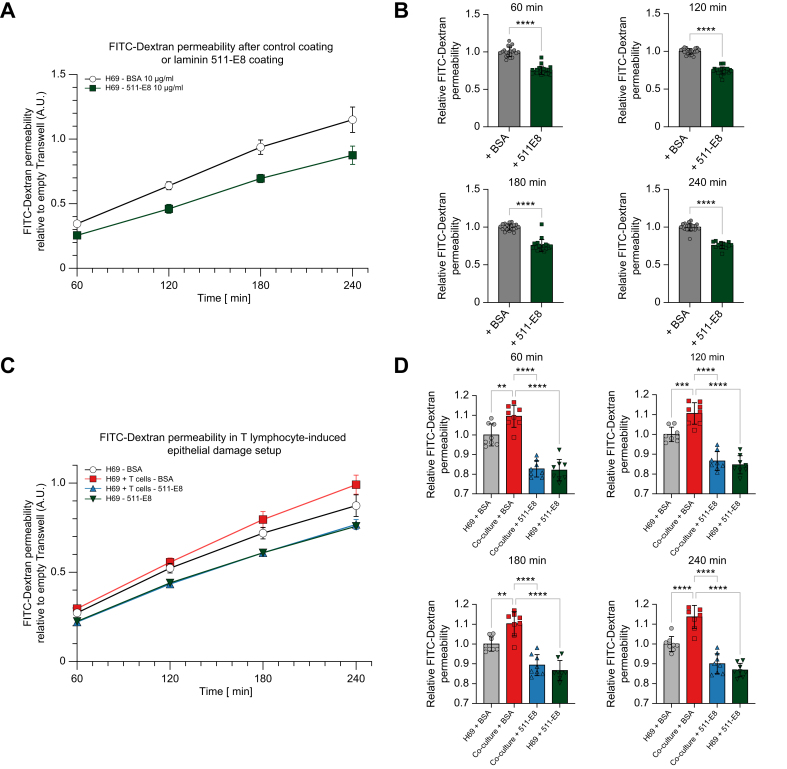
Recombinant laminin 511-E8 enhances cholangiocellular barrier function and protects against T lymphocyte-induced barrier dysfunction. (A) Kinetics of FITC–Dextran permeability assay in cholangiocytes treated with recombinant laminin 511-E8 compared with BSA control coating (representative experiment of n = 4). Relative quantification of FITC–Dextran permeability assay at (B) 60 min, 120 min, 180 min, and 240 min in cholangiocytes treated with recombinant laminin 511-E8 compared with BSA control coating (20 samples from n = 4 independent experiments). (C) Kinetics of FITC-Dextran permeability assay in co-culture set-up of activated T lymphocytes with H69 cholangiocytes (representative experiment of n=3). Relative quantification of FITC–Dextran permeability assay at (D) 60 min, 120 min, 180 min, and 240 min in co-culture set-up (eight samples from n = 3 independent experiments). Data are represented as mean with standard deviation. Levels of significance: (B) ∗∗∗∗*p* = <0.0001; unpaired *t* test. (D) ∗∗*p* <0.01, ∗∗∗*p* <0.001, ∗∗∗∗*p* <0.0001; one-way ANOVA. BSA, bovine serum albumin; FITC, fluorescein isothiocyanate.

As a parallel approach, the effect of recombinant laminin 511-E8 on barrier function was assessed. Recombinant laminin 511-E8 significantly reduced FITC–Dextran permeation (Fig. 7A and B).

To further investigate the role of laminin 511-E8 in the setting of inflammation, H69 cholangiocytes were co-cultured with activated T lymphocytes (Fig. 7C). Co-culture of H69 cholangiocytes with activated T lymphocytes resulted in an increased FITC-Dextran permeability (Fig. 7D, gray *vs.* red). Strikingly, when the H69 monolayer was in basolateral contact with recombinant laminin 511-E8, cholangiocytes were protected from the T lymphocyte-induced barrier dysfunction (Fig. 7D, red *vs.* blue). Notably, the co-culture condition with laminin 511-E8 coating maintained the barrier integrity at the same level as the single H69 cholangiocyte culture condition with laminin 511-E8 coating (Fig. 7D, blue and green). These findings indicate that laminin 511-E8 improves the cholangiocellular barrier function and prevents T lymphocyte-induced barrier dysfunction.

### Laminin 511 and claudin 1 staining may be altered in extrahepatic bile ducts of anti-laminin 511-E8 positive individuals with IRC

**Fig. 8:**
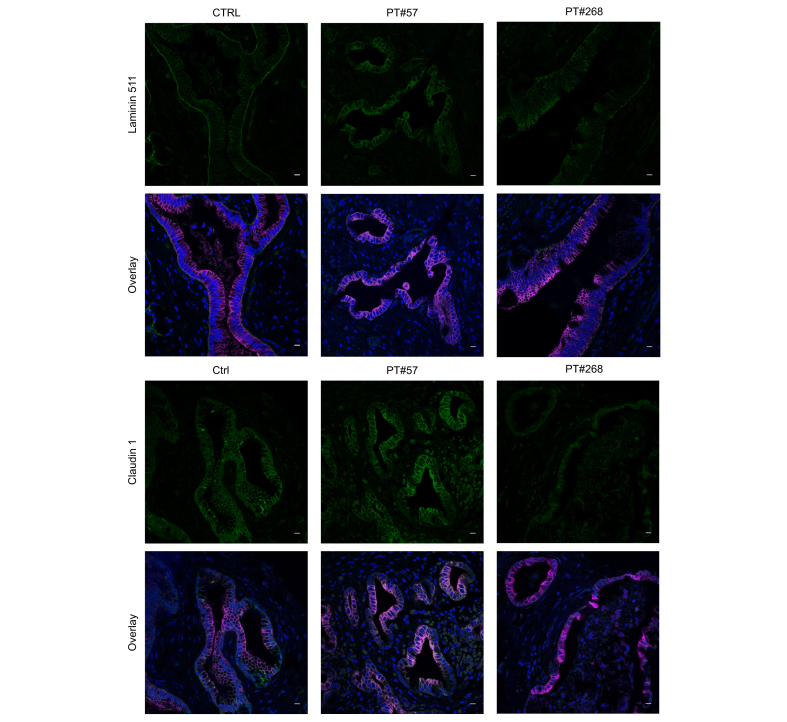
Laminin 511 and claudin 1 staining may be altered in extrahepatic bile ducts of patients with IRC who were anti-laminin 511-E8 positive. Immunofluorescent staining of (top panels) laminin 511 and (bottom panels) claudin 1 alone (green) and as an overlay with cytokeratin 7 (magenta) and Hoechst (blue) in one control and two patients with IRC who were anti-laminin 511-E8 positive (representative images of three fields per condition, Fig. S6 for all fields). Scale bar = 10 μm. CTRL, control; PT, patient.

To investigate the expression and localization of laminin 511 in individuals with IgG4-related cholangitis, laminin 511 expression was assessed by immunofluorescence in extrahepatic bile duct tissue from available resection specimens of controls and patients who were anti-laminin 511-E8 positive. In healthy control tissue, all bile ducts showed a clear basolateral laminin 511 staining (Fig. 8, top panels). In one patient with IRC who was anti-laminin 511-E8 positive (#268), a similar tracing was seen but laminin 511 staining appeared fainter. In the other patient with IRC who was anti-laminin 511-E8 positive (#57) an altered staining pattern was observed with a clear loss of basolateral laminin 511 localization.

Subsequently, extrahepatic bile duct tissue was stained for the tight junction protein claudin 1. Compared with control tissue, one patient with IRC who was anti-laminin 511-E8 positive (#268) showed a fainter and disrupted claudin 1 staining (Fig. 8, bottom panels). In the other patient with IRC who was anti-laminin 511-E8 positive (#57) a clear claudin 1 staining was observed. However, the localization of claudin 1 appeared altered compared with control tissue (as indicated by a higher degree of cytokeratin 7/claudin 1 overlap). All imaged fields can be found in Fig. S6.

## Discussion

The present study is the first to establish that the heterotrimeric extracellular matrix protein laminin 511-E8 is an autoantigen in a subset of patients with IRC. In addition, the present study unravels physiological roles of laminin 511 in human cholangiocytes, the target cells of the immune attack in IRC. Our data indicate that laminin 511 stabilizes cholangiocellular barrier function and protects human cholangiocytes against T lymphocyte-induced barrier dysfunction, toxic bile acid permeation and bile acid-induced apoptosis.

Autoantibodies against laminin 511-E8 were detected in 13.5% of patients with IRC and not in patients with PSC or CCA. Four out of seven patients positive for laminin 511-E8 autoantibodies had a malignancy. This is different from the first report of autoantibodies against laminin 511-E8 in type I AIP where none of the affected individuals with anti-laminin 511-E8 autoantibodies had a malignancy.^8^ The percentage of individuals positive for anti-laminin 511-E8 autoantibodies had been higher in the cohort with type I AIP (51%) than in our IRC cohort (13.5%). This difference could potentially be explained by differences in organ manifestations (pancreas *vs.* bile ducts), the genetic background (Japanese *vs.* Caucasian) and/or environmental risk factors between the two cohorts, but remains unclear at present.

Our experimental data support a protective role of laminin 511 for human cholangiocytes. RNA sequencing implicated laminin 511 to be involved in processes of (i) secretion, (ii) barrier function, and (iii) inflammation. In our RNA sequencing dataset, we were not able to identify driving genes because of the small fold changes after recombinant laminin 511-E8 treatment at 0.25 μg/cm^2^. An explanation could be the degree of variance observed in the MDS plot for untreated H69 cholangiocytes, whereas treatment with laminin 511-E8 clustered the cholangiocytes more closely together. Additionally, in previous studies, higher concentrations of recombinant laminin 511-E8 led to differential expression of secretory genes.^15^^,^^33^ Indeed, treatment with 2 μg/cm^2^ also led to the upregulation of several genes related to secretion in our study (*CA2*, *SLC4A2*). These proteins have been proposed as essential components of the secretory machinery in cholangiocytes.^34^ In turn, these secretory pathways have been hypothesized to create an apical alkaline microenvironment – the biliary bicarbonate umbrella – that protects cholangiocytes against hydrophobic bile acids.^13^^,^^14^ Our data in laminin 511 constituent KD or cell lines demonstrating increased bile acid permeation and increased susceptibility to GCDC-induced apoptosis support such a mechanism. In parallel, recombinant laminin 511-E8 treatment lowered toxic bile acid permeation and dose-dependently alleviated GCDC-induced apoptosis. Still, in the present study, the decrease in intracellular pH in both laminin 511 constituent KD cell lines and in recombinant 511-E8 treated cholangiocytes is incompletely understood. This observation can potentially be explained by the fact that (i) knockdown of one laminin 511 constituent also leads to knockdown of other laminin family members^34^ and (ii) compensatory mechanisms could be at play in knockdown cholangiocytes as this is a stable and permanent strategy.^35^^,^^36^

Our data demonstrating a protective role of laminin 511 in cholangiocellular barrier function align with a previous report showing an altered expression of barrier proteins in IRC-derived cholangiocytes.^17^ This study reported a downregulation of the tight-junction protein claudin 1 and an upregulation of the paracellular pore protein claudin 2. Notably, we observed an altered claudin 1 staining in extrahepatic bile ducts of two patients positive for anti-laminin 511-E8 autoantibodies (Fig. 8). However, definitive conclusions cannot be drawn regarding impaired laminin 511 and claudin 1 expression in IRC, given the limited number of anti-laminin 511-E8-positive individuals with IRC after liver resection available for assessment in our study. In the above-mentioned report,^17^ the downregulation of claudin 1 and upregulation of claudin 2 were related to inflammation and were regulated by IL-4 and IL-13. These cytokines are derived from T cell populations known to be involved in the pathogenesis of IRC.^37^ Notably, in endothelial cells, laminin 511 has been shown to prevent the extravasation of leukocytes and activation of T helper 17 (Th17) cells.^19^^,^^20^^,^^22^^,^[38], [39], [40] These reports fit with our RNA sequencing data suggesting a role for laminin 511 in regulating both cholangiocellular barrier function and inflammation. Indeed, our functional experiments demonstrated that laminin 511 constituent knockdown impaired cholangiocellular barrier function whereas recombinant laminin 511-E8 treatment improved cholangiocellular barrier function and prevented T lymphocyte-induced cholangiocellular barrier dysfunction.

It appears possible that autoantibodies directed against laminin 511-E8 could block its protective cholangiocellular functions – as those in other secretory organs affected by IgG4-RD.^1^^,^^2^ Anti-laminin 511-E8 autoantibodies could potentially contribute to the pathogenesis of IRC by impairing cholangiocellular secretion and barrier function, thereby enhancing the risk of both, T lymphocyte- and bile acid-induced damage. Notably, four out of seven IRC patients with autoantibodies against laminin 511-E8 had IgG4-RD manifestations in other secretory organs that are associated with an impaired barrier function and defective bicarbonate secretion, being the pancreas,^41^^,^^42^ salivary glands,^43^^,^^44^ kidney,^45^ and colon. This makes it tempting to speculate that a decreased epithelial barrier function with attraction of immune cells and impaired bicarbonate secretion because of dysfunction of laminin 511 by autoantibody binding could potentially be a common systemic pathogenic mechanism in a subset of IgG4-RD patients.

In conclusion, our study demonstrates that laminin 511-E8 is an autoantigen in a subset of individuals with IRC. Laminin 511 is located in the extracellular matrix of human cholangiocytes where it promotes cholangiocellular barrier function, prevents T lymphocyte-induced cholangiocellular barrier dysfunction, and protects human cholangiocytes against permeation of toxic bile acids and bile acid-induced apoptosis. Future studies will have to assess whether anti-laminin 511 autoantibodies directly contribute to the pathogenesis of IRC in a subset of affected individuals.

## Financial support

This study was supported by a ZonMw Open Competition grant (to UB), a South African PSC patient foundation grant (Stichting AMC Foundation #20837 to UB), a Gastrostart grant of the Netherlands Society of Gastroenterology (NVGE) and the Netherlands Association for the Study of the Liver (NASL) (to DT and RK), and an Amsterdam UMC/AMC PhD Scholarship (to DT). DT, RK, DTo, AJ, and SvdG have nothing to disclose. UB received lecture and consulting fees from Abacus, Behring, GSK and Zambon.

## Authors’ contributions

Conceived the experiments: DT, RK, SvdG, UB. Performed the experiments: DT, RK, DTo. Analysed the data: DT, RK, DTo, SvdG, UB. Wrote the original draft of the manuscript: DT, RK, UB. Reviewing and editing: all authors. Our RNA sequencing dataset analysis and graphica display: AJ, DT, RK. Approval of the final manuscript: all authors. Funding facilitation: DT, RK, UB.

## Data availability statement

RNA sequencing data were submitted to NCBI Gene Expression Omnibus (GEO) and are freely available under GEO accession number GSE221746.

## Conflicts of interest

The authors declare no conflicts of interest with regard to this work.

Please refer to the accompanying ICMJE disclosure forms for further details.
