# Supplementary material for: Laminin 511-E8, an autoantigen in IgG4-related cholangitis, contributes to cholangiocyte protection
Source: JHEP Rep. 2024 Jan 23;6(4):101015. doi: 10.1016/j.jhepr.2024.101015 (PMC10959701; doi:10.1016/j.jhepr.2024.101015)
Supplement: Multimedia component 1 [file mmc1.docx]

**Laminin 511-E8, an autoantigen in IgG4-related cholangitis, contributes to cholangiocyte protection**

David C. Trampert^*1^, Remco Kersten^*1^, Dagmar Tolenaars^1^, Aldo Jongejan^2^,

Stan F.J. van de Graaf^1^, Ulrich Beuers^1^

* These authors contributed equally to this work and share first authorship

Affiliations

1. Department of Gastroenterology and Hepatology, Tytgat Institute for Liver and Intestinal Research, Amsterdam Gastroenterology Endocrinology Metabolism (AGEM), Amsterdam UMC, Location AMC, University of Amsterdam, Amsterdam, the Netherlands
2. Department of Epidemiology & Data Science, Bioinformatics Laboratory, Amsterdam Public Health Research Institute, Amsterdam UMC, Location AMC, University of Amsterdam, Amsterdam, the Netherlands

**Table of contents**

Supplementary methods p. 3

Supplementary figures

*Fig. S1. Liver parameters and response to therapy* p. 8

*of anti-laminin 511-E8 positive patients*

*Fig. S2. Protein expression of LAMA5, LAMB1 and LAMC1*  p. 10

*in fibroblasts and cholangiocytes*

*Fig. S3. Knockdown validation of laminin 511* p. 11

*constituents LAMA5, LAMB1, LAMC1 by RT-qPCR*

*Fig. S4. Knockdown of laminin 511 constituents in* p. 12

*H69 cholangiocytes decreases baseline intracellular pH*

*Fig. S5. Recombinant laminin 511-E8 treatment of H69* p. 13

*cholangiocytes decreases baseline intracellular pH*

*Fig. S6. All confocal microscopy fields captured of* p. 14

*laminin 511 and claudin 1 immunofluorescent stainings*

*in control and IRC* *extrahepatic bile duct sections*

Supplementary tables

*Table S1. HPB surgery status and histological* p. 21

*assessment of IRC patient cohort*

*Table S2. Human H69 cholangiocyte culture* p. 24

*medium composition for 500ml volume*

*Table S3. Formulation of homemade HBSS for intracellular* p. 25

*pH measurements by BCECF AM under 5% CO2*

References p. 26

**Supplementary methods**

**Descriptive statistics for IRC patient cohort and clinical characteristics of anti-laminin 511-E8 positive patients**

For the entire IRC patient cohort, clinical characteristics were collected from the hospital electronic health record. In addition, for the entire IRC patient cohort, history of HPB surgery and biopsies including pathology reports containing the details for histological IRC assessment were collected from the hospital's electronic health record. Histological IRC diagnosis was “highly suggestive” when 2 out of 3 major criteria were met with >10 IgG4^+^ cells / HPF in resection specimens or biopsies [1-2]. Histological IRC diagnosis was “probable” when 1 out of 3 major criteria was met with >10 IgG4^+^ cells / HPF, otherwise cases had “insufficient evidence”.

For IRC patients who were positive for anti-laminin 511-E8 autoantibodies, detailed collected parameters included gender, age at disease onset, time to diagnosis, organ involvement, profession including > 1 year of potential blue-collar work, positivity to other autoantibodies, liver biochemistry, history of malignancies or major hepatopancreatobiliary surgery and therapeutic management of IgG4-RD. To illustrate the disease courses of anti-laminin 511-E8 positive IRC patients, liver parameters (total bilirubin, ALP, gGT, AST and ALT) and IRC-associated therapy (ERCP, PTC drainage, hepatopancreatobiliary surgery, ursodeoxycholic acid, prednisolone, 6-thioguanine, azathioprine) were extracted from the patient’s electronic health record and graphed accordingly.

**Cell culture and differentiation of LX2**

The human hepatic stellate cell line LX2, described by Xu et al. was treated with 10 ng/ml human TGFβ for 3 days to transdifferentiate cells into activated myofibroblasts as previously described [3-4]. LX2 culture medium consisted of Dulbecco's Modified Eagle Medium (DMEM) supplemented with 10% FBS and 37.5 U/ml (1%) penicillin, 37.5 µg/ml (1%) streptomycin.

**RNA sequencing**

H69 cholangiocytes were seeded at a density of 100.000 cells in 12-well plates and grown for 48 hours to reach confluency. Then treatment commenced with 0.25 µg/cm^2^ recombinant human laminin 511-E8 for 48 hours. Growth media with treatment was refreshed 24 hours after the start of treatment. RNA was isolated from four treated and four untreated wells after 48 hours of treatment using QIAzol lysis reagent (Qiagen) according to the spin column miRNeasy Mini Kit (Qiagen) as previously used for total RNA isolation and downstream bulk RNA sequencing [5]. RNA concentration and quality were assessed by the Core Facility Genomics (Amsterdam UMC) using the RNA ScreenTape. Concentrations were in the range of 100-300 ng/µl and RNA integrity scores (RIN) were 9-10. Three biological replicates with the best RIN score and concentration range were sent in for library preparation by KAPA mRNA Hyperprep. The RNA samples were enriched for mRNA by poly(A) mRNA capture with a library length from 150 – 850 base pairs. Samples were pair-end sequenced (150bp) on the NovaSeq (Illumina) with a sequencing depth of 40 million reads per sample.

Raw FASTQ files were obtained and adapter sequences were trimmed using Trimmomatic v0.39 [6]. Sequences were aligned against the human reference genome (GRCh38) using HISAT2 (v2.2.1) [7]. Quality control was performed using FastQC v0.11.9 and dupRadar v1.12.1 [8]. Count tables were made using HTSeq v0.11.0 [9] and the corresponding GTF. Genes with more than 2 counts-per-million reads (CPM) in 2 or more of the samples were kept. Counts were normalized as previously described [10]. Genes were reannotated using Ensembl (v105) with biomaRt. Count data was transformed to log2-counts per million (logCPM). No clear sample outliers were identified based on library size or normalization factors. MDS plots were generated showing a separation based on the condition with more variation between the control samples than the laminin 511-E8 treated samples. Differential expression was assessed using an empirical Bayes moderated t-test within limma’s linear model framework including the precision weights estimated by voom [11-12]. Cell line effects were corrected for by incorporating it in the design. Benjamini-Hochberg false discovery rate was used to correct for multiple testing of the resulting p-values. Analysis was performed using R v4.1.0 and Bioconductor v3.13. Gene set enrichment analysis (GSEA) was performed using the CAMERA [13] function as implemented in limma (using an inter-gene correlation of 0.01) with gene sets from MSigDB v7.4 (H,C1,C2,C3,C5,C6,C7,C8). Due to spread in the untreated cholangiocyte group and limited effects of low-dose laminin 511-E8 treatment, we made use of p-value instead of adjusted p-value for the GSEA. Heatmaps of selected differentially expressed genes in the classes cell barrier function and inflammation represent the top 20 genes per class, based on the absolute difference in mean expression of the laminin 511-E8 treated and untreated groups. Finally, selected genes of interest associated with secretion are shown. RNA sequencing data was submitted to NCBI Gene Expression Omnibus (GEO) and is freely available under GEO accession number GSE221746.

Single cell RNA sequencing data on the human liver was obtained from GEO accession number GSE124395. Clustering was performed as described ending up with a mature cholangiocyte population (*EPCAM, KRT19, CFTR* +) in clusters 4 and 7, and a hepatocyte population (*ALB, ASGR1* +) in cluster 11 [14]. Within the mesenchymal cell population, periductal (myo)fibroblasts were identified in clusters 9 and 10 based on a recently described murine gene panel consisting of *Col15a1, Cd34, Thy1, Gli1, Clec3b, Fbln2, Entpd2* [15].

**RNA isolation, cDNA synthesis, real-time quantitative PCR, primer design and validation**

Total RNA was isolated from H69 cholangiocytes using TRIzol reagent (Sigma). With chloroform and centrifugation, the aqueous phase was isolated. RNA was recovered by precipitating with isopropyl alcohol. RNA pellets were washed with 70% ethanol and resuspended in diethylpyrocarbonate (DEPC) treated water. Quality (A260/A280 >1.8) and concentration of RNA samples were checked by spectrophotometry using the Nanodrop 1000 (Thermo Scientific, Waltham, MS).

2 μg of RNA was used as input and treated with DNase I (Promega) followed by reverse transcription into cDNA using Oligo-dT (Invitrogen), Random Hexamer primers (Promega) and Revertaid transcriptase (Fermentas) resulting in 20 μl cDNA. cDNA was diluted to 100 μl, after which 2 μl served as a template for real-time quantitative PCR with the SensiFAST SYBR No-ROX kit (Bioline). RT-qPCR plates were run on the Bio-Rad CXF96. Raw fluorescent values were exported and starting concentration (N0) and cycle quantification (Cq) values were obtained using LinRegPCR v.2013.0 (Academic Medical Center, Amsterdam) [16]. Expression levels were normalized to the geomean of human *36B4* (*RPLP0*) and *HPRT* reference genes.

Primers used for RT-qPCR were self-designed to cover all transcript variants of the gene of interest. To this end FASTA sequences of all transcript variants were obtained and aligned with a nucleotide count using Clustal Omega [17], a multiple sequence alignment tool (<https://www.ebi.ac.uk/Tools/msa/clustalo/>). NCBI PRIMER-BLAST was then used to design primers restricted to the sequence range identical for all transcript variants (<https://www.ncbi.nlm.nih.gov/tools/primer-blast/index.cgi>) [18]. Additional requirements were that primers must span an exon-exon junction and ideally the primer pair was separated by at least one intron on the corresponding genomic DNA. Subsequently primers were validated by assessing the linear relation between increasing cycle quantification (Cq) values with serial dilution of cDNA. Melting temperatures (Tm) of the designed primers were checked after the RT-qPCR run with a melting curve analysis. Finally, the amplified PCR product was loaded on an agarose gel and separated by electrophoresis alongside a standard ladder to confirm the size of the amplified product corresponded with the size of the target PCR product.

**Intracellular pH measurement by 2',7'-bis-(2-carboxyethyl)-5-(and-6)-carboxyfluorescein acetoxymethyl ester (BCECF AM)**

The principle of this assay is performed as previously described with minor custom changes [19]. H69 cholangiocytes were plated in 96-well black solid flat bottom tissue culture treated plates (Corning, Costar #3916) and grown until confluency. For one 96-well plate a working solution of 5 µM BCECF AM (Sigma, Invitrogen B1150) was prepared in 5 ml of 20 mM HEPES-buffered Hank’s Balanced Salt Solution (HBSS) without phenol red at pH 7.4. After a series of washing steps with HEPES-buffered HBSS, cells were loaded with BCECF AM for 45 minutes at room temperature and protected from light. Three wells per condition / cell line were left unloaded for blank measurements. Excessive dye was removed and the plate was incubated for an additional 30 minutes in HEPES-buffered HBSS.

A pH standard was prepared from a high-potassium Nigericin (10 µM) solution supplemented with 30 mM HEPES covering the pH range from 5.8 to 8.6. The pH standards were placed in a water bath at 37 degrees Celsius, after which the pH was measured of each standard. BCECF AM loaded wells from the 96-well plate were filled with the pH standards and experimental solutions were added to the loaded and blank wells of each condition. The plate was placed in the CLARIOstar (BMG LABTECH, Ortenberg, Germany) set at 37 degrees and a gain adjustment for all wells was performed aiming for 50% of maximal fluorescent values. Ratiometric fluorescent measurement was performed at dual excitation wavelength: λEx1= 490-10 nm (pH-sensitive) / λEx2= 440-10 nm (pH‐insensitive), emission was collected at λEm= 535-20 nm. pH standards were measured until a steady state was acquired. After this, experimentally treated wells were measured at 37 degrees with 5% CO_2_ inflow again until a steady state was acquired.

Raw data of the experiment were converted to fluorescent ratios. Prior to this, background fluorescence from the unloaded blank wells were subtracted from the corresponding BCECF AM loaded conditions at all time points. The fluorescent ratio R was calculated as F490/440. Fluorescent ratios were converted to a true pH using the fluorescent ratios from the high-potassium Nigericin pH standards. In brief, pH values were obtained by performing nonlinear least-square fitting according to the following equation as previously described [20-21]: $pH=pK_{a}-\log\frac{R- R_{A}}{R_{B}-R} \times\frac{F_{A(\lambda2)}}{F_{B(\lambda2)}}$ , where R stands for F490/440, R_A_ and R_B_ represent fluorescent ratios at extreme acidic and basic conditions respectively, F_A(λ2)_ and F_B(λ2)_ are the fluorescent values λEx2 440 nm at extreme acidic and basic conditions respectively. Baseline intracellular pH values were determined by averaging the pH values of the last eight timepoints.

**Supplementary figures**

**
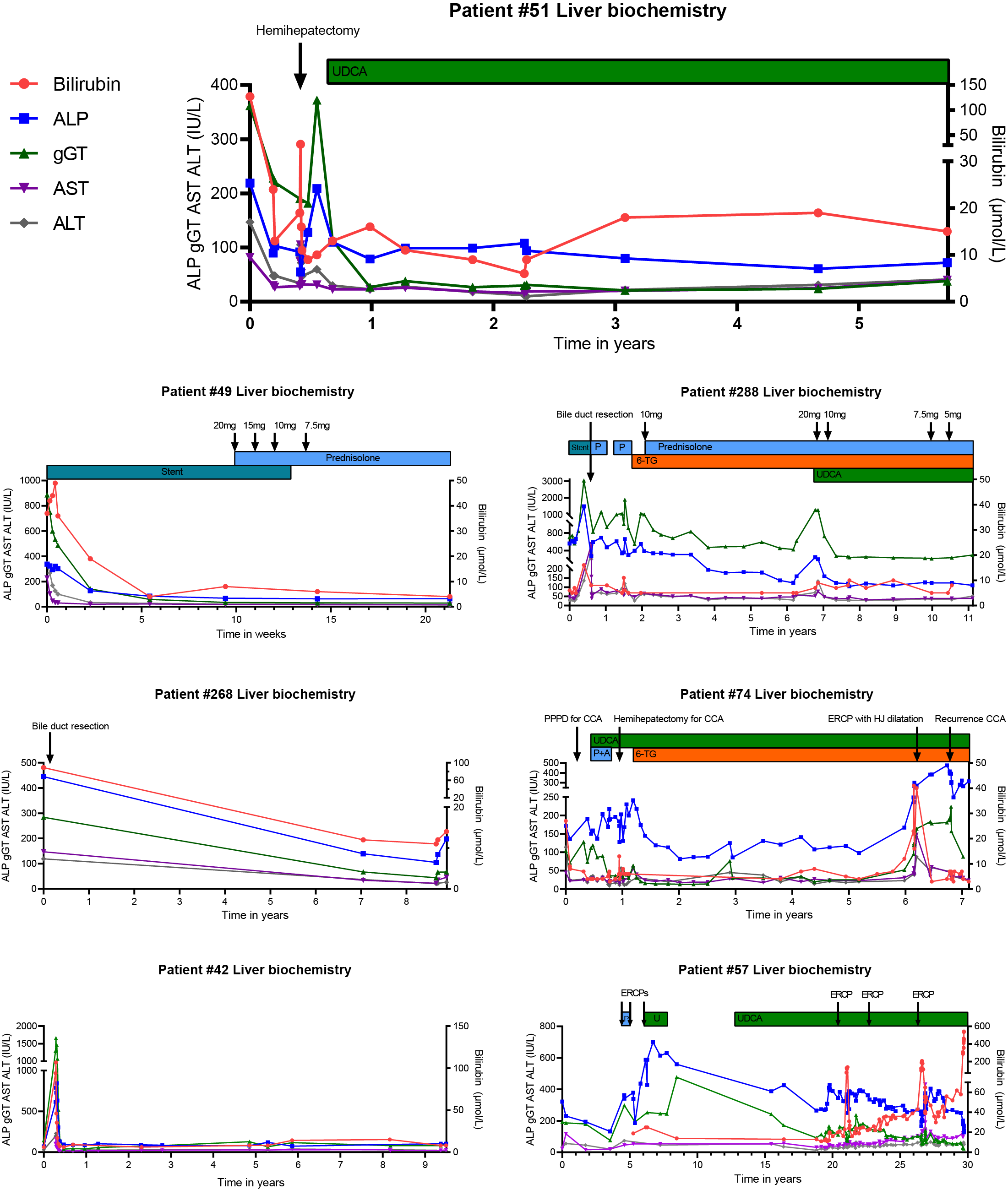
**

**Fig. S1. Liver biochemistry and response to IRC directed therapy in patients positive for anti-laminin 511-E8 autoantibodies.** ALP, gGT, AST, ALT left Y-axis. Bilirubin right Y-axis. Therapeutic interventions are indicated with bars and arrows above the graphs. Patient # corresponds to ELISA patient numbers in Figure 1 of the main manuscript. Abbreviations: 6-TG, 6-thioguanine; A, azathioprine; ALP, alkaline phosphatase; ALT, alanine aminotransferase; AST, aspartate aminotransferase; CCA, cholangiocarcinoma; ERCP, endoscopic retrograde cholangiopancreaticography; gGT, gamma-glutamyl transferase; HJ, hepaticojejunostomy; P, prednisolone; PPPD, pylorus-preserving pancreatoduodenectomy; UDCA, ursodeoxycholic acid.

**
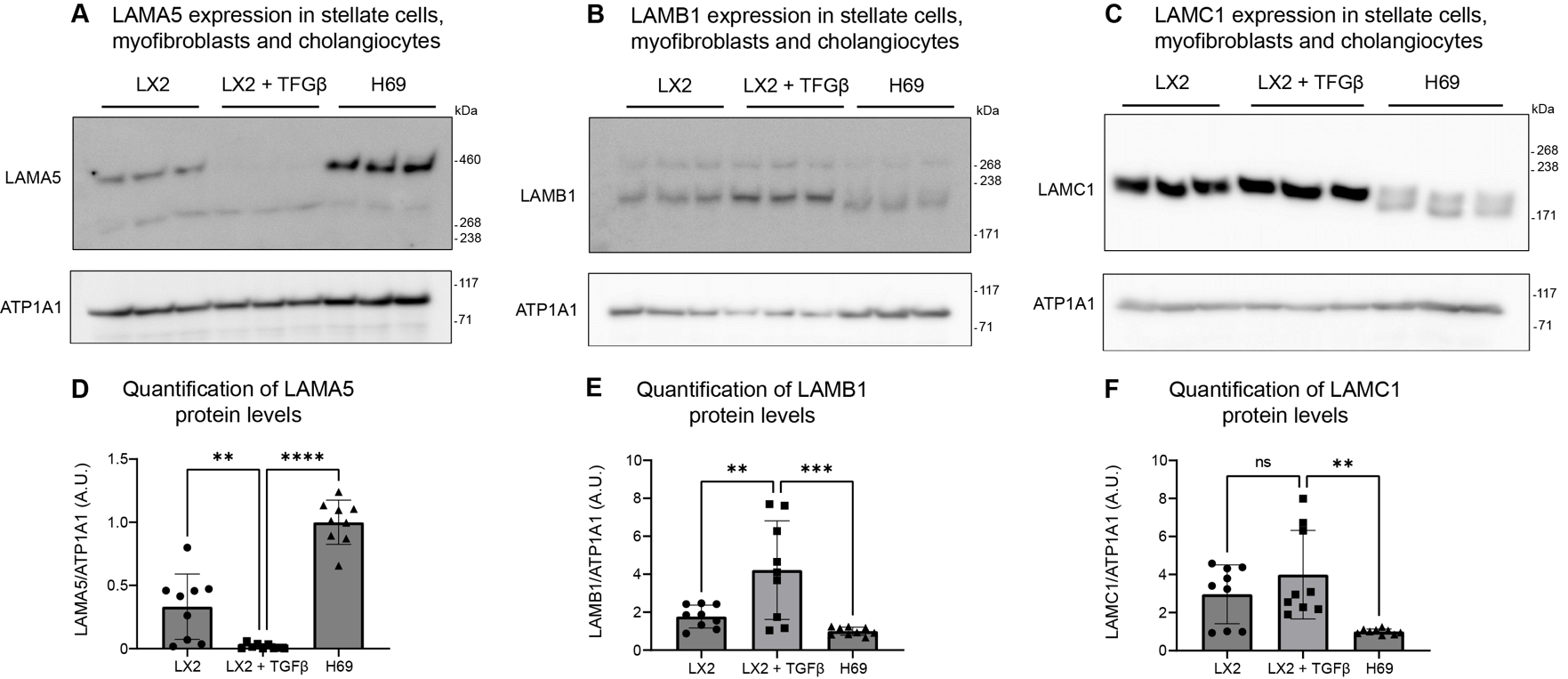
**

**Fig. S2. Laminin 511 constituent protein expression in fibroblast models and cholangiocytes.** (A) LAMA5 (400 kDa), (B) LAMB1 (198 kDa), and (C) LAMC1 (178 kDa) expression in naive LX2 (stellate cells), activated LX2 (myofibroblasts), and H69 cholangiocytes. Quantification of (D) LAMA5, (E) LAMB1, and (F) LAMC1 protein levels normalized by ATP1A1 (112 kDa), (9 cell samples from n=3 independent experiments). Data are represented as mean with standard deviation. Levels of significance: (E) – (F) ns = not significant, ** p < 0.01, *** p < 0.001, **** p < 0.0001. unpaired t-test.

**
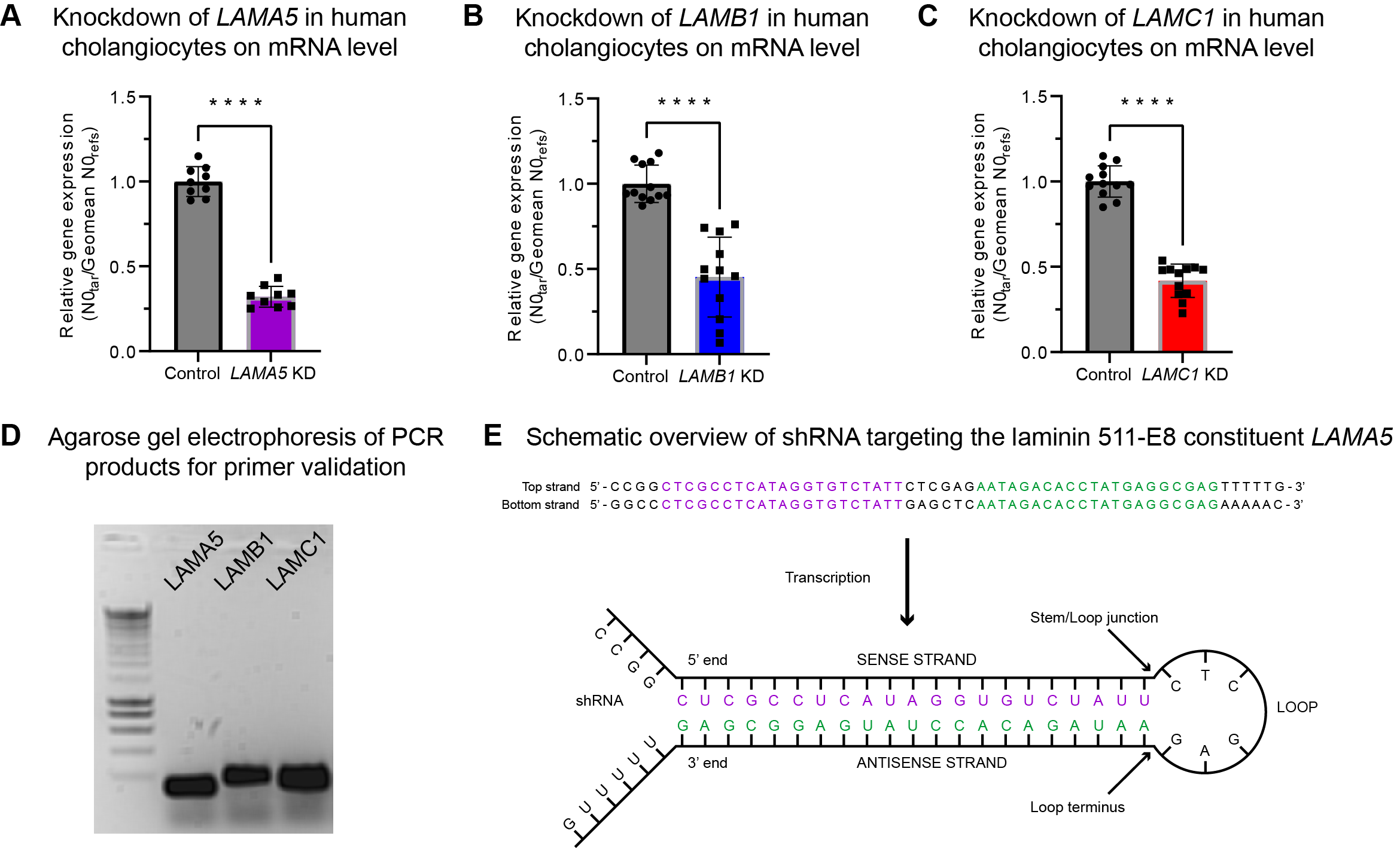
**

**Fig. S3. Knockdown validation of laminin 511 constituents *LAMA5*, *LAMB1*, *LAMC1* by RT-qPCR.** Relative mRNA expression in (A) *LAMA5*, (B) *LAMB1*, and (C) *LAMC1* shRNA knockdown human H69 cholangiocytes compared to control cholangiocytes (9-12 cell samples from n=3-4 independent experiments). (D) gel electrophoresis showing PCR products of *LAMA5*, *LAMB1*, and *LAMC1* as part of RT-qPCR primer validation. (E) Schematic of short hairpin RNA with the used sequence of *LAMA5* in place*.* Data are represented as starting concentration (N0) of target genes over the geomean of the reference genes *36B4* (*RPLP0*) and *HPRT* N0 values normalized to control. Levels of significance: (A) – (C) **** p < 0.0001. (A) – (C) unpaired t-test. Abbreviations: Control, shRNA control; KD, knockdown; N0, starting concentration; shRNA, short hairpin RNA.

**
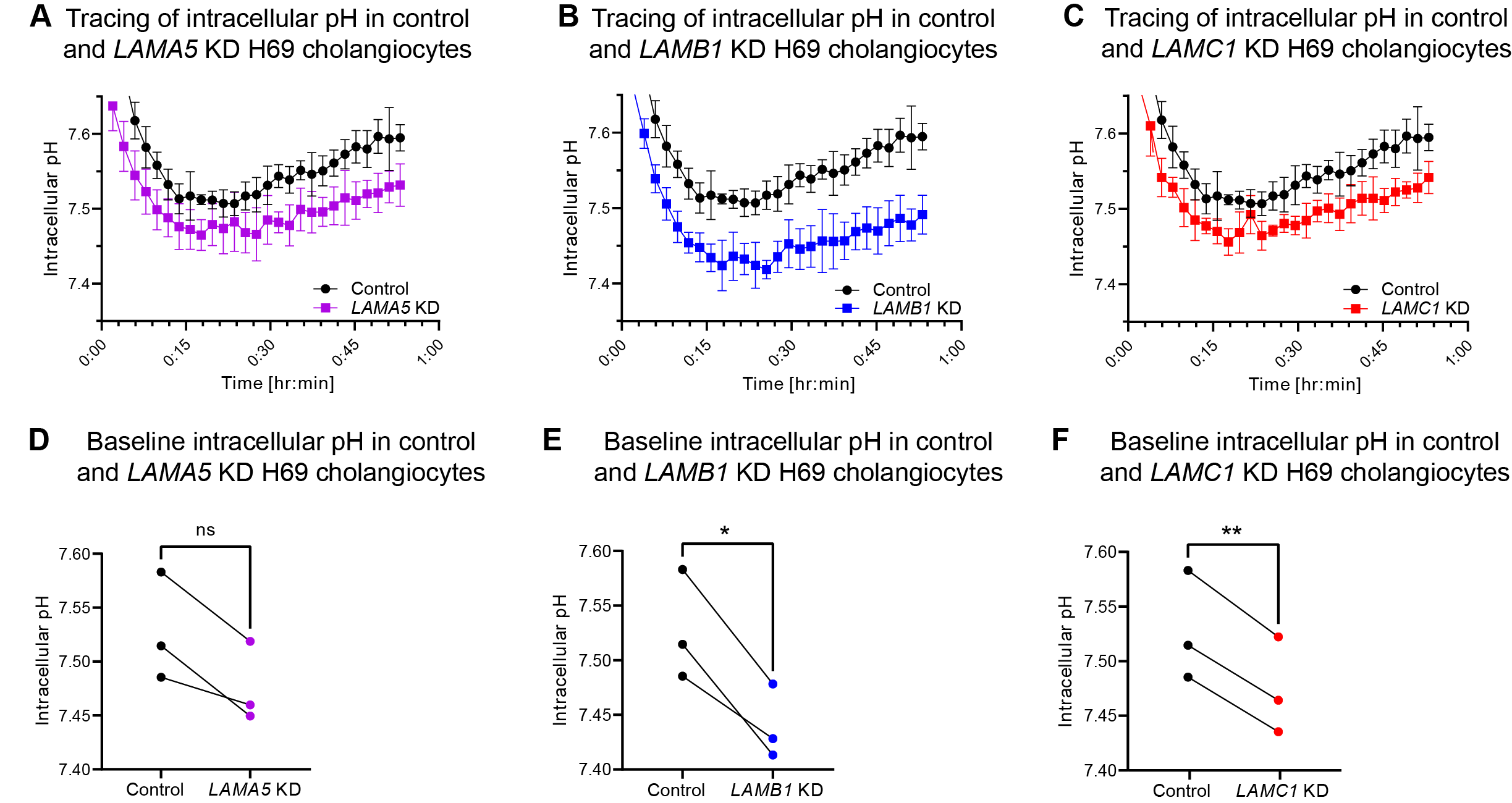
**

**Fig. S4. Knockdown of laminin 511 constituents in H69 cholangiocytes decreases baseline intracellular pH.** Intracellular pH tracing in control, (A) *LAMA5*, (B) *LAMB1,* and (C) *LAMC1* knockdown H69 cholangiocytes (representative experiment of n=3). Baseline intracellular pH in control, (D) *LAMA5*, (E) *LAMB1,* and (F) *LAMC1* knockdown cholangiocytes (3 average baseline values from n=3 independent experiments). Data are represented as mean with standard deviation. Levels of significance: (D) – (F) ns = not significant, * p < 0.05, ** p < 0.01. (D) – (F) paired t-test. Abbreviations: Control, shRNA control; KD, knockdown.

**
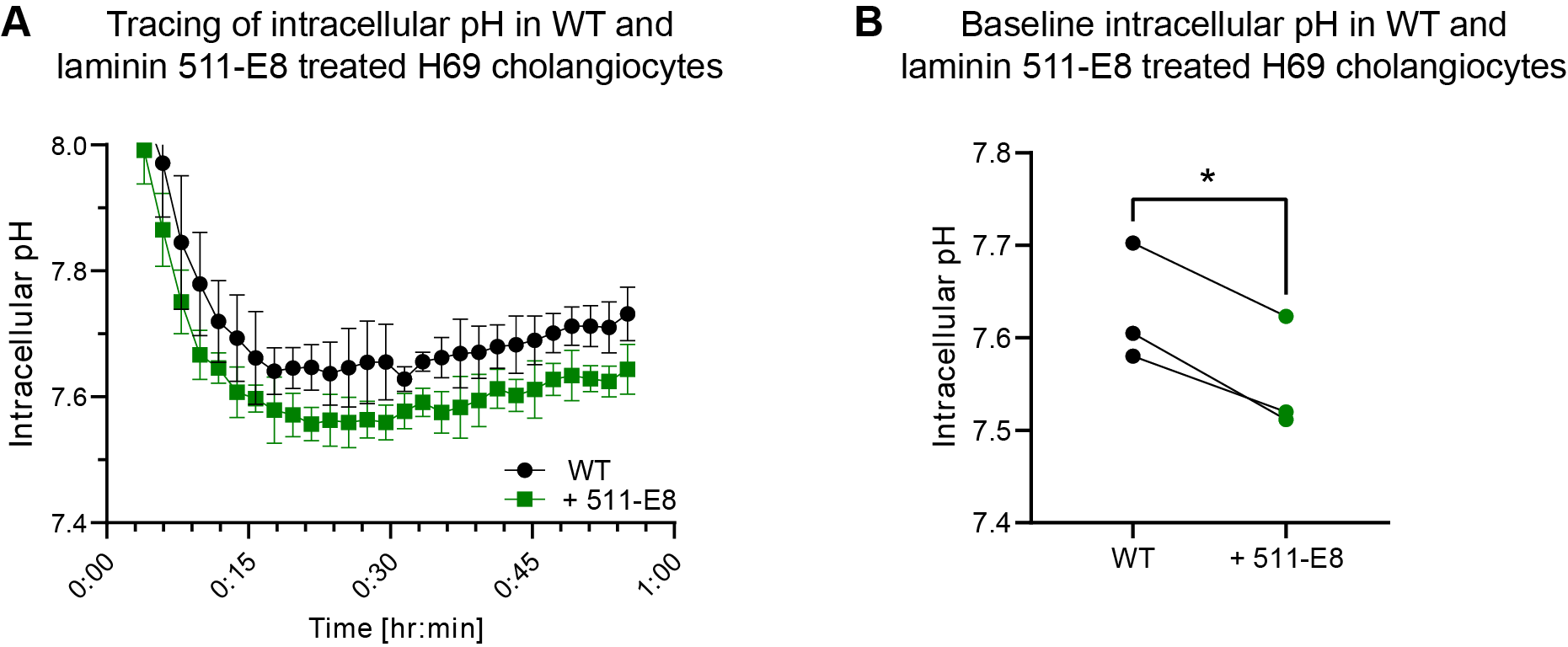
**

**Fig. S5. Recombinant laminin 511-E8 treatment of H69 cholangiocytes decreases baseline intracellular pH.** (A) Intracellular pH tracing in wild-type and recombinant laminin 511-E8 treated H69 cholangiocytes (representative tracing from n=3 independent experiments). (B) Baseline intracellular pH in wild-type and recombinant laminin 511-E8 treated H69 cholangiocytes (3 average baseline values from n=3 independent experiments). Data are represented as mean with standard deviation. Levels of significance: (B) * p < 0.05. (B) paired t-test. Abbreviations: KD, knockdown; WT, wild-type.

**
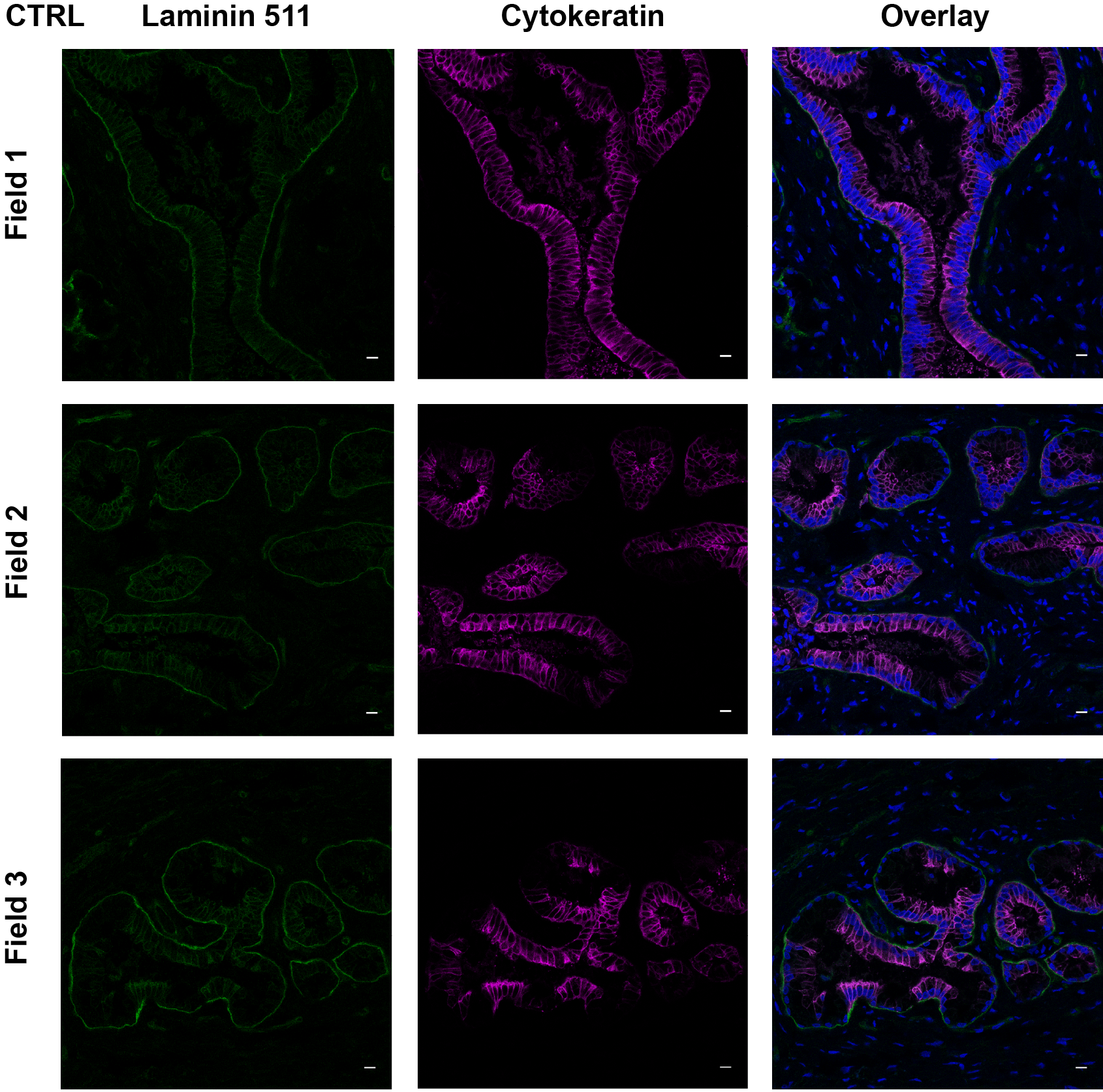
**

**
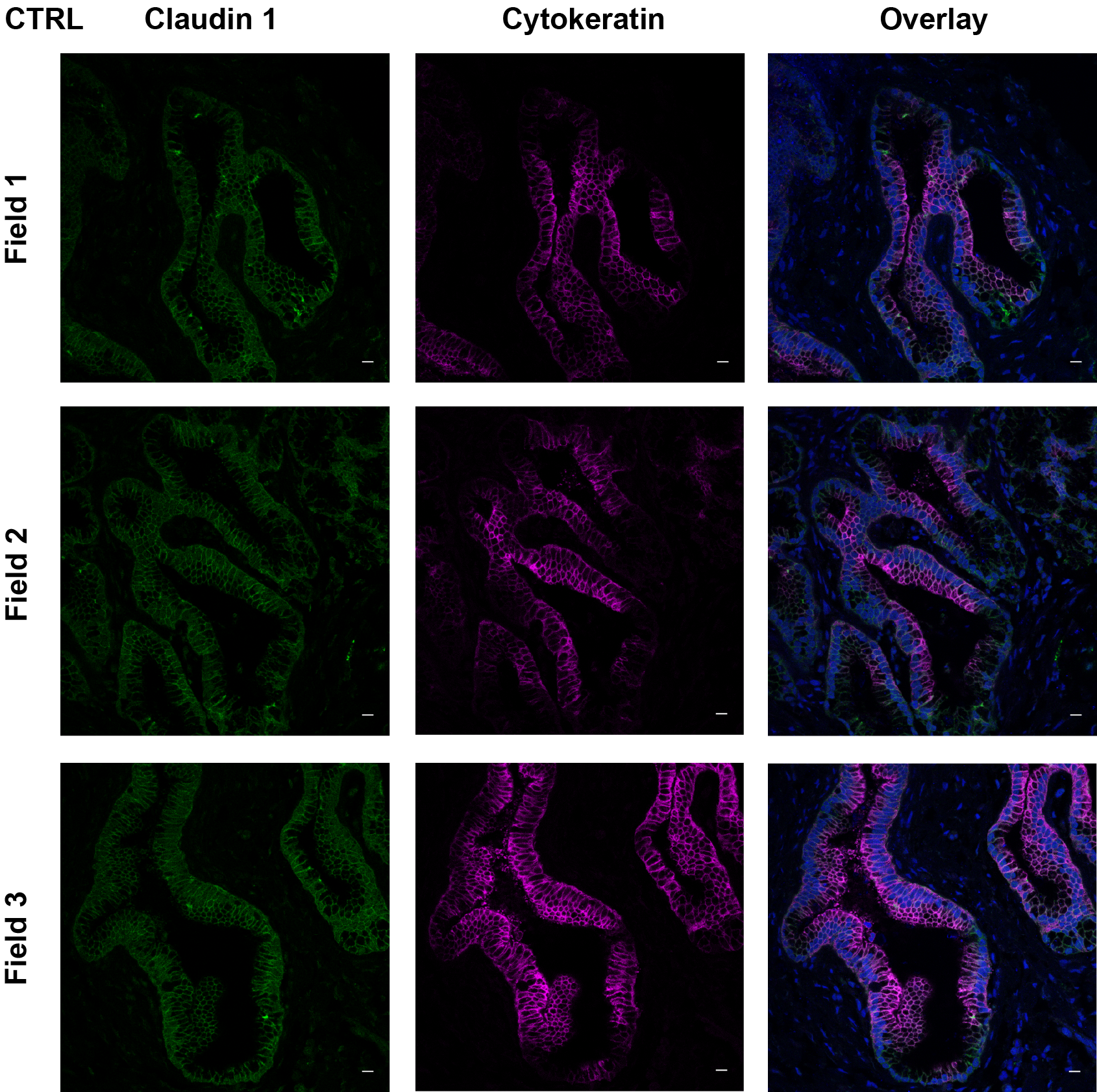
**

**
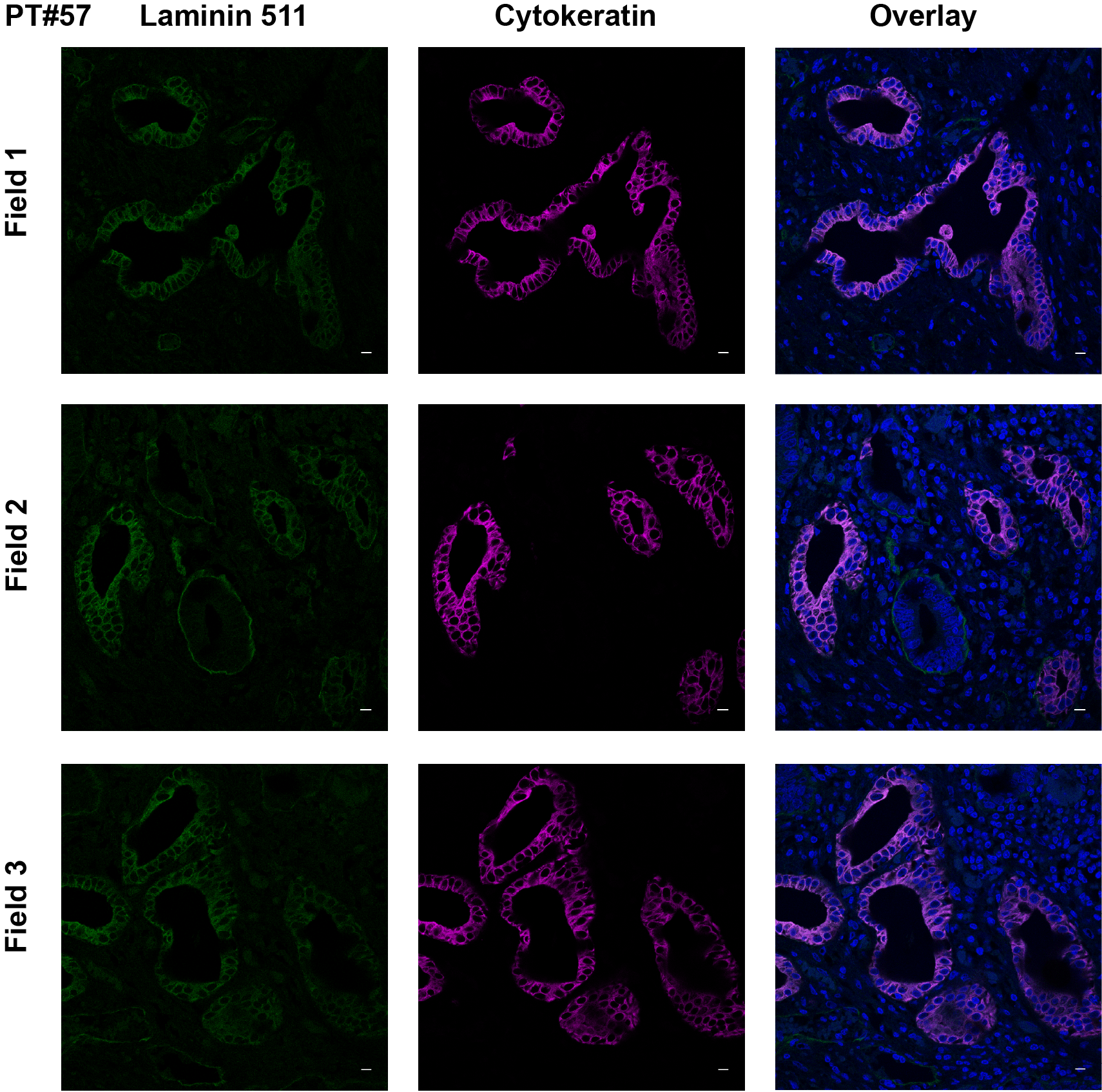
**

**
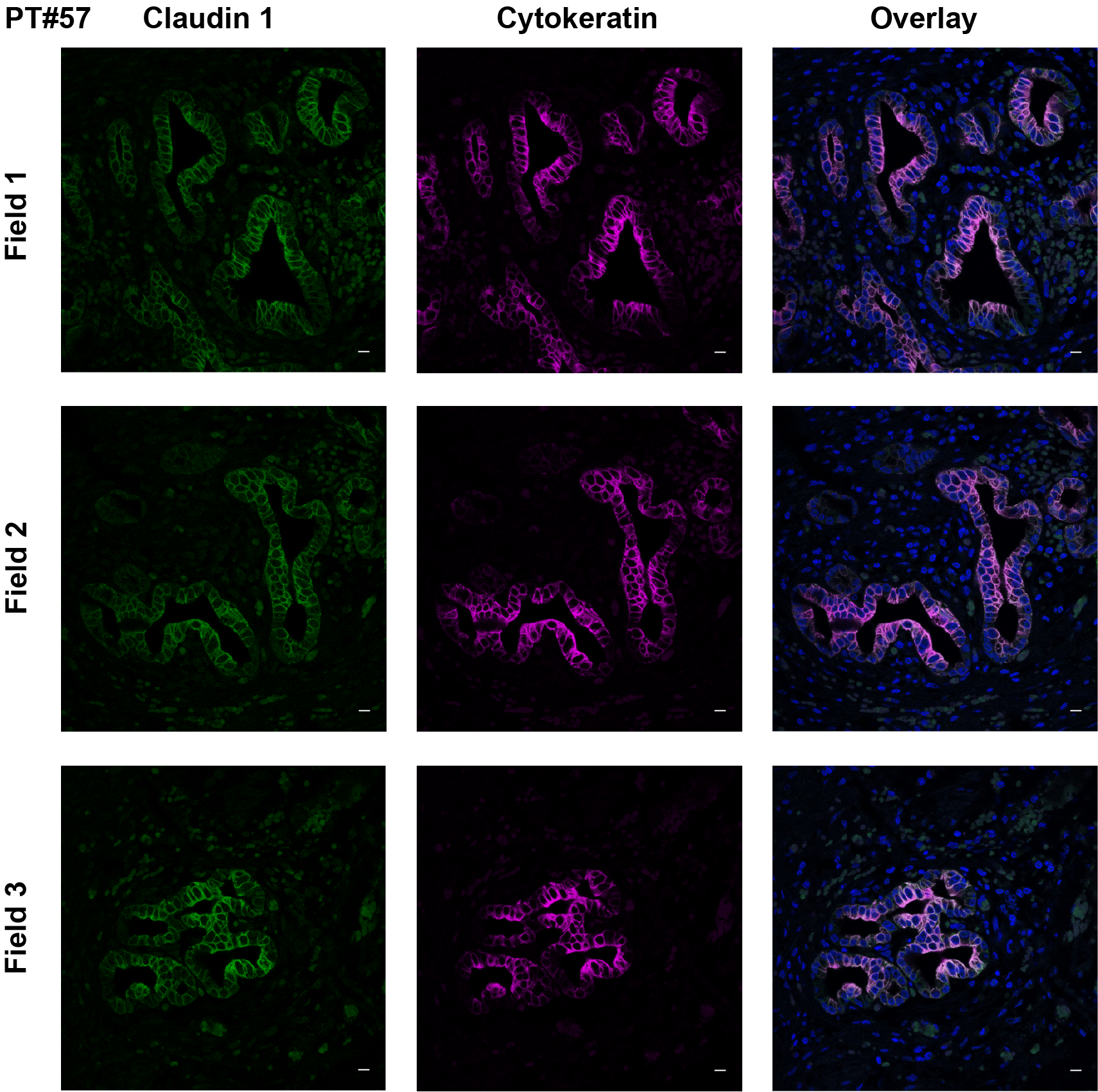
**

**
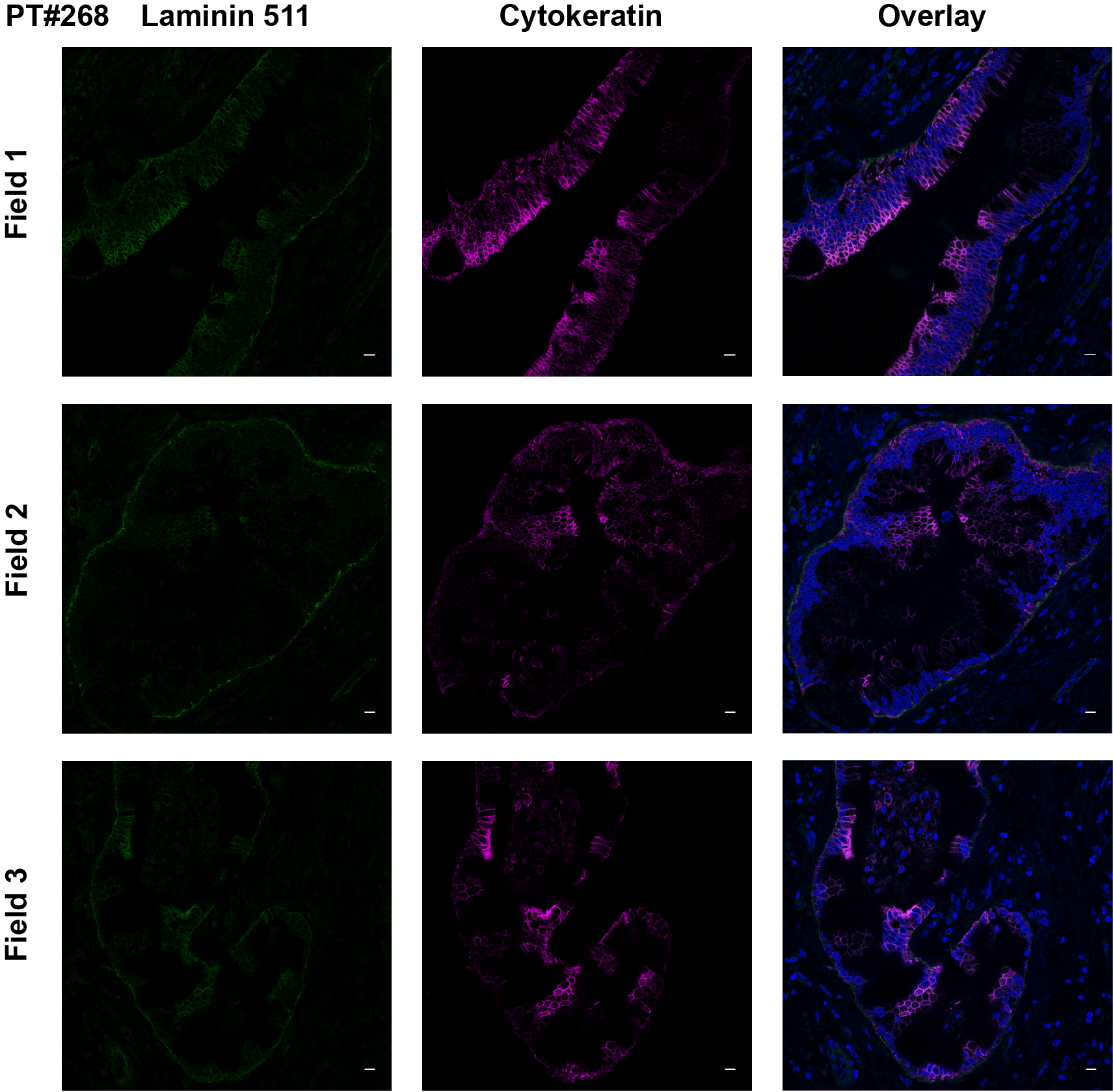
**

**
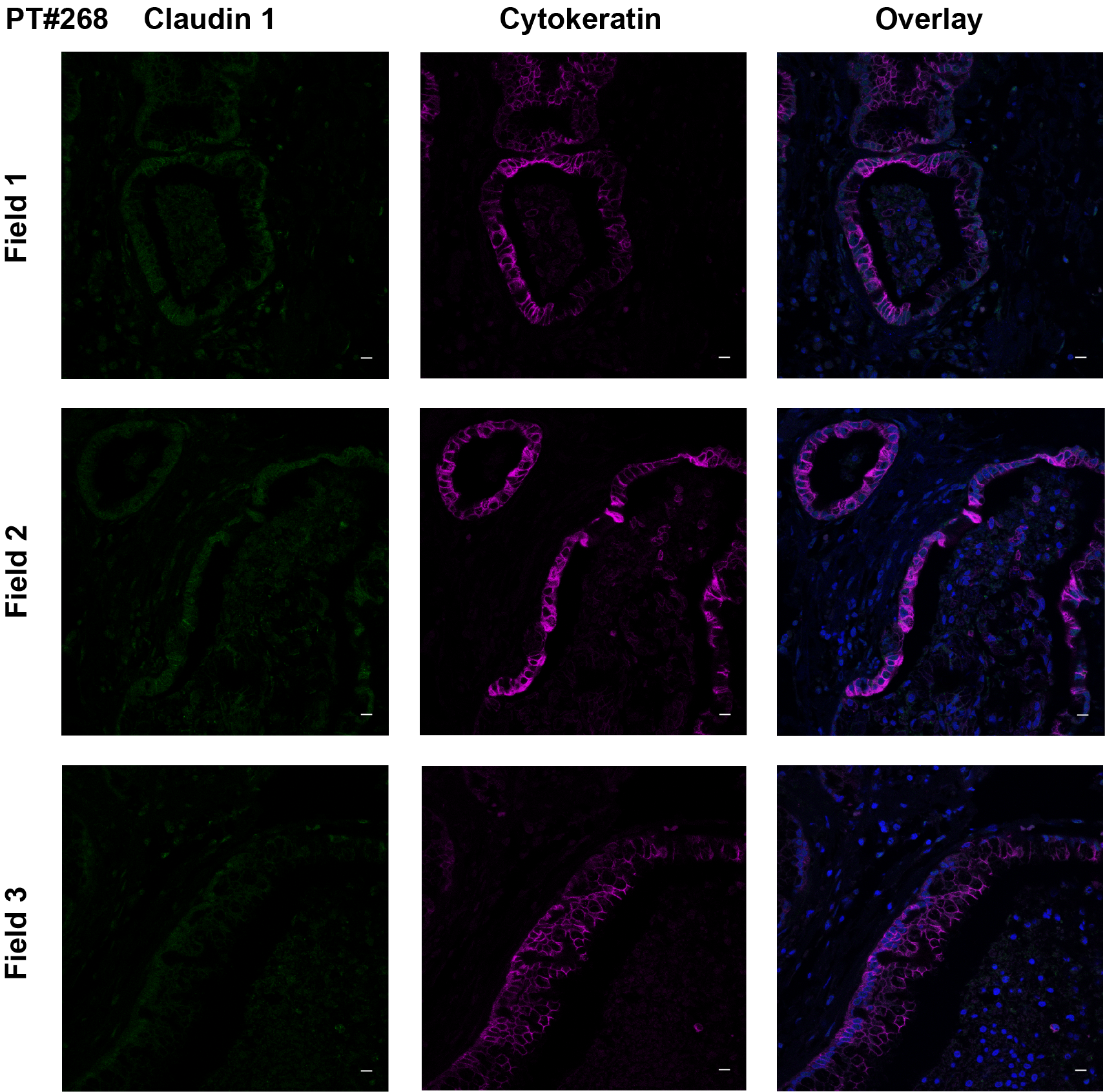
**

**Fig. S6. Immunofluorescent stainings of laminin 511 and claudin 1 in extrahepatic bile duct sections of control and of IRC patients #57 and #268.** In a blinded experiment, three fields of each condition were imaged with a confocal microscope using identical imaging settings. In green laminin 511 and claudin 1 are displayed, in magenta a cytokeratin 7 staining is displayed to identify the biliary tissue. The overlay image contains a nuclear Hoechst staining. Note that there is one structure in field 2 of patient #57 showing a basolateral laminin 511 staining, which is negative for cytokeratin 7, likely being a vascular structure. Representative fields were used for Figure 8 of the main manuscript**.** Scale bar is 10 μm. Abbreviations: CTRL, control; PT, patient.

**Supplementary tables**

**Table S1. Surgical history and histological assessment in IRC patient cohort**

| **Patient #** | **511-E8 positivity** | **HPB surgery** | **Timing of HPB surgery** | **HPB**  **Biopsy** | **Indication for surgery/biopsy** | **Histological IRC criteria**  **assessed** | **IRC Histological assessment** | **Lympho-**  **Plasmacellular infiltrate** | **Obliterative phlebitis** | **Storiform fibrosis** | **IgG4^+^/HPF** |
| --- | --- | --- | --- | --- | --- | --- | --- | --- | --- | --- | --- |
| **74** | **Yes** |  |  | Liver biopsy | Suspected CCA with PSC | Yes | Highly suggestive | Yes | No | Yes | >10 |
|  |  | PPPD | Before diagnosis |  | Suspected CCA | Yes | Highly suggestive | Yes | No | Yes | >10 |
|  |  | Hemihepatectomy | After diagnosis |  | CCA in background of IRC | No | NA | NA | NA | NA | NA |
| **51** | **Yes** | Hemihepatectomy | Before diagnosis | No | Suspected CCA | Yes | Highly suggestive | Yes | Yes | Yes | >20 |
| **288** | **Yes** |  |  | Liver biopsy | Suspected CCA | No | NA | NA | NA | NA | NA |
|  |  | Bile duct resection | Before diagnosis | No | Suspected CCA | Yes | Highly suggestive | Yes | Yes | Yes | >10 |
| 21 | No |  |  | Liver biopsy | Suspected CCA | Yes | Insufficient evidence | Yes | No | No | <10 |
|  |  | Hemihepatectomy | Before diagnosis |  | Suspected CCA | Yes | Highly suggestive | Yes | No | Yes | >10 |
| 47 | No | Hilar resection | Before diagnosis | No | Suspected CCA | Yes | Highly suggestive | Yes | No | Yes | >75 |
| 44 | No | Hemihepatectomy | Before diagnosis | No | Suspected CCA | Yes | Highly suggestive | Yes | No | Yes | 52 |
| 50 | No |  |  | Liver biopsy |  | No | NA | NA | NA | NA | NA |
|  |  | Cholecystectomy | Before diagnosis |  | Suspected CCA | Yes | Highly suggestive | Yes | No | Yes | >50 |
| 148 | No | Hemihepatectomy | Before diagnosis | No | Suspected CCA | Yes | Highly suggestive | Yes | No | Yes | 20 |
| 225 | No | Hemihepatectomy | Before diagnosis | No | Suspected CCA | Yes | Highly suggestive | Yes | Yes | Yes | 100 |
| 269 | No |  |  | Liver biopsy | Suspected CCA | No | NA | NA | NA | NA | NA |
|  |  | Bile duct resection | Before diagnosis |  |  | Yes | Highly suggestive | Yes | No | Yes | 22 |
| 262 | No | Hilar resection | Before diagnosis | No | Suspected CCA | Yes | Highly suggestive | Yes | No | Yes | 13 |
| 271 | No | Hemihepatectomy | Before diagnosis | No | Suspected CCA | Yes | Highly suggestive | Yes | No | Yes | 13 |

| **Patient #** | **511-E8 positivity** | **HPB surgery** | **Timing of HPB surgery** | **HPB**  **Biopsy** | **Indication for surgery/biopsy** | **Histological IRC criteria**  **assessed** | **IRC Histological assessment** | **Lympho-**  **plasmacellular infiltrate** | **Obliterative phlebitis** | **Storiform fibrosis** | **IgG4^+^/HPF** |
| --- | --- | --- | --- | --- | --- | --- | --- | --- | --- | --- | --- |
| 22 | No | No | NA | Liver biopsy | Elevated liver enzymes e.c.i. | Yes | Probable IRC | No | No | Yes | >10 |
| 52 | No | Biliodigestive anastomosis | Before diagnosis | No | Suspected pancreatic carcinoma | Yes | Probable IRC | Yes | No | No | >50 |
| **268** | **Yes** | Bile duct resection | Before diagnosis | No | Suspected CCA | Yes | Probable IRC | Yes | No | Yes | 8 |
| 58 | No | No | NA | Liver biopsy | Suspected metastasis, unknown primary tumour | Yes | Insufficient evidence* | Yes | No | Yes | Unreliable staining |
| 4 | No | No | NA | Liver biopsy | Suspected CCA,  DDx IRC | Yes | Insufficient evidence | No | No | No | Unreliable staining |
| 28 | No | No | NA | Liver biopsy | Liver cirrhosis e.c.i. | Yes | Insufficient evidence* | Yes | No | Yes | Not enough material |
| 24 | No | No | NA | Ampulla of Vater biopsy | Suspected CCA, DDx IRC | Yes | Insufficient evidence | No | No | No | <10 |
| 95 | No | No | NA | Liver biopsy | Elevated liver enzymes e.c.i. | No | NA | NA | NA | NA | NA |
|  |  |  |  | Liver biopsy | Elevated liver enzymes, DDx IRC | Yes | Insufficient evidence | NA | NA | NA | <10 |
| 54 | No | No | NA | Ampulla of Vater biopsy | Bile duct stenosis, DDx IRC | Yes | Insufficient evidence | No | No | No | None |
| 270 | No | Hemihepatectomy | Before diagnosis | No | Suspected CCA | Yes | Insufficient evidence | Yes | No | No | 3 |
| 75 | No | No | NA | Liver biopsy | Elevated liver enzymes e.c.i. | Yes | Insufficient evidence | No | No | No | None |
| 233 | No | No (autopsy) | After diagnosis | No | NA | Yes | Insufficient evidence | Yes | No | No | 13 |
| 53 | No | Hemihepatectomy | After diagnosis | NA | Liver abscess | NA | NA | NA | NA | NA | NA |
| 6 | No | Biliodigestive anastomosis | Before diagnosis | No | Suspected pancreatic tumour | NA | NA | NA | NA | NA | NA |
| 223 | No | Biliodigestive anastomosis | Before diagnosis | No | Suspected pancreatic tumour | NA | NA | NA | NA | NA | NA |
|  |  | PPPD | Before diagnosis | No | Suspected pancreatic tumour | NA | NA | NA | NA | NA | NA |
| 5 | No | No | NA | Liver biopsy | Suspected CCA | No | NA | NA | NA | NA | NA |
| 11 | No | No | NA | No | NA | NA | NA | NA | NA | NA | NA |

| **Patient #** | **511-E8 positivity** | **HPB surgery** | **Timing of HPB surgery** | **HPB**  **Biopsy** | **Indication for surgery/biopsy** | **Histological IRC criteria**  **assessed** | **IRC Histological assessment** | **Lympho-**  **plasmacellular infiltrate** | **Obliterative phlebitis** | **Storiform fibrosis** | **IgG4^+^/HPF** |
| --- | --- | --- | --- | --- | --- | --- | --- | --- | --- | --- | --- |
| **57** | **Yes** | Cholecystectomy | Before diagnosis |  | Cholecystolithiasis | No | NA | NA | NA | NA | >20 |
|  |  |  |  | Liver biopsy | Elevated liver enzymes e.c.i. | No | NA | NA | NA | NA | NA |
|  |  |  |  | Liver biopsy | DDx PSC, study biopsy | No | NA | NA | NA | NA | NA |
|  |  |  |  | Liver biopsy | DDx PSC, study biopsy | No | NA | NA | NA | NA | NA |
| 80 | No | No | NA | Ampulla of Vater biopsy | Suspected metastasis esophageal carcinoma | No | NA | NA | NA | NA | NA |
| 65 | No | No | NA | No | NA | NA | NA | NA | NA | NA | NA |
| 67 | No | No | NA | No | NA | NA | NA | NA | NA | NA | NA |
| 87 | No | No | NA | No | NA | NA | NA | NA | NA | NA | NA |
| 144 | No | No | NA | No | NA | NA | NA | NA | NA | NA | NA |
| 147 | No | No | NA | No | NA | NA | NA | NA | NA | NA | NA |
| 158 | No | No | NA | No | NA | NA | NA | NA | NA | NA | NA |
| 160 | No | No | NA | No | NA | NA | NA | NA | NA | NA | NA |
| 170 | No | No | NA | No | NA | NA | NA | NA | NA | NA | NA |
| 172 | No | No | NA | No | NA | NA | NA | NA | NA | NA | NA |
| 157 | No | No | NA | No | NA | NA | NA | NA | NA | NA | NA |
| 17 | No | No | NA | No | NA | NA | NA | NA | NA | NA | NA |
| 177 | No | No | NA | No | NA | NA | NA | NA | NA | NA | NA |
| 213 | No | No | NA | No | NA | NA | NA | NA | NA | NA | NA |
| 311 | No | No | NA | No | NA | NA | NA | NA | NA | NA | NA |
| 257 | No | No | NA | No | NA | NA | NA | NA | NA | NA | NA |
| 263 | No | No | NA | No | NA | NA | NA | NA | NA | NA | NA |
| **49** | **Yes** | No | NA | No | NA | NA | NA | NA | NA | NA | NA |
| **42** | **Yes** | No | NA | No | NA | NA | NA | NA | NA | NA | NA |
| 23 | No | No | NA | No | NA | NA | NA | NA | NA | NA | NA |
| 39 | No | No | NA | No | NA | NA | NA | NA | NA | NA | NA |
| 9 | No | No | NA | No | NA | NA | NA | NA | NA | NA | NA |

**Table S1.** * IRC histological assessment was scored as “insufficient evidence” due to a lack of adequate material for IgG4^+^ staining. Abbreviations: CCA, cholangiocarcinoma; DDx, differential diagnosis; e.c.i., e causa ignota; HPB, hepatopancreatobiliary; HPF, high power field; IRC, IgG4-related cholangitis; NA, not applicable; PSC, primary sclerosing cholangitis; PPPD, pylorus-preserving pancreatoduodenectomy.

**Table S2. Human H69 cholangiocyte culture medium composition for 500 ml volume**

| Material / Compound | Cat. No. | Supplier | Volume / Concentration |
| --- | --- | --- | --- |
| DMEM | 31600-083 | Gibco | 375 mL |
| Ham’s F-12 Nutrient Mix | N6760 | Sigma | 125 mL |
| Sodium Bicarbonate | 144-55-8 | Merck | 1.794 g/L |
| HEPES-NaOH, pH 7.4 | H3375 | Sigma | 20 mM |
| L-glutamine | BE-17-605E | Lonza | 3.25 mM |
| Penicillin / Streptomycin | 15140-122 | Gibco | 37.5 U/ml / 37.5 µg/ml |
| Fetal Bovine Serum | S00EW10003  Batch: BDC-S00EW1 | Bodinco | 10% (v/v) |
| Adenine | A2786 | Sigma | 180 µM |
| Insulin | I1882 | Sigma | 865 nM |
| Transferrin | T8158 | Sigma | 62.5 nM |
| Triiodothyronine (T3) | T3697 | Sigma | 2 nM |
| Hydrocortisone | H0888 | Sigma | 1.1 µM |
| Epinephrine | E4250 | Sigma | 5.5 µM |
| Epidermal Growth Factor | E9644 | Sigma | 1.67 nM |

**Table S3. Formulation of homemade HBSS for intracellular pH measurements by BCECF AM under 5% CO_2_**

| Component | Final concentration (mM) | | Osmolarity (mosm/L) |
| --- | --- | --- | --- |
| CaCl_2_ * 2H_2_O | 1.27 | 3.80 | |
| KCl | 4.56 | 9.12 | |
| NaH_2_PO_4_ * 2H_2_O | 0.45 | 0.90 | |
| MgSO_4_ * 7H_2_O | 0.41 | 0.81 | |
| MgCl_2_ * 6H_2_O | 0.39 | 1.18 | |
| NaCl | 107.80 | 215.61 | |
| NaHCO_3_^-^ | 21.43 | 42.85 | |
| Na_2_HPO_4_ * 2H_2_O | 0.34 | 1.01 | |
| HEPES-NaOH, pH 7.4 | 20 | 30 | |
| Glucose | 5.55 | 5.55 | |

**References**

*Author names in bold designate shared co-first authorship*.

1. **Roos E, Hubers LM**, Coelen RJS, Doorenspleet ME, de Vries N et al. IgG4-Associated Cholangitis in Patients Resected for Presumed Perihilar Cholangiocarcinoma: a 30-Year Tertiary Care Experience. Am J Gastroenterol. 2018; 113: 765-772. doi: 10.1038/s41395-018-0036-5.
2. Deshpande V, Zen Y, Chan JKC, Yi EE, Sato Y et al. Consensus statement on the pathology of IgG4-related disease. Mod Pathol. 2012; 25: 1181-92. doi: 10.1038/modpathol.2012.72.
3. Xu L, Hui AY, Albanis E, Arthur MJ, O'Byrne SM et al. Human hepatic stellate cell lines, LX-1 and LX-2: new tools for analysis of hepatic fibrosis. Gut. 2005; 54: 142-51. doi: 10.1136/gut.2004.042127.
4. Smith-Cortinez N, Fagundes RR, Gomez V, Kong D, de Waart DR et al. Collagen release by human hepatic stellate cells requires vitamin C and is efficiently blocked by hydroxylase inhibition. FASEB J. 2021; 35: e21219. doi: 10.1096/fj.202001564RR.
5. Verstegen MMA, Roos FJM, Burka K, Gehart H, Jager M, de Wolf M et al. Human extrahepatic and intrahepatic cholangiocyte organoids show region-specific differentiation potential and model cystic fibrosis-related bile duct disease. Sci Rep. 2020; 10: 21900. doi: 10.1038/s41598-020-79082-8.
6. Bolger AM, Lohse M, Usadel B. Trimmomatic: a flexible trimmer for Illumina sequence data. Bioinformatics. 2014; 30: 2114-20. doi: 10.1093/bioinformatics/btu170.
7. Kim D, Langmead B, Salzberg SL. HISAT: a fast spliced aligner with low memory requirements. Nat Methods Nat Methods. 2015; 12: 357-60. doi: 10.1038/nmeth.3317.
8. Sayols S, Scherzinger D, Klein H. dupRadar: a Bioconductor package for the assessment of PCR artifacts in RNA-Seq data. BMC Bioinformatics. 2016; 17: 428. doi: 10.1186/s12859-016-1276-2.
9. Anders S, Pyl PT, Huber W. HTSeq-A Python framework to work with high-throughput sequencing data. Bioinformatics. 2015; 31: 166-9. doi: 10.1093/bioinformatics/btu638.
10. Robinson MD, Oshlack A. A scaling normalization method for differential expression analysis of RNA-seq data. Genome Biol. 2010; 11: R25. doi: 10.1186/gb-2010-11-3-r25.
11. **Robinson MD, McCarthy DJ**, Smyth GK. edgeR: A Bioconductor package for differential expression analysis of digital gene expression data. Bioinformatics. 2010; 26: 139-40. doi: 10.1093/bioinformatics/btp616.
12. Ritchie ME, Phipson B, Wu D, Hu Y, Law CW, Shi W et al. Limma powers differential expression analyses for RNA-sequencing and microarray studies. Nucleic Acids Res. 2015; 43: e47. doi: 10.1093/nar/gkv007.
13. Wu D, Smyth GK. Camera: a competitive gene set test accounting for inter-gene correlation. Nucleic Acids Res. 2012; 40: e133. doi: 10.1093/nar/gks461.
14. Aizarani N, **Saviano A, Sagar**, Mailly L, Durand S, Herman JS et al. A human liver cell atlas reveals heterogeneity and epithelial progenitors. Nature. 2019; 572: 199-204. doi: 10.1038/s41586-019-1373-2.
15. Lei L, Bruneau A, El Mourabit H, Guégan J, Folseraas T, Lemoinne S et al. Portal fibroblasts with mesenchymal stem cell features form a reservoir of proliferative myofibroblasts in liver fibrosis. Hepatology. 2022; 76 :1360-1375. doi: 10.1002/hep.32456.
16. Ramakers C, Ruijter JM, Deprez RHL, Moorman AFM. Assumption-free analysis of quantitative real-time polymerase chain reaction (PCR) data. Neurosci Lett 2003;339:62–6. https://doi.org/10.1016/s0304-3940(02)01423-4
17. **Madeira F, Pearce M**, Tivey ARN, Basutkar P, Lee J, Edbali O et al. Search and sequence analysis tools services from EMBL-EBI in 2022. Nucleic Acids Res. 2022; 50: W276-W279. doi: 10.1093/nar/gkac240.
18. Jian Ye J, Coulouris G, Zaretskaya I, Cutcutache I, Rozen S, Madden TL. Primer-BLAST: a tool to design target-specific primers for polymerase chain reaction. BMC Bioinformatics. 2012; 13: 134. doi: 10.1186/1471-2105-13-134.
19. Chang JC, Go S, de Waart DR, Munoz‐Garrido P, Beuers U, Paulusma, C, Oude Elferink, R. Soluble Adenylyl Cyclase Regulates Bile Salt‐Induced Apoptosis in Human Cholangiocytes. Hepatology. 2016; 64: 522-34. doi: 10.1002/hep.28550.
20. Brown AM, A step-by-step guide to non-linear regression analysis of experimental data using a Microsoft Excel spreadsheet. Comput Methods Programs Biomed. 2001; 65:191-200. doi: 10.1016/s0169-2607(00)00124-3.
21. Kemmer G, Keller S. Nonlinear least-squares data fitting in Excel spreadsheets. Nat Protoc. 2010; 5: 267-81. doi: 10.1038/nprot.2009.182.
