# Supplementary material for: Laminin 511-E8, an autoantigen in IgG4-related cholangitis, contributes to cholangiocyte protection
Source: JHEP Rep. 2024 Jan 23;6(4):101015. doi: 10.1016/j.jhepr.2024.101015 (PMC10959701; doi:10.1016/j.jhepr.2024.101015)
Supplement: Multimedia component 2 [file mmc2.docx]

**JHEP Reports**

**CTAT methods**

Tables for a “Complete, Transparent, Accurate and Timely account” (CTAT) are now mandatory for all revised submissions. The aim is to enhance the reproducibility of methods.

- Only include the parts relevant to your study
- Refer to the CTAT in the main text as ‘Supplementary CTAT Table’
- Do not add subheadings
- Add as many rows as needed to include all information
- Only include one item per row

**If the CTAT form is not relevant to your study, please outline the reasons why:**

|  |
| --- |

- 1. **Antibodies**

| **Name** | **Citation** | **Supplier** | **Cat no.** | **Clone no.** |
| --- | --- | --- | --- | --- |
| ATP1A1; ATPase Na+/K+ Transporting Subunit Alpha | Koenderink JB et al. J Biol Chem. 2003; 278: 51213-22 | Gift from JB Koenderink |  |  |
| LAMA5; Laminin Alpha 5 | Gordon-Weeks A et al. Cancers. 2019; 11: 630 | Novus Biologicals | NBP2-42391 | CL3118 |
| LAMB1; Laminin Beta 1 | Chen Q et al. Neuro Oncol. 2014; 16: 637-51 | Santa Cruz | sc-17810 | A-1 |
| LAMC1; Laminin Gamma 1 | Ye G et al. Cancer Biol Ther. 2019; 20: 711-19 | Santa Cruz | sc-17751 | D-3 |
| Laminin 511 | Komiya et al. Cancer Med. 2014; 3: 537-49 | GeneTex | GTX17688 | 12D |
| Claudin 1 | Enhanced validation antibody,  Human Prot. Atlas | Sigma-Aldrich, Prestige Antibodies® Powered by Atlas Antibodies | HPA048319 | Polyclonal |
| Cytokeratin 7 | Yoon et al. Nat Commun. 2022; 13: 3291 | Abcam | ab181598 | EPR17078 |
| Cytokeratin 7 |  | Santa Cruz | sc-8421 | C46 |
| Goat anti-mouse IgG Alexa Fluor 488 | Lopez-Escalera et al. Biochem Biophys Rep. 2022; 31: 101314 | Molecular Probes | A-11001 |  |
| Goat anti-rabbit IgG Alexa Fluor 488 | Ghosh et al. Theranostics. 2022; 12: 5574–5595 | Molecular Probes | A-11008 |  |
| Goat anti-rabbit IgG Alexa Fluor 568 | Schenke et al.  Toxins. 2020; 12 :276 | Molecular Probes | A-11036 |  |
| Goat anti-mouse IgG Alexa Fluor 594 | Gayatri et al.  Cell Death Discov. 2022; 8: 277 | Molecular Probes | A-11005 |  |
| Goat anti-mouse IgG HRP-conjugated | Roof AK et al. Endocrinology. 2018; 159: 2421-34 | BioRad | 1706516 |  |
| Goat anti-rabbit IgG HRP-conjugated | Vitor AC et al. Sci Adv. 2019; 5:eaau1249 | BioRad | 1706515 |  |
| Rabbit anti-human IgG HRP-conjugates | Clement et al. Malar J. 2012; 11: 384 | Dako | P0214 |  |
| Mouse anti-human IgG1 HRP | Liu et al.  Nephrology. 2008; 13: 629-35 | Southern Biotech | 9052-05 | 4E3 |
| Mouse anti-human IgG4 HRP | Bogdanos et al.  J Hepatol. 2008; 49: 466-73. | Southern Biotech | 9200-05 | HP6025 |

- 1. **Cell lines**

| **Name** | **Citation** | **Supplier** | **Cat no.** | **Passage no.** | **Authentication test method** |
| --- | --- | --- | --- | --- | --- |
| H69 human cholangiocytes | Grubman SA et al. Am J Physiol. 1994; 266: G1060-70 | Gift from Dr. Douglas Jefferson |  | P05 to P20 | RNA sequencing for transcriptomic cell profile; deposited in GEO under GSE221746 |

- 1. **Organisms**

| **Name** | **Citation** | **Supplier** | **Strain** | **Sex** | **Age** | **Overall n number** |
| --- | --- | --- | --- | --- | --- | --- |
|  |  |  |  |  |  |  |

- 1. **Sequence based reagents**

| **Name** | **Sequence** | **Supplier** |
| --- | --- | --- |
| h*LAMA5* | **Fw** TGCTGCCTAGCGCATACTAC  **Rv** TGTAGAGGAGGCAGTTGTCG | Sigma-Aldrich |
| h*LAMB1* | **Fw** GAGTGTATGCTCGGCAGTGT  **Rv** GTAACCAGCCAAGCACCTTT | Sigma-Aldrich |
| h*LAMC1* | **Fw** AGCGCTGTCCAAAAGAATGC  **Rv** CCTGGTCAGCGTCATCTTGT | Sigma-Aldrich |
| h*CA2* | **Fw** TTACTGGACCTACCCAGGCT  **Rv** ACGGAATTTCAACACCTGCTC | Sigma-Aldrich |
| h*SLC4A2* | **Fw** GAAGACGAACTTCACCGCAC  **Rv** ACTGGCGGTGGTACTCAAAG | Sigma-Aldrich |
| h*CHP1* | **Fw** GGACGAAGAGCTCGAGGAGA  **Rv** CTGGAAATCTTCCCGGCTGA | Sigma-Aldrich |
| h*SLC9A1* | **Fw** AAGCGCTCCATCAACGAAGA  **Rv** AAACCGGTTGAGCTTGTCCT | Sigma-Aldrich |
| h*36B4* (*RPLP0*) | **Fw** TCATCAACGGGTACAAACGA  **Rv** GCCTTGACCTTTTCAGCAAG | Sigma-Aldrich |
| h*HPRT* | **Fw** CCTGGCGTCGTGATTAGTGAT  **Rv** AGACGTTCAGTCCTGTCCATAA | Sigma-Aldrich |
| shRNA *LAMA5*  TCRN0000119152 | CTCGCCTCATAGGTGTCTATT | Sigma-Aldrich  MISSION™ shRNA Library |
| shRNA *LAMB1*  TRCN0000083431 | CCCAAGGATACAGAATTTATT | Sigma-Aldrich  MISSION™ shRNA Library |
| shRNA *LAMC1*  TRCN0000119110 | CCCAAAGTTCTCAAGTCCTAT | Sigma-Aldrich  MISSION™ shRNA Library |
| shRNA SHC002  TRC1/1.5 | CAACAAGATGAAGAGCACCAA |  |

- 1. **Biological samples**

| **Description** | **Source** | **Identifier** |
| --- | --- | --- |
| IgG4-related cholangitis serum samples | Department of Gastroenterology and Hepatology, Amsterdam UMC, the Netherlands | MEC 10/007 |
| Primary sclerosing cholangitis serum samples | Department of Gastroenterology and Hepatology, Amsterdam UMC, the Netherlands | MEC 10/007 |
| Healthy volunteer serum samples | Amsterdam UMC, the Netherlands | MEC 2020/081 |
| Extrahepatic bile duct tissue from control and anti-laminin 511-E8 positive individuals with IRC | Amsterdam UMC HPB Pathology biobank | TcB2018-063 |

- 1. **Deposited data**

| **Name of repository** | **Identifier** | **Link** |
| --- | --- | --- |
| NCBI Gene Expression Omnibus (GEO) | GSE221746 |  |

- 1. **Software**

| **Software name** | **Manufacturer** | **Version** |
| --- | --- | --- |
| eLabJournal and eLabInventory, Electronic Lab Notebook (ELN) | eLabNext Eppendorf Group | 2.32.2 |
| ImageJ | Wayne Rasband, NIH | 1.50i |
| LinRegPCR | Amsterdam UMC | 2013.0 |
| GraphPad Prism | GraphPad | 9 |
| R Console | The R Foundation for Statistical Computing http://www.R-project.org | R 4.0.2 GUI 1.72 |
| RStudio | RStudio, PBC | 1.3.1093 |
| R2: Genomics Analysis and Visualization Platform  (http://r2.amc.nl http://r2platform.com) | Dr. Koster, Amsterdam UMC, Center for Experimental and Molecular Medicine (CEMM) |  |
| Leica Application Suite X (LAS X) | Leica Microsystems |  |
| MARS microplate reader software | BMG Labtech | v.4.20 compatible with CLARIOstar and CLARIOstar Plus |

- 1. **Other (*e.g*. drugs, proteins, vectors etc.)**

| Complete^TM^ Protease Inhibitor Cocktail | 11697498001 | Roche |
| --- | --- | --- |
| Pierce^TM^ BCA Protein Assay Kit | 23225 | Thermo Scientific |
| Lumi-light | 12015196001 | Roche |
| BCECF-AM | B1170 | Thermo Scientific |
| HBSS phenol red free | 10-527F | Lonza |
| Glycochenodeoxycholate | G0759 | Sigma-Aldrich |
| 22,23-^3^H-sodium glycochenodeoxycholate | - | Dr. Alan Hofmann |
| Fatty-acid free BSA | A6003 | Sigma-Aldrich |
| Digitonin | 3043 | Merck |
| 3,3′,5,5′-tetramethylbenzidine (TMB) | CL07 | Merck |
| Stop solution | N600 | Thermo Scientific |
| TRIzol | T92424 | Sigma |
| Chloroform | 2445 | Merck |
| Isopropyl alcohol | 1040 | Merck |
| Diethylpyrocarbonate (DEPC) | D5757 | Sigma |
| DNase I | M6101 | Promega |
| Random Hexamer primers | SO142 | Promega |
| Revertaid transcriptase | EP0442 | Fermentas |
| Nigericin (sodium salt) | 11437 | Cayman Chemical |
| SensoLyte^®^ Homogeneous Rh110 Caspase-3/7 Assay Kit | AS-71141 | AnaSpec |
| Raptinal | AG-CR1-2902-M005 | AdipoGen Life Sciences |
| Fluorescein isothiocyanate–dextran (FD4) | FD4-250MG | Sigma-Aldrich |
| ECMatrix-511 E8 Laminin Substrate | CC160-350UG | Sigma-Aldrich |
| Ficoll-Paque Plus  (GE Healthcare / Cytiva) | 17-1440-03 | Sigma-Aldrich |
| Ionomycin Ca-salt | I-24222 | Invitrogen |
| Phorbol-12-Myristate-13-Acetate (PMA) | sc-3576A | Santa Cruz |
| Trilogy | 920P-06 | Cell Marque |
| Citrate buffer, antigen Retriever | C9999 | Sigma-Aldrich |
| Prolong Diamond Antifade Mountant | P36965 | Thermo Scientific |
| Hoechst 33342 | 62249 | Thermo Scientific |

- 1. **Please provide the details of the corresponding methods author for the manuscript:**

| David Trampert. Amsterdam UMC, University of Amsterdam, Department of Gastroenterology and Hepatology, Tytgat Institute for Liver and Intestinal Research, Amsterdam Gastroenterology Endocrinology Metabolism (AGEM), Meibergdreef 9, Amsterdam, the Netherlands. E-mail: d.c.trampert@amsterdamumc.nl |
| --- |

**2.0 Please confirm for randomised controlled trials all versions of the clinical protocol are included in the submission. These will be published online as supplementary information.**

|  |
| --- |
