# Supplementary material for: Laminin 511-E8, an autoantigen in IgG4-related cholangitis, contributes to cholangiocyte protection
Source: JHEP Rep. 2024 Jan 23;6(4):101015. doi: 10.1016/j.jhepr.2024.101015 (PMC10959701; doi:10.1016/j.jhepr.2024.101015)
Supplement: Multimedia component 4 [file mmc4.pdf]

Research article

JHEP|Reports

Laminin 511-E8, an autoantigen in IgG4-related cholangitis, contributes to cholangiocyte protection

Authors

David C. Trampert, Remco Kersten, Dagmar Tolenaars, Aldo Jongejan, Stan F.J. van de Graaf, Ulrich Beuers

Correspondence

u.h.beuers@amsterdamumc.nl (U. Beuers).

Graphical abstract

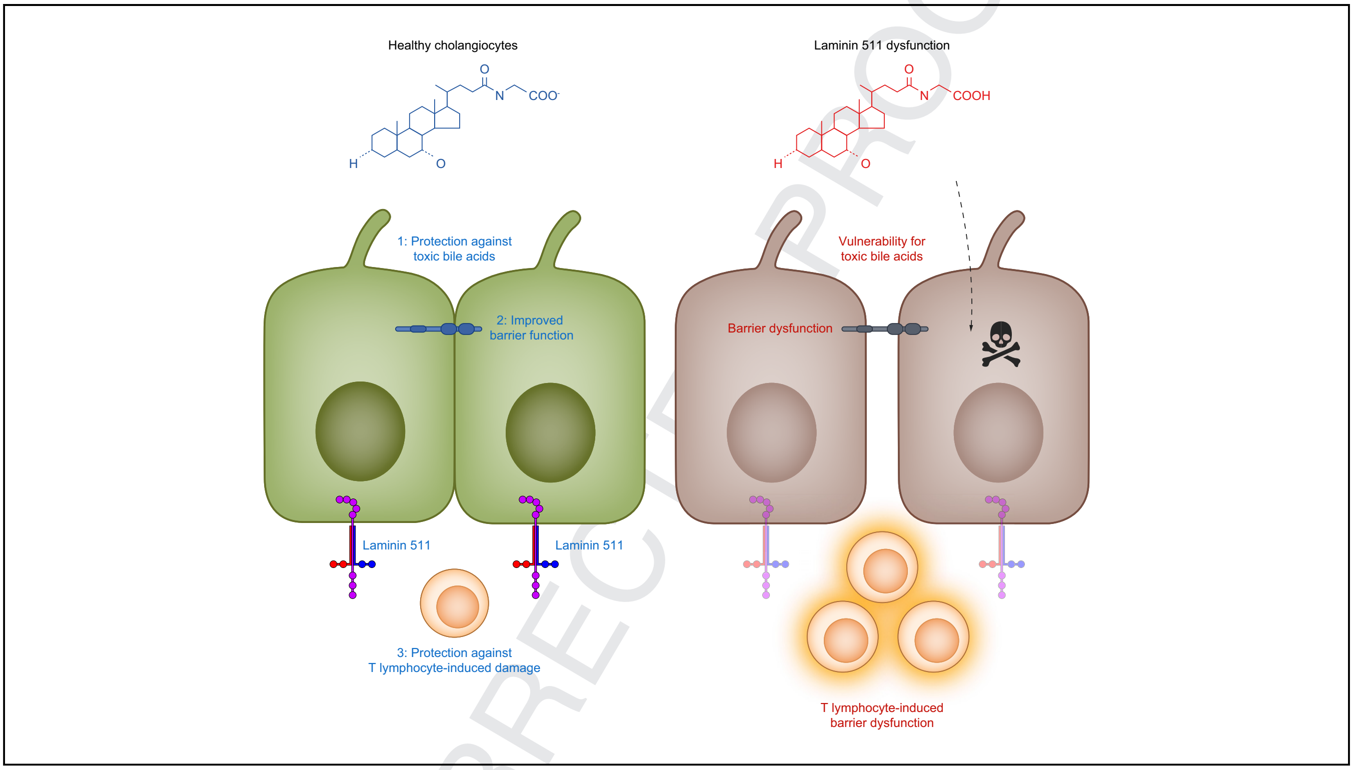

Highlights

- Laminin 511-E8 is an autoantigen in a subset of individuals with IRC.
- Laminin 511 protects cholangiocytes against toxic bile acids.
- Laminin 511 improves cholangiocellular barrier function.
- Laminin 511 protects the cholangiocyte barrier from T lymphocyte-induced damage.
- Laminin 511 and claudin 1 expression may be altered in individuals with IRC.

Impact and implications

A subset of patients with IgG4-related cholangitis (IRC) has autoantibodies against laminin 511-E8. In human cholangiocytes, laminin 511 protects against (T lymphocyte-induced) epithelial barrier dysfunction and hydrophobic bile acids. Laminin 511 and claudin 1 staining may be altered in extrahepatic bile ducts of patients with IRC who are anti-laminin 511-E8 positive. This makes it tempting to speculate that a decreased epithelial barrier function with attraction of immune cells and impaired bicarbonate secretion as a result of dysfunction of laminin 511 by autoantibody binding could potentially be a common systemic pathogenetic mechanism in a subset of patients with IgG4-RD.

https://doi.org/10.1016/j.jhepr.2024.101015

UNCORRECTED PROOF

UNCORRECTED PROOF

FLA 5.6.0 DTD ■ JHEPR101015_grabs ■ 23 February 2024 ■ 12:09 pm ■ ce

Laminin 511-E8, an autoantigen in IgG4-related cholangitis, contributes to cholangiocyte protection

David C. Trampert,[1,†] **Remco Kersten**,[1,†] Dagmar Tolenaars,[1] Aldo Jongejan,[2] Stan F.J. van de Graaf,[1] Ulrich Beuers[1,*]

[1]*Department of Gastroenterology and Hepatology, Tytgat Institute for Liver and Intestinal Research, Amsterdam Gastroenterology Endocrinology Metabolism (AGEM), Amsterdam UMC, Location AMC, University of Amsterdam, Amsterdam, The Netherlands;* [2]*Department of Epidemiology & Data Science, Bioinformatics Laboratory, Amsterdam Public Health Research Institute, Amsterdam UMC, Location AMC, University of Amsterdam, Amsterdam, The Netherlands*

JHEP Reports **2024.** https://doi.org/10.1016/j.jhepr.2024.101015

Background & Aims: IgG4-related cholangitis (IRC) is the hepatobiliary manifestation of IgG4-related disease. Anti-laminin 511-E8 autoantibodies have been identified in its pancreatic manifestation. Laminin 511-E8 promotes endothelial barrier function, lymphocyte recruitment, and cholangiocyte differentiation. Here, we investigate anti-laminin 511-E8 autoantibody presence in IRC, and mechanisms via which laminin 511 may contribute to cholangiocyte protection.
Methods: Anti-laminin 511-E8 serum autoantibody positivity was assessed by ELISA. RNA sequencing and RT-qPCR were performed on human H69 cholangiocytes treated with recombinant laminin 511-E8. H69 cholangiocytes were subjected to shRNA knockdown targeting genes encoding laminin 511 (*LAMA5*, *LAMB1*, *LAMC1*) or treated with recombinant laminin 511-E8. Cholangiocellular bile acid influx was quantified radiochemically using 22,23-^{3}H-glycochenodeoxycholic acid (GCDC). GCDC-induced apoptosis was determined by Caspase-3/7 assays. Cholangiocellular barrier function was assessed by FITC-Dextran permeability assays. Immunofluorescent staining of laminin 511 and claudin 1 was performed on extrahepatic bile duct tissue of control and anti-laminin 511-E8 positive individuals with IRC.
Results: Seven out of 52 individuals with IRC had autoantibodies against laminin 511-E8. Recombinant laminin 511-E8 led to differential expression of genes involved in secretion, barrier function, and inflammation. Knockdown of laminin 511 constituents increased toxic bile acid permeation and GCDC-induced apoptosis. Laminin 511-E8 treatment decreased toxic bile acid permeation and dose-dependently alleviated GCDC-induced apoptosis. *LAMA5* and *LAMC1* knockdown increased transepithelial permeability. Laminin 511-E8 treatment reduced transepithelial permeability and prevented T lymphocyte-induced barrier dysfunction. Laminin 511 and claudin 1 staining patterns appeared altered in anti-laminin 511-E8 positive individuals with IRC.
Conclusions: Laminin 511-E8 is an autoantigen in subsets of individuals with IRC. Laminin 511 enhances cholangiocellular barrier function and protects cholangiocytes against T lymphocyte-induced barrier dysfunction, toxic bile acid permeation and bile acid-induced apoptosis.
Impact and implications: A subset of patients with IgG4-related cholangitis (IRC) has autoantibodies against laminin 511-E8. In human cholangiocytes, laminin 511 protects against (T lymphocyte-induced) epithelial barrier dysfunction and hydrophobic bile acids. Laminin 511 and claudin 1 staining may be altered in extrahepatic bile ducts of patients with IRC who are anti-laminin 511-E8 positive. This makes it tempting to speculate that a decreased epithelial barrier function with attraction of immune cells and impaired bicarbonate secretion as a result of dysfunction of laminin 511 by autoantibody binding could potentially be a common systemic pathogenic mechanism in a subset of patients with IgG4-RD.
© 2024 The Author(s). Published by Elsevier B.V. on behalf of European Association for the Study of the Liver (EASL). This is an open access article under the CC BY license (http://creativecommons.org/licenses/by/4.0/).

Introduction

IgG4-related disease (IgG4-RD) is a systemic, lymphocyte driven, fibroinflammatory disorder characterized by elevated IgG4 serum levels and typical histopathological findings in affected organs. The most common abdominal organ manifestations in IgG4-RD are autoimmune pancreatitis (type I AIP) and IgG4-related cholangitis (IRC).[1,2] The fibroinflammatory nature of IRC leads to thickening of the bile duct wall and stenosis formation, both of which are features overlapping with hallmark signs of primary sclerosing cholangitis (PSC) and cholangiocarcinoma (CCA). Currently, no single accurate diagnostic test is available to distinguish IRC from these conditions. IRC therefore presents a major diagnostic challenge, with misdiagnosis and unjustified major surgical resections being a relatively common occurrence (Table S1).[3]

Keywords: Activated T lymphocytes; Biliary bicarbonate umbrella; Cholangiopathy; Epithelial barrier function; GCDC; Glycochenodeoxycholate; Hydrophobic bile acids; IgG4-related disease; IgG4-RD; Immune-mediated disease; Laminin.
Received 19 June 2023; received in revised form 4 January 2024; accepted 11 January 2024; available online 23 January 2024
† These authors contributed equally to this work and share first authorship.
* Corresponding author. Address: Department of Gastroenterology and Hepatology, Tytgat Institute for Liver and Intestinal Research, Amsterdam UMC, University of Amsterdam, Location AMC (C2-327), Meibergdreef 9, 1105 AZ Amsterdam, The Netherlands. Tel.: +31-20-5662422; Fax: +31-20-5669701.
E-mail address: u.h.beuers@amsterdamumc.nl (U. Beuers).

ELSEVIER

EASL The Home of Hepatology

In light of this clinical problem, understanding the pathogenesis of IRC is of great importance.

Previous studies have demonstrated the presence of affinity-maturated dominant B cell receptor clones in peripheral blood and affected tissue of individuals with IgG4-RD.[4–6] This raised the suspicion that specific autoantibodies are present in patients with IgG4-RD and led to the discovery of the autoantigens annexin A11 (ANXA11), galectin-3, laminin 511-E8, and prohibitin 1.[7–9] The pathogenicity of autoantibodies was strongly suggested after injection of patient-derived IgG1 or IgG4 autoantibodies into mice led to the development of typical organ lesions in salivary glands and pancreas which were more intense after IgG1 than IgG4 injection.[10] Simultaneous injection showed that IgG4 alleviated the more pathogenic effects of IgG1. In the same line, IgG4 autoantibodies directed against annexin A11 blocked binding of IgG1 autoantibodies to annexin A11 supporting an anti-inflammatory role of IgG4 in IgG4-RD.[7] Additional support for the pathogenicity of autoantibodies in IgG4-RD comes from the observation that individuals with IgG4-RD who are positive for multiple autoantibodies have increased disease severity[11] and that autoantibody levels decrease after successful treatment.[8] Autoantibodies could potentially contribute to the pathogenesis of IgG4-RD either by directly interfering with the endogenous function of the targeted autoantigen or by eliciting an excessive immune response after binding to the autoantigen. To address the potential direct pathogenicity of these autoantibodies, an improved understanding of the physiological role of the targeted autoantigens is pivotal.

To this end, we have previously investigated the role of the autoantigen annexin A11 in cholangiocytes,[12] which has a critical role in trafficking anoctamin 1 (ANO1), a calcium-activated chloride channel, to the apical cholangiocyte membrane. Patient serum containing autoantibodies directed against annexin A11 inhibited the membrane trafficking of ANO1, thereby potentially preventing its function at the plasma membrane of establishing a chloride gradient to facilitate biliary bicarbonate secretion. Biliary bicarbonate secretion is thought to keep millimolar levels of bile salts within the bile duct lumen in their deprotonated, membrane-impermeable and thus non-toxic state.[13,14] These findings are of interest as the autoantigen laminin 511-E8 has been shown to promote the differentiation of induced pluripotent stem cells (iPSCs) towards cholangiocytes with upregulation of secretory components such as the cystic fibrosis transmembrane conductance regulator (*CFTR*), the G protein-coupled bile acid receptor 1 (*GPBAR1*, *TGR5*), and secretin receptor (*SCTR*), which are all instrumental in adequate apical bicarbonate secretion.[15] Laminin 511 is a heterotrimeric extracellular matrix protein composed of the subunits alpha-5, beta-1, and gamma-1.[16] Anti-laminin 511 autoantibodies are directed against the cell-binding proteolytic laminin 511-E8 fragment in type 1 AIP.[8]

In addition to potential secretory defects in IRC, an impaired cholangiocellular barrier function has been proposed to play a role in IRC. Bile and brush cytology samples taken upon endoscopic retrograde cholangiopancreaticography (ERCP) from individuals with IRC demonstrated an increase in the T cell cytokines IL-4 and IL-13.[17] These cytokines negatively impacted cholangiocellular barrier function *in vitro* through differential expression of tight junction-associated claudin genes *CLDN1* and *CLDN2*. Numerous T cell populations are involved in the pathogenesis of IgG4-RD including regulatory T cells, follicular T helper 2, T peripheral helper, and cytotoxic $CD8^+$ and $CD4^+$ T cells.[2,18] Notably in endothelial cells, laminin 511 has been shown to strengthen endothelial barrier function by stabilizing vascular endothelial-cadherin and increasing the expression of tight junction proteins.[19] At the endothelial barrier, laminin 511 prevents the extravasation of leukocytes[20,21] and activation of T cells.[22]

In the present study, we investigate whether laminin 511-E8 is an autoantigen in IgG4-related cholangitis and study the role of laminin 511 in human cholangiocytes. Based on our own studies and the current literature, we hypothesize that laminin 511 protects cholangiocytes against toxic bile acids and prevents T lymphocyte-induced barrier dysfunction.

Patients and methods

Human ethics statement

The use of serum samples and peripheral blood mononuclear cells (PBMCs) was approved by the local medical ethical committee in Amsterdam (MEC 10/007 and MEC 2020/081). All participants gave written informed consent before inclusion in the study. Resection specimens of extrahepatic bile duct tissue from control and anti-laminin 511-E8 positive individuals with IRC were obtained from the Amsterdam UMC HPB Pathology biobank (TcB2018-063).

Participants

Serum samples were obtained from individuals with IRC, PSC, CCA, and healthy volunteers. Diagnosis of IRC was made according to the HISORt criteria (histology, imaging, serology, other organ involvement, response to therapy),[23] whereas diagnosis of PSC was made according to the EASL Clinical Practice Guidelines on sclerosing cholangitis.[24] Diagnosis of CCA was made based on histopathology.

ELISA

Alternating rows of 96-well ELISA plates were coated with 100 µl human recombinant laminin 511-E8 (2 µg/ml) or bovine serum albumin (2 µg/ml) in coating buffer (50 mM carbonate/bicarbonate pH 9.6 and incubated at 37 °C for 1 h.[11] Wells were washed with 50 mM Tris-buffered saline (TBS) with 0.05% Tween (TBST) pH 8.0 and subsequently blocked for 1 h with 1% bovine serum albumin (BSA)/TBS pH 8.0 at room temperature. Wells were again washed and incubated with diluted sera from patients with IRC, PSC, CCA, or healthy controls (1:20 in 1% BSA/TBS pH 8.0) for 30 min at room temperature. After washing, wells were incubated for 1 h at room temperature with 0.65 µg/ml of rabbit anti-human IgG horseradish peroxidase (HRP) secondary antibody. After washing, bound reactants were detected after a 5-min incubation with 100 µl of the chromogenic substrate 3,3′,5,5′-tetramethylbenzidine. The reaction was stopped with 100 µl of stop solution and absorbance was determined at 450 nm using the CLARIOstar (BMG LABTECH, Ortenberg, Germany). Samples were considered positive for autoantibodies against laminin 511-E8 when absorbance values were greater than the mean plus two standard deviations of the healthy control group as previously described.[9,11]

Cell cultures

H69 cholangiocytes were kindly provided by Dr Douglas Jefferson (Tufts University, Boston, MS, USA) and were cultured as previously described.[25] Composition of H69 culture medium can be found in Table S2. Culture details on LX2 cells can be found in

the Supplementary methods. *Mycoplasma* contamination tests were carried out every 3 months with negative results throughout.

RNA sequencing

H69 cholangiocytes were treated with recombinant human laminin 511-E8 0.25 μg/cm^2 for 48 h. After RNA isolation by spin column, concentration, and quality were assessed using the RNA ScreenTape. Library preparation by poly(A) mRNA capture was performed using KAPA mRNA Hyperprep. Samples were pair-end sequenced (150 bp) on the NovaSeq (Illumina, San Diego, CA, USA) with a sequencing depth of 40 million reads per sample. Raw data were submitted to NCBI Gene Expression Omnibus (GEO) and are freely available under GEO accession number GSE221746. For the RNA sequencing data analysis pipeline used, see the Supplementary methods.

Human PBMCs isolation and activation of T lymphocytes

Isolation of PBMCs, depletion of monocytes/macrophages by the adherence method, and activation of T lymphocytes was performed as previously described.[26–28] Peripheral blood was obtained from healthy volunteers and transferred to 50-ml conical tubes in an ML-2/BSL-2 facility. Ficoll-Paque Plus was added to the bottom of the 50-ml conical tube and centrifuged to generate a gradient. The PBMC layer was collected in a new tube, diluted with sterile filtered 0.2% BSA 2 mM EDTA in PBS and centrifuged. Supernatant was discarded and PBMCs were resuspended in Iscove's modified Dulbecco's medium (IMDM) supplemented with 10% fetal bovine serum and 37.5 U/ml (1%) penicillin, 37.5 μg/ml (1%) streptomycin. PBMCs were seeded in culture plates for 2 h to deplete monocytes/macrophages by adherence and T lymphocytes were stimulated using phorbol-12-myristate-13-acetate (PMA) 50 ng/ml and ionomycin 1000 ng/ml.[28] Non-adherent activated T lymphocytes were collected and centrifuged. Pelleted cells were resuspended in supplemented IMDM and counted for co-culture plating.

Fluorescein isothiocyanate–Dextran permeability assay in a Transwell co-culture system

On Day 0, 12-well Transwell inserts were coated with 10 μg/ml human recombinant laminin 511-E8 in sterile PBS or 10 μg/ml BSA control coating, and left to dry for 1 h at 37 °C. After coating, H69 cholangiocytes were seeded in the apical compartment at 100,000 cells per insert. On Day 4, PBMCs were isolated from healthy volunteers, and T lymphocytes were activated with PMA and ionomycin, after which 200,000 cells were added to the basolateral compartment. The apical compartment was refreshed with H69 culture medium and the basolateral compartment contained supplemented IMDM with or without activated T lymphocytes. Co-culture was left overnight. On Day 5, 4 kDa fluorescein isothiocyanate (FITC)-Dextran permeability assays were performed. For this, medium was refreshed with 800 μl DMEM supplemented with 10% FBS and 37.5 U/ml (1%) penicillin, 37.5 μg/ml (1%) streptomycin on the basolateral side, and 250 μl of supplemented DMEM containing 1 mg/ml 4 kDa FITC–Dextran on the apical side. At timepoint 0, 100 μl of medium was transferred to a black 96-well plate to determine potential background fluorescence. In addition, an empty unseeded Transwell insert was included to determine maximal permeability of the insert itself. At t = 60, 120, 180, and 240 min, 100 μl of basolateral medium was collected per experimental condition and transferred to the black 96-well plate. FITC–Dextran fluorescence (excitation 490 nm, emission 520 nm) was measured using the CLARIOstar apparatus.

Lentivirus generation and transduction for shRNA-mediated knockdown

Lentiviral short hairpin RNA (shRNA) constructs were purchased from the MISSION® TRC version 1 shRNA library (Sigma-Aldrich, St. Louis, MO, USA): *LAMA5* (TCRN0000119152), *LAMB1* (TRCN0000083431), *LAMC1* (TRCN0000119110) and the non-targeting shRNA control (SHC002). Lentivirus was produced as previously described in an ML-2/BSL-2 facility.[29] Transduced H69 cholangiocytes were selected with 1 μg/ml puromycin to obtain stable *LAMA5*, *LAMB1,* and *LAMC1* knockdown cholangiocytes.

Western blotting

Cells were lysed in radioimmunoprecipitation assay buffer and lysate samples were prepared in lithium dodecyl sulfate sample buffer. After running the samples on SDS-PAGE gels (3–15% Tris-acetate for LAMA5, 4–10% Tris-glycine for LAMB1 and LAMC1) in running buffer (50 mM tricine, 50 mM Tris, 0.1% SDS, and 1.3 mM sodium bisulfite at pH 8.2), samples were transferred by wet-transfer in ethanolamine/glycine transfer buffer to polyvinylidene difluoride membranes, blocked for 2 h at room temperature in 5% non-fat milk/TBST and probed overnight at 4 °C with the respective primary antibody (see CTAT methods). Immune complexes were detected with HRP-conjugated secondary antibodies and visualized using enhanced chemiluminescence detection reagent (Lumi-light, Roche Diagnostics, Rotkreuz, Switzerland) and ImageQuant LAS 4000 (GE Healthcare, Chicago, IL, USA). Protein bands were quantified using ImageJ 1.50i (Wayne Rasband, National Institutes of Health, Bethesda, MD, USA). Results were presented as fold changes of control (shRNA control; SHC002) transduced H69 cells, normalized per experiment.

RNA isolation, cDNA synthesis, and real-time quantitative PCR

For RNA isolation, cDNA synthesis, primer design, RT-qPCR and data analysis, see Supplementary methods.

Bile acid permeation assay

Bile acid permeation assays were performed as previously described.[12]

Caspase-3/7 assay

Apoptosis was determined with Caspase-3/7 assays in H69 cholangiocytes exposed to 1,500 μM glycochenodeoxycholic acid (GCDC) or 10 μM Raptinal as a positive control as described previously.[13,30]

Immunofluorescent staining and confocal microscopy

Paraffin-embedded extrahepatic bile duct tissue of control and anti-laminin 511-E8 positive individuals with IRC were sectioned at 4 μm thickness. Slides were deparaffinized, re-hydrated and unmasked in a steamed Trilogy (Cell Marque) solution. Additional antigen retrieval was done in steamed 10 mM sodium citrate (Sigma) solution. Autofluorescence was quenched using 50 mM NH_4Cl in PBS pH 7.4 at room temperature. Slides were

blocked in 5% normal goat serum in 1% BSA/TBST for 1 h. Primary antibodies were added to the tissue sections at a 1:100 dilution in 1% BSA in TBST overnight at 4 °C. Alexa Fluor 488 was used as secondary antibody for laminin 511-E8 and claudin 1, whereas Alexa Fluor 568 and 594 were used for cytokeratin 7 alongside Hoechst 33324 for nuclear staining. See CTAT methods for all antibodies used. Tissue sections were mounted with Prolong Gold antifade (Invitrogen) and 1.5 mm cover glasses. Stainings were imaged using the SP8-X DLS LightSheet confocal microscope by a researcher blinded to patient conditions. Three randomly selected fields were captured for each condition.

Statistical analysis

Data are presented as means with standard deviations. Statistical analyses were performed with GraphPad Prism 9 (GraphPad, La Jolla, CA, USA). Results of two groups were compared with a paired or unpaired *t* test where appropriate. One-way ANOVA was used when comparing multiple groups. Values of $p < 0.05$ were considered statistically significant.

Results

Autoantibodies against laminin 511-E8 are present in a subset of individuals with IRC

Seven out of 52 individuals with IRC (13.5%) were found to have autoantibodies against laminin 511-E8, but none of the disease controls with PSC (n = 13) or CCA (n = 14). One of 13 healthy volunteers had autoantibodies against laminin 511-E8 (7.7%) (Fig. 1A). IgG subtype classification showed that two individuals with IRC had autoantibodies of the IgG1 subtype, one had autoantibodies of the IgG4 subtype and one had autoantibodies of both the IgG1 and IgG4 subtype (Fig. 1B and C). There were no differences between individuals with IRC positive or negative for laminin 511-E8 autoantibodies regarding age, sex, multi-organ involvement, the occurrence of malignancies, major hepatobiliary surgeries or serum liver and inflammatory tests at presentation (Table 1). Six out of seven patients had a history of 'blue-collar work', that is, skilled trades frequently involving manual labor, and had been exposed to occupational toxins and chemicals (*e.g.* gases, fumes, mineral dusts) for >1 year (Table 2).[31,32] Four laminin 511-E8 positive individuals with IgG4-RD had manifestations in other secretory organs, namely the pancreas, salivary glands, kidneys, lungs, and colon. Autoantibodies targeting other autoantigens were found in six IRC-affected individuals and four underwent major hepatobiliary surgeries for the suspicion of CCA (Table S1). Malignancy occurred in four individuals either before or after the diagnosis of IRC. In four individuals there was a considerable delay in the diagnosis of IRC, ranging from 9 to 360 months. Owing to the diagnostic challenge of IRC and accompanying initial misdiagnosis, patients were treated in different ways, with varying rates of clinical and biochemical success (Fig. S1). Thus, in our IRC cohort no relevant occupational, clinical, or biochemical differences were observed between laminin 511-E8-positive and -negative individuals.

Table 1. Comparison of IRC patients positive and negative for anti-laminin 511-E8 autoantibodies.

	Positive	Negative	*p* value
IRC patients, n (%)	7 (13.5)	45 (86.5)	
Sex (male/female)	5/2	41/4	0.1859
Age (years)	60 (42–65)	63 (51–69)	0.3344
Multi-organ, n (%)	4 (57)	33 (82.5)	0.3763
Malignancy, n (%)	4 (57)	15 (34)	0.4016
Major HPB surgery, n (%)	4 (57)	13 (29.5)	0.2033
Bilirubin (μmol/L)	92 (29–123)	56 (33–143)	0.7346
ALP (IU/L)	445 (322–560)	354 (242–638)	0.6964
gGT (IU/L)	362 (192–887)	508 (265–816)	0.6207
AST (IU/L)	93 (51–167)	83 (69–195)	0.6058
ALT (IU/L)	148 (97–234)	117 (79–330)	0.5542
CA19.9 (kU/L)	191 (183–220)	73 (24–239)	0.6465
CRP (mg/L)	30 (23–83)	11 (5–31)	0.9766
ESR (mm/h)	69 (47–88)	44 (20–71)	0.2135

Levels of significance: $p < 0.05$ was seen as significant. For categorical data (sex, multi-organ, malignancy, major HPB surgery) Fisher's exact test was performed. For numerical data (age, bilirubin, ALP, gGT, AST, ALT, CA19.9, CRP, ESR) an unpaired *t* test was performed.
ALP, alkaline phosphatase; ALT, alanine aminotransferase; AST, aspartate aminotransferase; CA19.9, cancer antigen 19.9; CRP, c-reactive protein; ESR, erythrocyte sedimentation rate; gGT, gamma-glutamyl transferase; HPB, hepatopancreatobiliary; IRC, IgG4-related cholangitis.

Laminin 511 constituents are highly expressed in models of human cholangiocytes

As cholangiocytes are the main cell type affected in IRC, laminin 511 expression was assessed in human control extrahepatic bile duct tissue by immunofluorescence (Fig. 2A). A sharp peribiliary staining pattern was observed. To differentiate the cellular origin of peribiliary laminin staining in cholangiocytes *vs.* periductal fibroblasts, gene constituent expression of laminin was studied in human cholangiocytes and compared with periductal fibroblasts that also could contribute to laminin 511 production. The laminin 511 gene constituent expression appeared highest in the

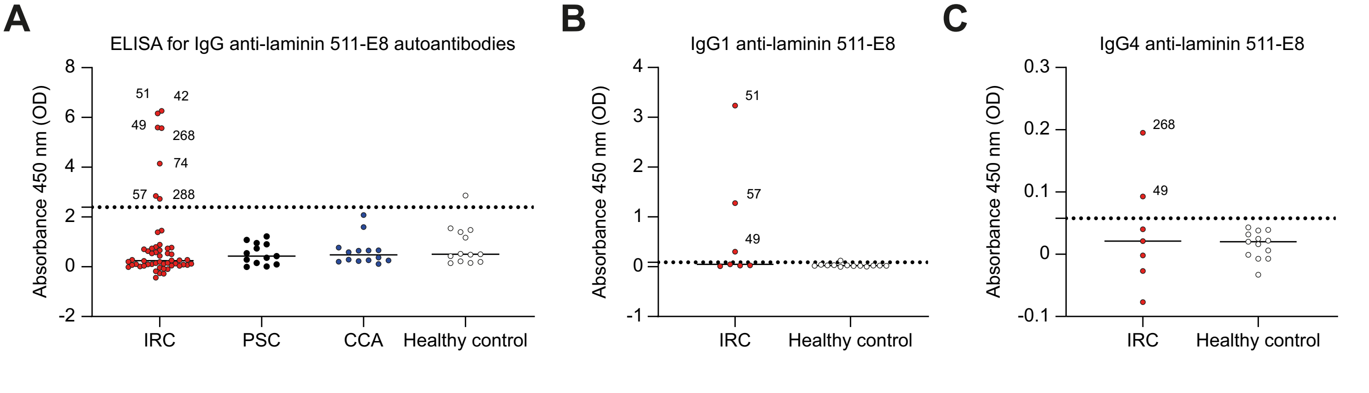

Fig. 1. Laminin 511-E8 is an autoantigen in a subset of patients with IRC. (A) Anti-laminin 511-E8 IgG in patients with IRC (7/52), PSC (0/13), CCA (0/14) and healthy controls (1/13). Anti-laminin 511-E8 IgG1 (B) and IgG4 (C) subtype classification in positive patients with IRC. Patients are presented as individual data points and positive patients are annotated. CCA, cholangiocarcinoma; IRC, IgG4-related cholangitis; PSC, primary sclerosing cholangitis.

Table 2. Characteristics of individuals with IRC positive for anti-laminin 511-E8 autoantibodies.

	#49	#42	#51	#74	#268	#288	#57
Age at onset (years)	60	52	63	42	74	65	34
Time to diagnosis (months)	3	4	9	2	93	10	360
Sex	M	M	M	F	M	M	F
Profession	Driver petrochemical company	Truck driver	Military service, limousine driver	Beautician, car sales	–	Construction worker	Cleaner
Organ involvement	IRC<br>AIP<br>Salivary glands	IRC<br>AIP<br>Kidney	IRC	IRC	IRC	IRC<br>AIP	IRC<br>AIP<br>Lung, colon
Autoantibody positivity	Laminin 511-E8	Laminin 511-E8<br>Annexin A11<br>Prohibitin 1	Laminin 511-E8<br>Prohibitin 1	Laminin 511-E8<br>Prohibitin 1	Laminin 511-E8<br>Galectin-3<br>Prohibitin 1	Laminin 511-E8<br>Prohibitin 1	Laminin 511-E8<br>Prohibitin 1<br>Annexin A11
Surgery	No	No	Hemihepatectomy	PPPD<br>Hemihepatectomy	Bile duct resection	Bile duct resection	No
Malignancy	No	No	CRC 2 years after IgG4-RD onset	CCA at diagnosis, 2× recurrence after IRC diagnosis	No	Sigmoid carcinoma 6 years prior to IgG4-RD onset	CCA 27 years after first manifestation
Therapy	ERCP with stent placement<br>Prednisolone	Spontaneous improvement	UDCA	Prednisolone<br>Azathioprine/6-Thioguanine<br>UDCA	No therapy	ERCP with stent placement<br>Prednisolone<br>6-Thioguanine<br>UDCA	Stent placement<br>UDCA<br>Prednisolone

AIP, type 1 autoimmune pancreatitis; CCA, cholangiocarcinoma; CRC, colorectal carcinoma; ERCP, endoscopic retrograde cholangiopancreaticography; IRC, IgG4-related cholangitis; PPPD, pylorus-preserving pancreatoduodenectomy; PSC, primary sclerosing cholangitis; UDCA, ursodeoxycholic acid.

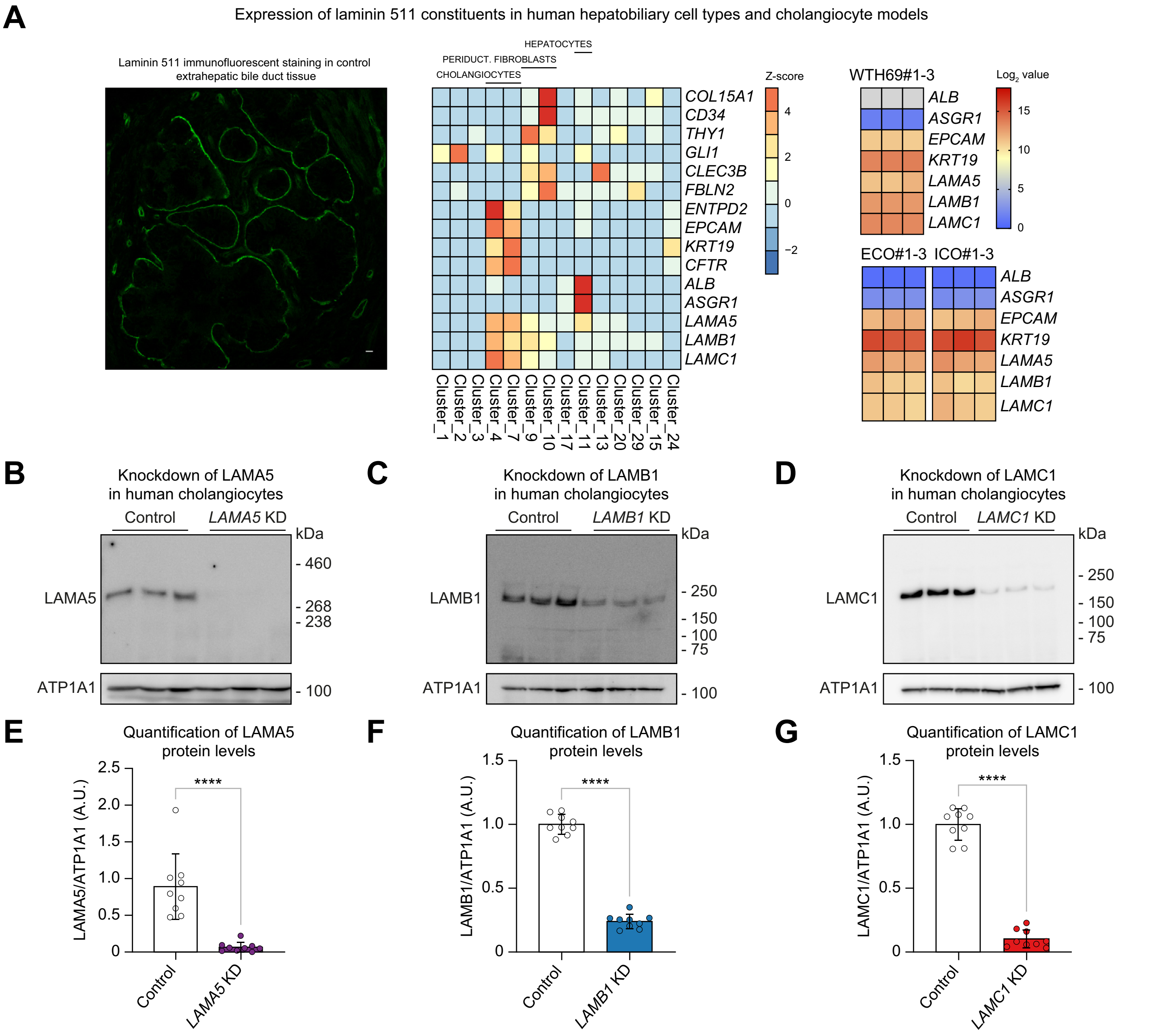

Fig. 2. Laminin 511 constituents are expressed in various human cholangiocyte models. (A) Laminin 511 immunofluorescent staining in human hepatobiliary control tissue discloses a sharp peribiliary staining pattern (scale bar = 10 μm). Left heatmap represents laminin 511 gene constituent expression in cholangiocyte, hepatocyte, and periductal fibroblast clusters from human liver. Right heatmaps demonstrate expression levels of laminin 511 constituents in two different human cholangioid models and H69 cholangiocytes. Establishment of (B) LAMA5 (400 kDa), (C) LAMB1 (198 kDa), and (D) LAMC1 (178 kDa) shRNA knockdown H69 cholangiocytes. Quantification of (E) LAMA5, (F) LAMB1, and (G) LAMC1 protein levels normalized by ATP1A1 (112 kDa), (nine cell samples from n = 3 independent experiments). Data are represented as mean with standard deviation. Levels of significance: (E–G) ****p <0.0001; unpaired t test. A.U., arbitrary units; Control, shRNA control; ECO/ICO, extra-/intrahepatic cholangiocyte organoids; KD, knockdown; *LAMA5*, laminin alpha 5 chain; *LAMB1*, laminin beta 1 chain; *LAMC1*, laminin gamma 1 chain.

cholangiocyte population (Fig. 2A). Additionally, on a protein level, LAMA5 expression was negligibly low in activated myofibroblasts compared with cholangiocytes (Fig. S2). To further determine the cholangiocellular expression of *LAMA5*, *LAMB1*, and *LAMC1*, two types of human cholangiocyte organoids and human H69 cholangiocytes were assessed. In all datasets, a cholangiocyte phenotype was demonstrated by high expression of cholangiocellular markers *EPCAM* (epithelial cell adhesion molecule) and *KRT19* (cytokeratin 19), whereas expression of the hepatocyte markers *ALB* (albumin) and *ASGR1* (asialoglycoprotein receptor 1) was low. The three genetic laminin 511 constituents *LAMA5, LAMB1, LAMC1* showed high expression across all types of cholangiocyte organoids and human H69 cholangiocytes (Fig. 2A). Thus, laminin is highly expressed in human cholangiocytes, but not periductal fibroblasts.

To further investigate the physiological role of laminin 511 in cholangiocytes, stable knockdown cell lines for *LAMA5*, *LAMB1*, and *LAMC1* were generated in human H69 cholangiocytes by lentiviral shRNA transduction. Expression of laminin 511

constituents and their respective knockdown were demonstrated on the protein level by Western blot; *LAMA5* (83% knockdown), *LAMB1* (76% knockdown), and *LAMC1* (90% knockdown) (Fig. 2B–G) and on mRNA level by RT-qPCR (Fig. S3).

Recombinant laminin 511-E8 leads to transcriptional changes in cholangiocytes relating to barrier function and inflammation

To examine the endogenous roles of laminin 511 in human cholangiocytes, RNA sequencing was performed after treatment of human H69 cholangiocytes with recombinant laminin 511-E8. Initial multidimensional scaling (MDS) showed that recombinant laminin 511-E8 treatment clustered the samples together, whereas more spread was observed in the untreated cholangiocytes (Fig. 3A). Gene set enrichment analysis showed that processes related to extracellular matrix, cell polarity, barrier function, and inflammation demonstrated enrichment after recombinant laminin 511-E8 treatment (Fig. 3B–D). Only a limited number of secretory genes were differentially expressed (Fig. 3E). To further investigate a potential relation between laminin 511 and secretion, H69 cholangiocytes were treated with a higher dose (2 μg/cm^2) of recombinant laminin 511-E8, and gene expression was studied by RT-qPCR. Increased expression was found in the secretory genes *CA2* and *SLC4A2*, while a decrease in *CHP1*, an essential co-factor of *SLC9A1*, was seen (Fig. 3F). Thus, altered gene expression after recombinant laminin 511-E8 treatment implicated roles for laminin 511 in cholangiocellular barrier function, inflammation, and secretory processes.

Knockdown of laminin 511 constituents increases toxic bile acid permeation and vulnerability to bile acid-induced apoptosis

As secretory proteins are involved in cholangiocellular protection against toxic hydrophobic bile acids, we investigated whether knockdown of the laminin 511 constituents would render cholangiocytes vulnerable to toxic bile acids.

Bile acid permeation assays demonstrated that *LAMB1* and *LAMC1* knockdown cholangiocytes showed an increased GCDC permeation at 4, 16, and 64 min, whereas *LAMA5* knockdown cholangiocytes only showed an increased toxic bile acid permeation at 64 min (Fig. 4A–F). To gain further insight into a potentially altered pH homeostasis induced by the knockdown of laminin 511 constituents, intracellular pH (pHi) measurements were performed. These assays indicated that *LAMA5*, *LAMB1,* and *LAMC1* knockdown cholangiocytes had a lower pHi compared to control H69 cholangiocytes (Fig. S4, Table S3). Baseline intracellular pH tended to decrease in *LAMA5* knockdown cholangiocytes and decreased in *LAMB1* and *LAMC1* cholangiocytes.

The increased permeation of hydrophobic bile acids is expected to result in an increased rate of apoptosis. Caspase-3/7 assays were performed in *LAMA5, LAMB1,* and *LAMC1* knockdown cholangiocytes after exposure to GCDC and Raptinal as a positive control for apoptosis induction. Increased apoptosis was seen in the *LAMB1* and *LAMC1* knockdown cholangiocytes, but not in the *LAMA5* knockdown cholangiocytes (Fig. 4G–I). The *LAMA5* knockdown cholangiocytes also showed the smallest alterations in bile acid permeation and baseline pHi. Thus, knockdown of laminin 511 constituents renders cholangiocytes vulnerable to toxic bile acids.

Recombinant laminin 511-E8 lowers toxic bile acid permeation and dose-dependently protects against bile acid-induced apoptosis

As a parallel approach to study the role of laminin 511 in human cholangiocytes, cholangiocyte susceptibility to bile acid toxicity was assessed after recombinant laminin 511-E8 treatment.

Recombinant laminin 511-E8 treatment reduced GCDC permeation into H69 cholangiocytes at 1, 4, 16, and 64 min (Fig. 5A and B). Recombinant laminin 511-E8 lowered baseline pHi (Fig. S5, Table S3). Furthermore, recombinant laminin 511-E8 dose-dependently alleviated GCDC-induced apoptosis (Fig. 5C). Thus, recombinant laminin 511-E8 treatment protects cholangiocytes against toxic bile acid permeation and bile acid-induced apoptosis.

Knockdown of laminin 511 constituents decreases barrier function in human cholangiocytes

To assess whether laminin 511 is involved in cholangiocellular barrier formation, FITC–Dextran permeability assays were performed. *LAMA5* and *LAMC1* KD led to an increase in FITC–Dextran permeability at all time points, whereas *LAMB1* KD only demonstrated a trend towards increased FITC–Dextran permeation (Fig. 6A and B). These data indicate that laminin 511 could play a role in cholangiocellular barrier formation.

Recombinant laminin 511-E8 enhances barrier function of human cholangiocytes and protects from T lymphocyte-induced barrier dysfunction

As a parallel approach, the effect of recombinant laminin 511-E8 on barrier function was assessed. Recombinant laminin 511-E8 significantly reduced FITC–Dextran permeation (Fig. 7A and B).

To further investigate the role of laminin 511-E8 in the setting of inflammation, H69 cholangiocytes were co-cultured with activated T lymphocytes (Fig. 7C). Co-culture of H69 cholangiocytes with activated T lymphocytes resulted in an increased FITC-Dextran permeability (Fig. 7D, gray *vs.* red). Strikingly, when the H69 monolayer was in basolateral contact with recombinant laminin 511-E8, cholangiocytes were protected from the T lymphocyte-induced barrier dysfunction (Fig. 7D, red *vs.* blue). Notably, the co-culture condition with laminin 511-E8 coating maintained the barrier integrity at the same level as the single H69 cholangiocyte culture condition with laminin 511-E8 coating (Fig. 7D, blue and green). These findings indicate that laminin 511-E8 improves the cholangiocellular barrier function and prevents T lymphocyte-induced barrier dysfunction.

Laminin 511 and claudin 1 staining may be altered in extrahepatic bile ducts of anti-laminin 511-E8 positive individuals with IRC

To investigate the expression and localization of laminin 511 in individuals with IgG4-related cholangitis, laminin 511 expression was assessed by immunofluorescence in extrahepatic bile duct tissue from available resection specimens of controls and patients who were anti-laminin 511-E8 positive. In healthy control tissue, all bile ducts showed a clear basolateral laminin 511 staining (Fig. 8, top panels). In one patient with IRC who was anti-laminin 511-E8 positive (#268), a similar tracing was seen but laminin 511 staining appeared fainter. In the other patient with IRC who was anti-laminin 511-E8 positive (#57) an altered staining pattern was observed with a clear loss of basolateral laminin 511 localization.

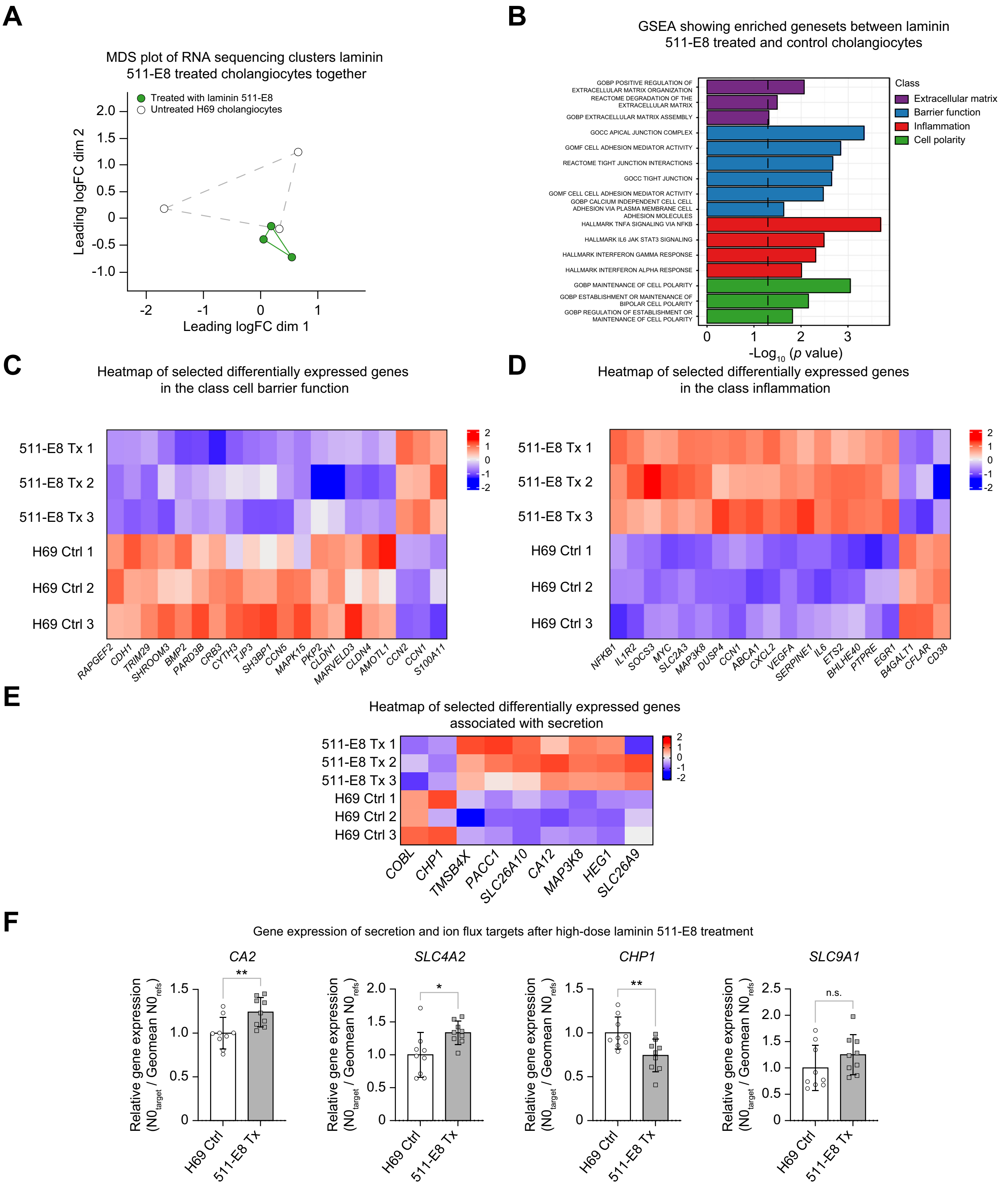

Fig. 3. Recombinant laminin 511-E8 treatment transcriptionally alters genes involved in cholangiocellular barrier function, inflammation, and secretion. (A) MDS plot of recombinant laminin 511-E8 (0.25 μg/cm^2) treated and untreated control cholangiocytes. (B) GSEA displaying enriched gene sets from MSigDB. Heatmaps of (C) differentially expressed genes relating to cell barrier function, (D) inflammation, and (E) secretion. (F) RT-qPCR target genes related to secretion after high-dose (2 μg/cm^2) recombinant laminin 511-E8. Heatmaps show Log2 transformed z-scored expression values. Levels of significance: (B) -log10 (*p* value) >1.3 was seen as significant. (F) ns = not significant, $^*p$ <0.05, $^{**}p$ <0.01; unpaired *t* test. BP, biological process; CC, cellular component; Ctrl, control; FC, fold change; GO, gene ontology; GSEA, gene set enrichment analysis; MDS, multidimensional scaling; MF, molecular function; MSigDB, molecular signatures database; N0, starting concentration.

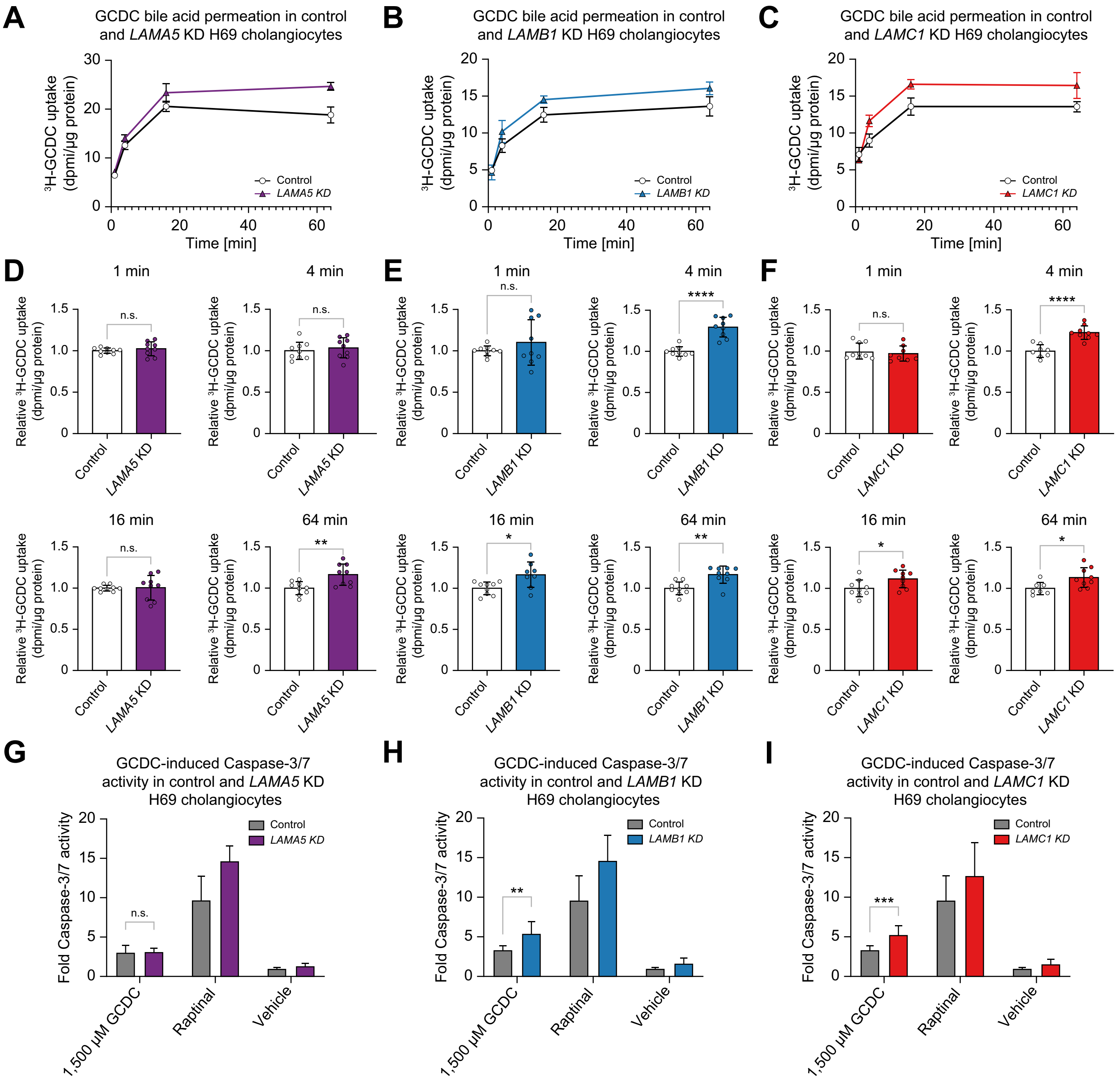

Fig. 4. Knockdown of laminin 511 constituents increases toxic bile acid permeation and vulnerability to bile acid-induced apoptosis. ^{3}H-GCDC permeation assay in control, (A) *LAMA5*, (B) *LAMB1*, and (C) *LAMC1* knockdown cholangiocytes (representative experiment of n = 3). Relative quantification of ^{3}H-GCDC permeation per timepoint in control, (D) *LAMA5*, (E) *LAMB1*, and (F) *LAMC1* knockdown cholangiocytes (seven to nine samples from n = 3 independent experiments). GCDC-induced Caspase-3/7 activity in control (G) *LAMA5*, (H) *LAMB1*, and (I) *LAMC1* knockdown cholangiocytes (nine samples from n = 3 independent experiments). Data are represented as mean with standard deviation. Levels of significance: (D–F) ns = not significant, *p <0.05, **p <0.01, ****p <0.0001; unpaired t-test. (G–I) ns = not significant, **p <0.01, ***p <0.001; unpaired t test. Control, shRNA control; DPMI, disintegrations per minute; GCDC, glycochenodeoxycholic acid; KD, knockdown; *LAMA5*, laminin alpha 5 chain; *LAMB1*, laminin beta 1 chain; *LAMC1*, laminin gamma 1 chain.

Subsequently, extrahepatic bile duct tissue was stained for the tight junction protein claudin 1. Compared with control tissue, one patient with IRC who was anti-laminin 511-E8 positive (#268) showed a fainter and disrupted claudin 1 staining (Fig. 8, bottom panels). In the other patient with IRC who was anti-laminin 511-E8 positive (#57) a clear claudin 1 staining was observed. However, the localization of claudin 1 appeared altered compared with control tissue (as indicated by a higher degree of cytokeratin 7/claudin 1 overlap). All imaged fields can be found in Fig. S6.

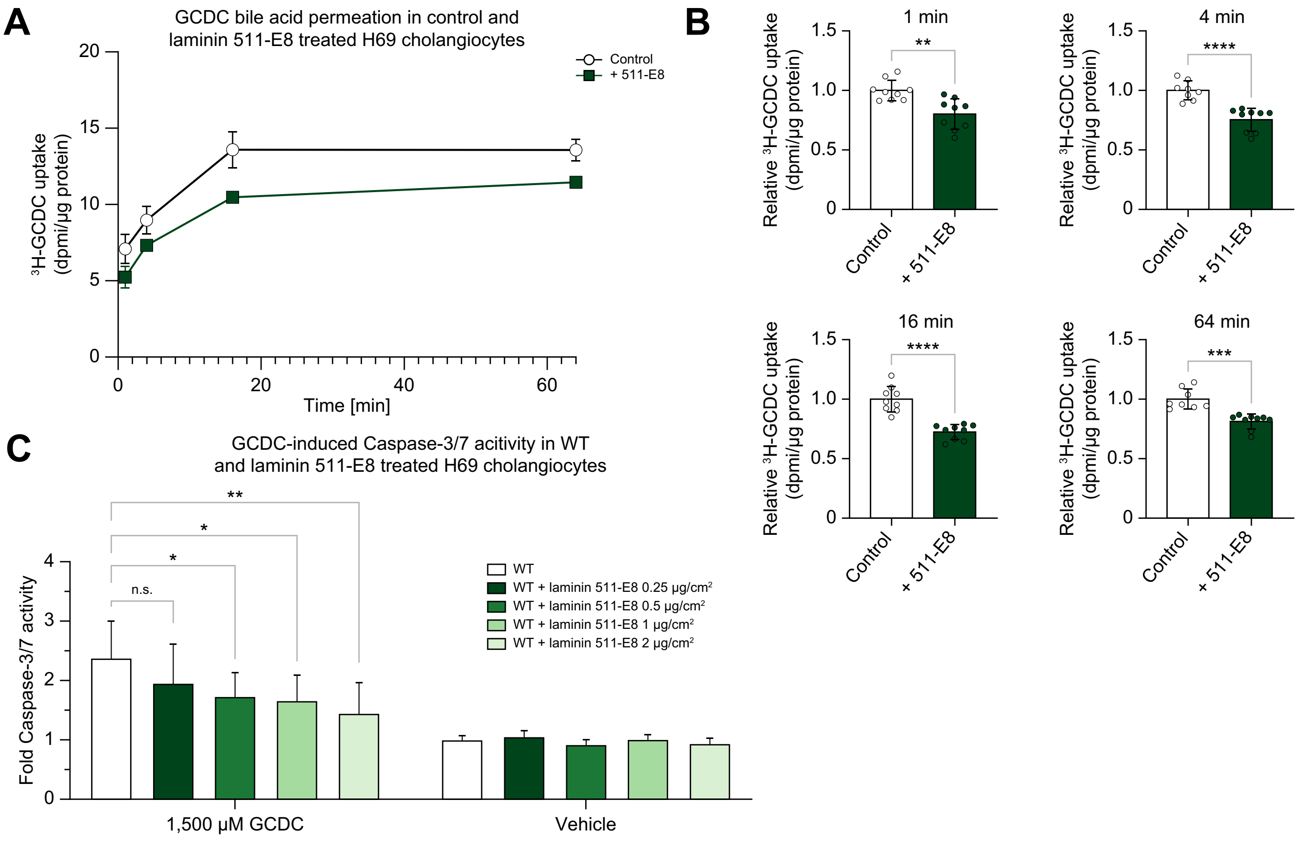

Fig. 5. Recombinant laminin 511-E8 lowers toxic bile acid permeation and dose-dependently alleviates bile acid-induced apoptosis. (A) ^{3}H-GCDC permeation assay in control and recombinant laminin 511-E8 treated H69 cholangiocytes (representative experiment of n = 3). Relative quantification of ^{3}H-GCDC permeation at (B) 1 min, 4 min, 16 min, 64 min in control and recombinant laminin 511-E8 treated H69 cholangiocytes (eight to nine samples from n = 3 independent experiments). (C) GCDC-induced Caspase-3/7 activity in WT and recombinant laminin 511-E8 treated H69 cholangiocytes (nine samples from n = 3 independent experiments). Data are represented as mean with standard deviation. Levels of significance: (B) ***p* <0.01, ****p* <0.001, *****p* <0.0001; unpaired *t* test. (C) ns = not significant, **p* <0.05, ***p* <0.01; one-way ANOVA. Control, shRNA control; DPMI, disintegrations per minute; GCDC, glycochenodeoxycholic acid; KD, knockdown; WT, wild type.

Discussion

The present study is the first to establish that the heterotrimeric extracellular matrix protein laminin 511-E8 is an autoantigen in a subset of patients with IRC. In addition, the present study unravels physiological roles of laminin 511 in human cholangiocytes, the target cells of the immune attack in IRC. Our data indicate that laminin 511 stabilizes cholangiocellular barrier function and protects human cholangiocytes against T lymphocyte-induced barrier dysfunction, toxic bile acid permeation and bile acid-induced apoptosis.

Autoantibodies against laminin 511-E8 were detected in 13.5% of patients with IRC and not in patients with PSC or CCA. Four out of seven patients positive for laminin 511-E8 autoantibodies had a malignancy. This is different from the first report of autoantibodies against laminin 511-E8 in type I AIP where none of the affected individuals with anti-laminin 511-E8 autoantibodies had a malignancy.[8] The percentage of individuals positive for anti-laminin 511-E8 autoantibodies had been higher in the cohort with type I AIP (51%) than in our IRC cohort (13.5%). This difference could potentially be explained by differences in organ manifestations (pancreas *vs.* bile ducts), the genetic background (Japanese *vs.* Caucasian) and/or environmental risk factors between the two cohorts, but remains unclear at present.

Our experimental data support a protective role of laminin 511 for human cholangiocytes. RNA sequencing implicated laminin 511 to be involved in processes of (i) secretion, (ii) barrier function, and (iii) inflammation. In our RNA sequencing dataset, we were not able to identify driving genes because of the small fold changes after recombinant laminin 511-E8 treatment at 0.25 μg/cm^2. An explanation could be the degree of variance observed in the MDS plot for untreated H69 cholangiocytes, whereas treatment with laminin 511-E8 clustered the cholangiocytes more closely together. Additionally, in previous studies, higher concentrations of recombinant laminin 511-E8 led to differential expression of secretory genes.[15,33] Indeed, treatment with 2 μg/cm^2 also led to the upregulation of several genes related to secretion in our study (*CA2*, *SLC4A2*). These proteins have been proposed as essential components of the secretory machinery in cholangiocytes.[34] In turn, these secretory pathways have been hypothesized to create an apical alkaline microenvironment – the biliary bicarbonate umbrella – that protects cholangiocytes against hydrophobic bile acids.[13,14] Our data in laminin 511 constituent KD or cell lines demonstrating increased bile acid permeation and increased susceptibility to GCDC-induced apoptosis support such a mechanism. In parallel, recombinant laminin 511-E8 treatment lowered toxic bile acid

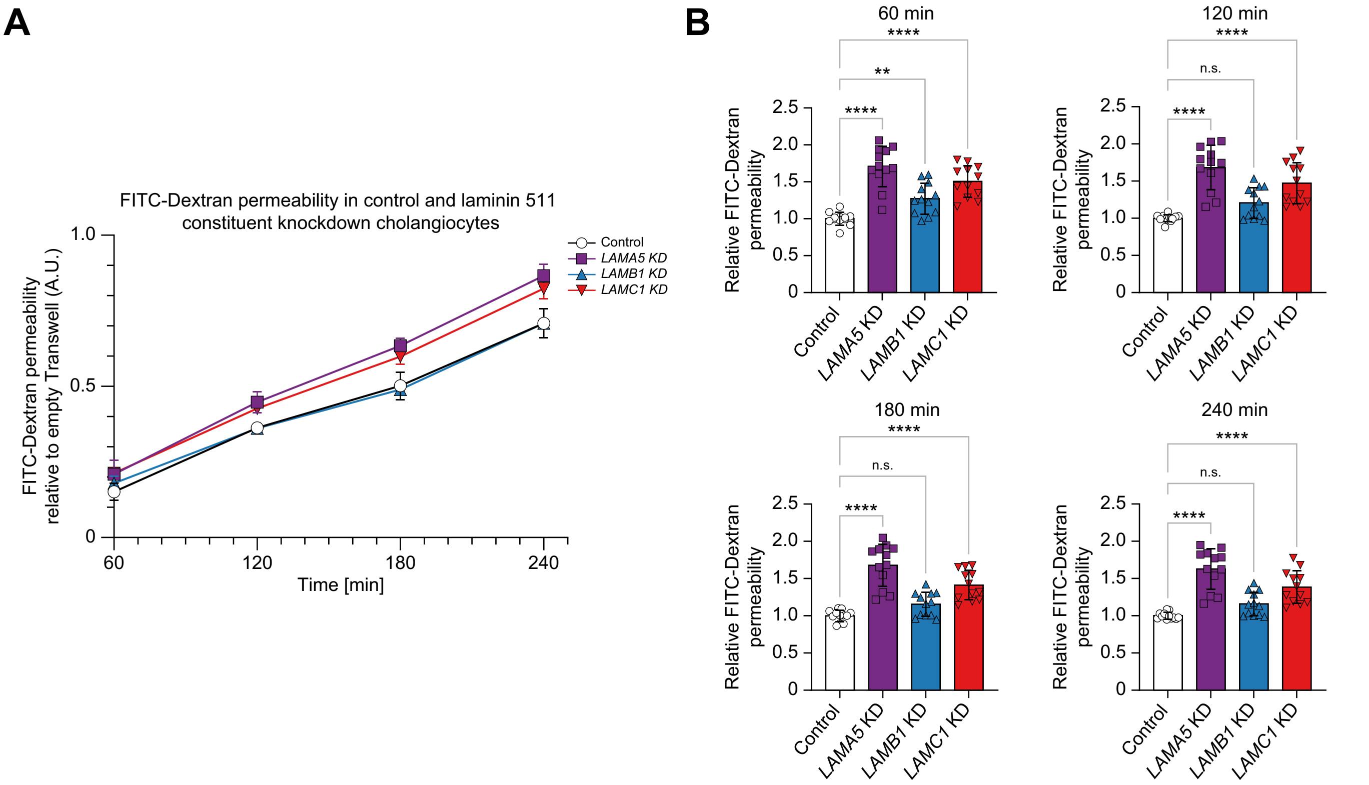

Fig. 6. Knockdown of laminin 511 constituents decreases barrier function in human cholangiocytes. (A) Kinetics of FITC–Dextran permeability assay in *LAMA5*, *LAMB1,* and *LAMC1* knockdown compared with control cholangiocytes (representative experiment of n = 4). Relative quantification of FITC–Dextran permeability assay at (B) 60 min, 120 min, 180 min, and 240 min in *LAMA5*, *LAMB1,* and *LAMC1* knockdown compared with control cholangiocytes (12 samples from n = 4 independent experiments). Data are represented as mean with standard deviation. Levels of significance: (B) ns = not significant, $^{**}p$ <0.01, $^{****}p$ <0.0001; one-way ANOVA. Control, shRNA control; FITC, fluorescein isothiocyanate; KD, knockdown; *LAMB1*, laminin beta 1 chain; *LAMC1*, laminin gamma 1 chain.

permeation and dose-dependently alleviated GCDC-induced apoptosis. Still, in the present study, the decrease in intracellular pH in both laminin 511 constituent KD cell lines and in recombinant 511-E8 treated cholangiocytes is incompletely understood. This observation can potentially be explained by the fact that (i) knockdown of one laminin 511 constituent also leads to knockdown of other laminin family members[34] and (ii) compensatory mechanisms could be at play in knockdown cholangiocytes as this is a stable and permanent strategy.[35,36]

Our data demonstrating a protective role of laminin 511 in cholangiocellular barrier function align with a previous report showing an altered expression of barrier proteins in IRC-derived cholangiocytes.[17] This study reported a downregulation of the tight-junction protein claudin 1 and an upregulation of the paracellular pore protein claudin 2. Notably, we observed an altered claudin 1 staining in extrahepatic bile ducts of two patients positive for anti-laminin 511-E8 autoantibodies (Fig. 8). However, definitive conclusions cannot be drawn regarding impaired laminin 511 and claudin 1 expression in IRC, given the limited number of anti-laminin 511-E8-positive individuals with IRC after liver resection available for assessment in our study. In the above-mentioned report,[17] the downregulation of claudin 1 and upregulation of claudin 2 were related to inflammation and were regulated by IL-4 and IL-13. These cytokines are derived from T cell populations known to be involved in the pathogenesis of IRC.[37] Notably, in endothelial cells, laminin 511 has been shown to prevent the extravasation of leukocytes and activation of T helper 17 (Th17) cells.[19,20,22,38–40] These reports fit with our RNA sequencing data suggesting a role for laminin 511 in regulating both cholangiocellular barrier function and inflammation. Indeed, our functional experiments demonstrated that laminin 511 constituent knockdown impaired cholangiocellular barrier function whereas recombinant laminin 511-E8 treatment improved cholangiocellular barrier function and prevented T lymphocyte-induced cholangiocellular barrier dysfunction.

It appears possible that autoantibodies directed against laminin 511-E8 could block its protective cholangiocellular functions – as those in other secretory organs affected by IgG4-RD.[1,2] Anti-laminin 511-E8 autoantibodies could potentially contribute to the pathogenesis of IRC by impairing cholangiocellular secretion and barrier function, thereby enhancing the risk of both, T lymphocyte- and bile acid-induced damage. Notably, four out of seven IRC patients with autoantibodies against laminin 511-E8 had IgG4-RD manifestations in other secretory organs that are associated with an impaired barrier function and defective bicarbonate secretion, being the pancreas,[41,42] salivary glands,[43,44] kidney,[45] and colon. This makes it tempting to speculate that a decreased epithelial barrier function with attraction of immune cells and impaired bicarbonate secretion because of dysfunction of laminin 511 by autoantibody binding could potentially be a common systemic pathogenic mechanism in a subset of IgG4-RD patients.

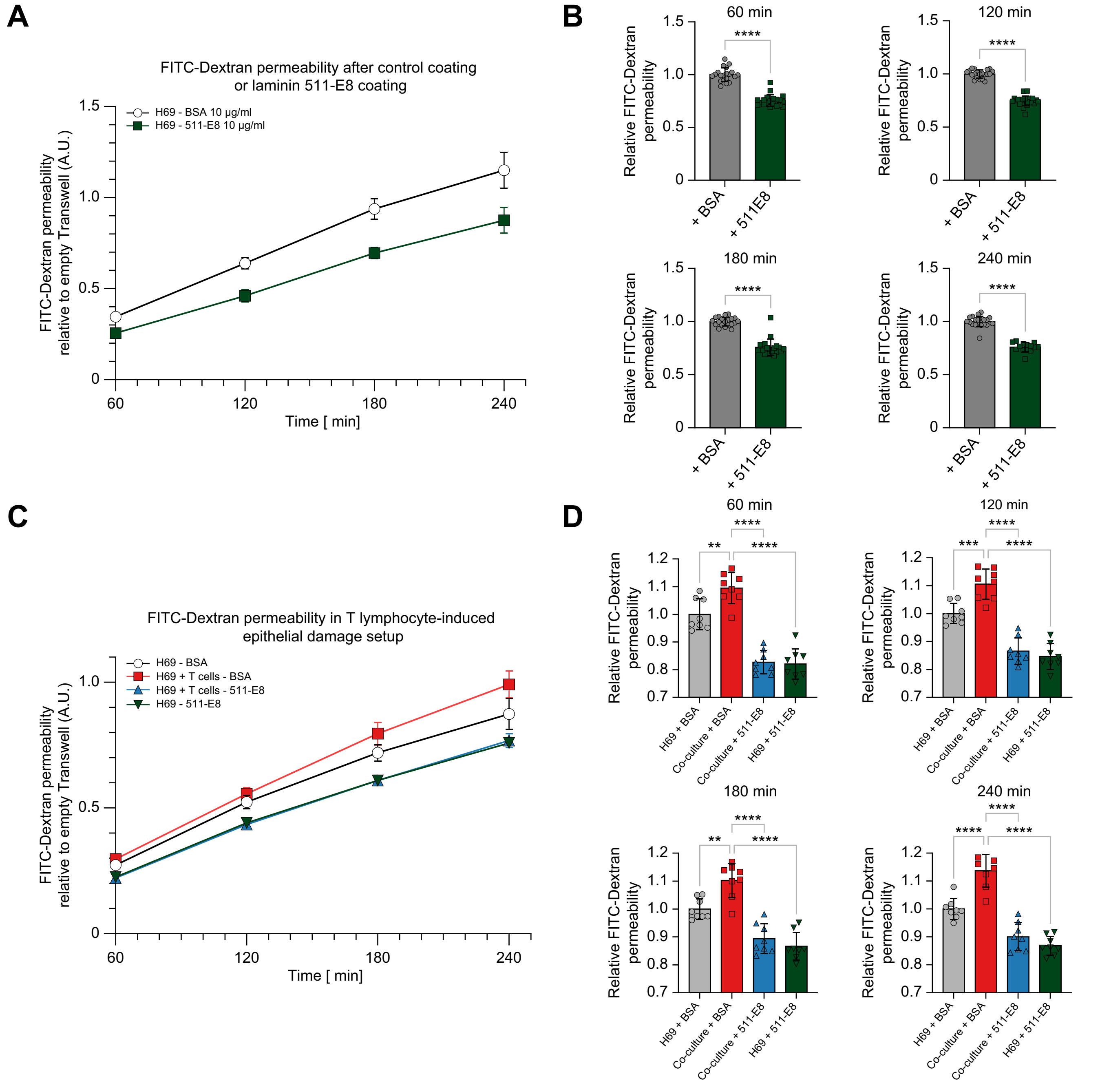

Fig. 7. Recombinant laminin 511-E8 enhances cholangiocellular barrier function and protects against T lymphocyte-induced barrier dysfunction. (A) Kinetics of FITC–Dextran permeability assay in cholangiocytes treated with recombinant laminin 511-E8 compared with BSA control coating (representative experiment of n = 4). Relative quantification of FITC–Dextran permeability assay at (B) 60 min, 120 min, 180 min, and 240 min in cholangiocytes treated with recombinant laminin 511-E8 compared with BSA control coating (20 samples from n = 4 independent experiments). (C) Kinetics of FITC-Dextran permeability assay in co-culture set-up of activated T lymphocytes with H69 cholangiocytes (representative experiment of n=3). Relative quantification of FITC–Dextran permeability assay at (D) 60 min, 120 min, 180 min, and 240 min in co-culture set-up (eight samples from n = 3 independent experiments). Data are represented as mean with standard deviation. Levels of significance: (B) ****p = <0.0001; unpaired t test. (D) **p <0.01, ***p <0.001, ****p <0.0001; one-way ANOVA. BSA, bovine serum albumin; FITC, fluorescein isothiocyanate.

In conclusion, our study demonstrates that laminin 511-E8 is an autoantigen in a subset of individuals with IRC. Laminin 511 is located in the extracellular matrix of human cholangiocytes where it promotes cholangiocellular barrier function, prevents T lymphocyte-induced cholangiocellular barrier dysfunction, and protects human cholangiocytes against permeation of toxic bile acids and bile acid-induced apoptosis. Future studies will have to assess whether anti-laminin 511 autoantibodies directly contribute to the pathogenesis of IRC in a subset of affected individuals.

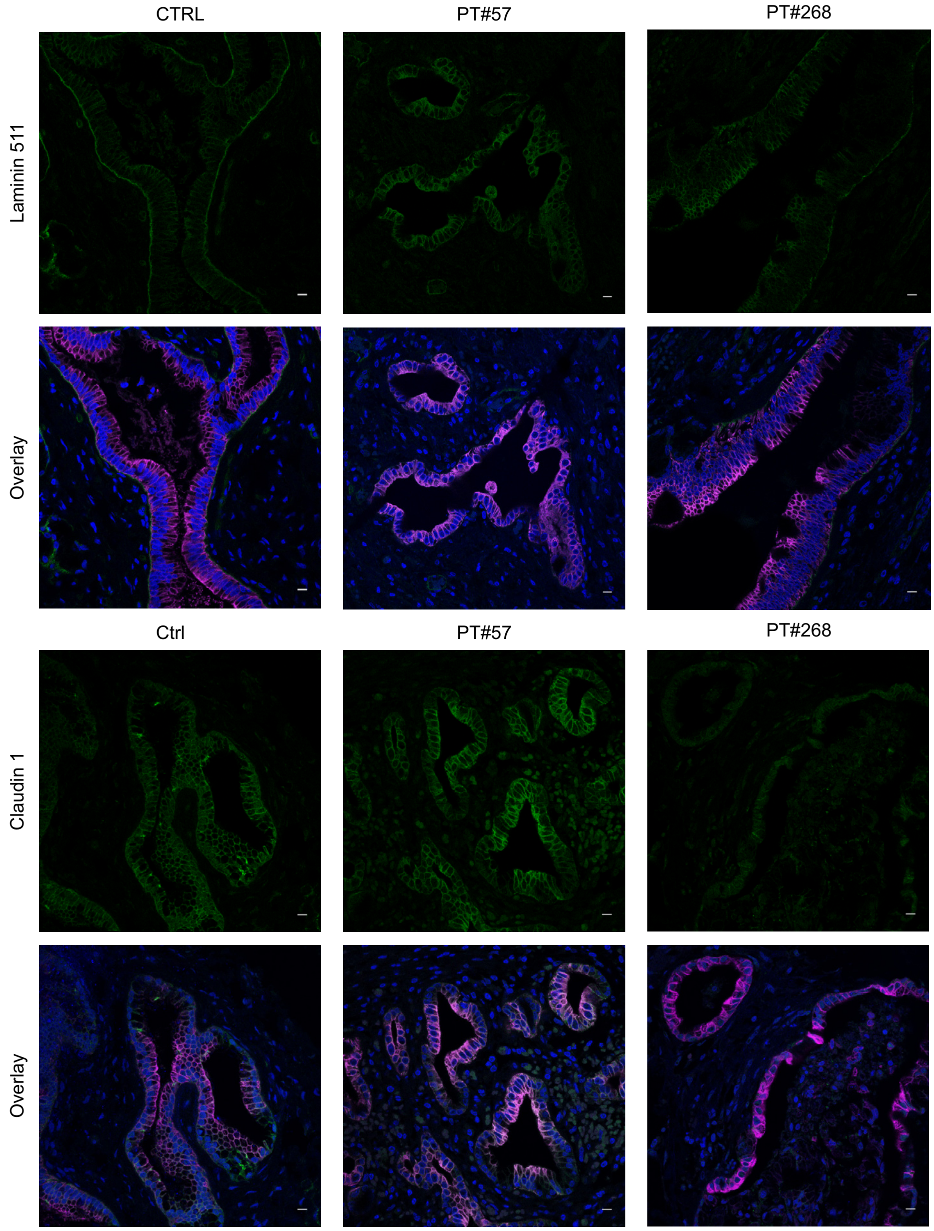

Fig. 8. Laminin 511 and claudin 1 staining may be altered in extrahepatic bile ducts of patients with IRC who were anti-laminin 511-E8 positive. Immunofluorescent staining of (top panels) laminin 511 and (bottom panels) claudin 1 alone (green) and as an overlay with cytokeratin 7 (magenta) and Hoechst (blue) in one control and two patients with IRC who were anti-laminin 511-E8 positive (representative images of three fields per condition, Fig. S6 for all fields). Scale bar = 10 μm. CTRL, control; PT, patient.

Abbreviations

AIP, autoimmune pancreatitis; ANO1, anoctamin 1; *ALB*, albumin; ALP, alkaline phosphatase; ALT, alanine aminotransferase; *ASGR1*, asialoglycoprotein receptor 1; AST, aspartate aminotransferase; BSA, bovine serum albumin; CA19.9, cancer antigen 19.9; CCA, cholangiocarcinoma; *CFTR*, cystic fibrosis transmembrane conductance regulator; CRC, colorectal carcinoma; CRP, C-reactive protein; DPMI, disintegrations per minute; ECO, extrahepatic cholangiocyte organoid; *EPCAM*, epithelial cell adhesion molecule; ERCP, endoscopic retrograde cholangiopancreaticography; ESR, erythrocyte sedimentation rate; FITC, fluorescein isothiocyanate; GCDC, glycochenodeoxycholic acid; GEO, gene expression omnibus; gGT, gamma-glutamyl transferase; *GPBAR1*, G-protein coupled bile acid receptor 1; GSEA, gene set enrichment analysis; HISORt, histology, imaging, serology, other organ involvement, response to therapy; HPB, hepatopancreatobiliary; HRP, horseradish peroxidase; ICO, intrahepatic cholangiocyte organoid; IgG4-RD, IgG4-related disease; IMDM, Iscove's modified Dulbecco's medium; iPSC, induced pluripotent stem cell; IRC, IgG4-related cholangitis; KRT19, cytokeratin 19; *LAMA5*, laminin alpha 5 chain; *LAMB1*, laminin beta 1 chain; *LAMC1*, laminin gamma 1 chain; MDS, multidimensional scaling; MSigDB, molecular signatures database; PBMCs, peripheral blood mononuclear cells; pHi, intracellular pH; PMA, phorbol-12-myristate-13-acetate; PPPD, pylorus-preserving pancreatoduodenectomy; PSC, primary sclerosing cholangitis; SCTR, secretin receptor; shRNA, short hairpin RNA; TBS, Tris-buffered saline; TBST, Tris-

buffered saline with Tween; Th, T helper cell; UDCA, ursodeoxycholic acid; WT, wild type.

Financial support

This study was supported by a ZonMw Open Competition grant (to UB), a South African PSC patient foundation grant (Stichting AMC Foundation #20837 to UB), a Gastrostart grant of the Netherlands Society of Gastroenterology (NVGE) and the Netherlands Association for the Study of the Liver (NASL) (to DT and RK), and an Amsterdam UMC/AMC PhD Scholarship (to DT). DT, RK, DTo, AJ, and SvdG have nothing to disclose. UB received lecture and consulting fees from Abacus, Behring, GSK and Zambon.

Conflicts of interest

The authors declare no conflicts of interest with regard to this work.

Please refer to the accompanying ICMJE disclosure forms for further details.

Authors' contributions

Conceived the experiments: DT, RK, SvdG, UB. Performed the experiments: DT, RK, DTo. Analysed the data: DT, RK, DTo, SvdG, UB. Wrote the original draft of the manuscript: DT, RK, UB. Reviewing and editing: all authors. Our RNA sequencing dataset analysis and graphica display: AJ, DT, RK. Approval of the final manuscript: all authors. Funding facilitation: DT, RK, UB.

Data availability statement

RNA sequencing data were submitted to NCBI Gene Expression Omnibus (GEO) and are freely available under GEO accession number GSE221746.

Acknowledgements

We gratefully acknowledge Dr Perry D. Moerland for recommendations on the analysis of datasets obtained from Gene Expression Omnibus (GEO) and Dr Jan Koster for making use of the R2 genomics analysis and visualization platform (http://r2.amc.nl/). We also acknowledge N. Aizarani and D. Grün (GEO accession GSE124395), and F. Roos, H. Wu, M. Verstegen and L. van der Laan (GEO accession GSE156519) for kindly depositing their RNA sequencing datasets in GEO available to the public. We also acknowledge Prof. Dr Joanne Verheij for support in obtaining extrahepatic bile duct tissue from the Amsterdam UMC HPB Pathology biobank and Esther Vogels for cutting the tissue sections for downstream immunofluorescent staining and imaging. The Graphical abstract was created with BioRender.com.

Supplementary data

Supplementary data to this article can be found online at https://doi.org/10.1016/j.jhepr.2024.101015.

References

Author names in bold designate shared co-first authorship.

[1] Katz G, Stone JH. Clinical perspectives on IgG4-related disease and its classification. Annu Rev Med 2022;73:545–562.
[2] Löhr J-M, Vujasinovic M, Rosendahl J, et al. IgG4-related diseases of the digestive tract. Nat Rev Gastroenterol Hepatol 2022;19:185–197.
[3] **Roos E**, **Hubers LM**, Coelen RJS, et al. IgG4-associated cholangitis in patients resected for presumed perihilar cholangiocarcinoma: a 30-year tertiary care experience. Am J Gastroenterol 2018;113:765–772.
[4] **Maillette de Buy Wenniger LJ**, **Doorenspleet ME**, Klarenbeek PL, et al. Immunoglobulin G4+ clones identified by next-generation sequencing dominate the B cell receptor repertoire in immunoglobulin G4 associated cholangitis. Hepatology 2013;57:2390–2398.
[5] **Mattoo H**, **Mahajan VS**, Della-Torre E, et al. De novo oligoclonal expansions of circulating plasmablasts in active and relapsing IgG4-related disease. J Allergy Clin Immunol 2014;134:679–687.
[6] Wallace ZS, Mattoo H, Carruthers M, et al. Plasmablasts as a biomarker for IgG4-related disease, independent of serum IgG4 concentrations. Ann Rheum Dis 2015;74:190–195.
[7] Hubers L, Vos H, Schuurman A, et al. Annexin A11 is targeted by IgG4 and IgG1 autoantibodies in IgG4-related disease. Gut 2018;67.
[8] Shiokawa M, Kodama Y, Sekiguchi K, et al. Laminin 511 is a target antigen in autoimmune pancreatitis. Sci Transl Med 2018;10.
[9] Perugino CA, AlSalem SB, Mattoo H, et al. Identification of galectin-3 as an autoantigen in patients with IgG4-related disease. J Allergy Clin Immunol 2019;143:736–745.e6.
[10] Shiokawa M, Kodama Y, Kuriyama K, et al. Pathogenicity of IgG in patients with IgG4-related disease. Gut 2016;65:1322–1332.
[11] **Liu H**, **Perugino CA**, Ghebremichael M, et al. Disease severity linked to increase in autoantibody diversity in IgG4-related disease. Arthritis Rheumatol (Hoboken, NJ) 2020;72:687–693.
[12] **Herta T**, **Kersten R**, Chang J-C, et al. Role of the IgG4-related cholangitis autoantigen annexin A11 in cholangiocyte protection. J Hepatol 2022;76: 319–331.
[13] **Hohenester S**, **Maillette de Buy Wenniger LJ**, Paulusma CC, et al. A biliary HCO3- umbrella constitutes a protective mechanism against bile acid-induced injury in human cholangiocytes. Hepatology 2012;55:173–183.
[14] Beuers U, Hohenester S, Maillette de Buy Wenniger LJ, et al. The biliary HCO(3)(-) umbrella: a unifying hypothesis on pathogenetic and therapeutic aspects of fibrosing cholangiopathies. Hepatology 2010;52:1489–1496.
[15] Takayama K, Mitani S, Nagamoto Y, et al. Laminin 411 and 511 promote the cholangiocyte differentiation of human induced pluripotent stem cells. Biochem Biophys Res Commun 2016;474:91–96.
[16] Aumailley M. The laminin family. Cell Adh Migr 2013;7:48–55.
[17] Müller T, Beutler C, Picó AH, et al. Increased T-helper 2 cytokines in bile from patients with IgG4-related cholangitis disrupt the tight junction-associated biliary epithelial cell barrier. Gastroenterology 2013;144:1116–1128.
[18] Mattoo H, Stone JH, Pillai S. Clonally expanded cytotoxic CD4+ T cells and the pathogenesis of IgG4-related disease. Autoimmunity 2017;50:19–24.
[19] Song J, Zhang X, Buscher K, et al. Endothelial basement membrane laminin 511 contributes to endothelial junctional tightness and thereby inhibits leukocyte transmigration. Cell Rep 2017;18:1256–1269.
[20] Sixt M, Engelhardt B, Pausch F, et al. Endothelial cell laminin isoforms, laminins 8 and 10, play decisive roles in T cell recruitment across the blood-brain barrier in experimental autoimmune encephalomyelitis. J Cell Biol 2001;153:933–946.
[21] Wu C, Ivars F, Anderson P, et al. Endothelial basement membrane laminin alpha5 selectively inhibits T lymphocyte extravasation into the brain. Nat Med 2009;15:519–527.
[22] **Zhang X**, **Wang Y**, **Song J**, et al. The endothelial basement membrane acts as a checkpoint for entry of pathogenic T cells into the brain. J Exp Med 2020:217.
[23] Ghazale A, Chari ST, Zhang L, et al. Immunoglobulin G4-associated cholangitis: clinical profile and response to therapy. Gastroenterology 2008;134:706–715.
[24] European Association for the Study of the Liver. EASL clinical Practice Guidelines on sclerosing cholangitis. J Hepatol 2022;77:761–806.
[25] Grubman SA, Perrone RD, Lee DW, et al. Regulation of intracellular pH by immortalized human intrahepatic biliary epithelial cell lines. Am J Physiol 1994;266:G1060–G1070.
[26] Fuss IJ, Kanof ME, Smith PD, et al. Isolation of whole mononuclear cells from peripheral blood and cord blood. Curr Protoc Immunol 2009. Chapter 7:7.1.1–8.
[27] Kizhakeyil A, Ong ST, Fazil MHUT, et al. Isolation of human peripheral blood T-lymphocytes. Methods Mol Biol 2019;1930:11–17.
[28] Chatila T, Silverman L, Miller R, et al. Mechanisms of T cell activation by the calcium ionophore ionomycin. J Immunol 1989;143:1283–1289.
[29] Seppen J, Rijnberg M, Cooreman MP, et al. Lentiviral vectors for efficient transduction of isolated primary quiescent hepatocytes. J Hepatol 2002;36:459–465.
[30] Chang J, Go S, de Waart D, et al. Soluble adenylyl cyclase regulates bile salt-induced apoptosis in human cholangiocytes. Hepatology 2016;64:522–534.
[31] **Hubers LM**, **Schuurman AR**, Buijs J, et al. Blue-collar work is a risk factor for developing IgG4-related disease of the biliary tract and pancreas. JHEP Rep Innov Hepatol 2021;3:100385.
[32] **Maillette de Buy Wenniger LJ**, **Culver EL**, Beuers U. Exposure to occupational antigens might predispose to IgG4-related disease. Hepatology 2014;60:1453–1454.
[33] Löhr J-M, Beuers U, Vujasinovic M, et al. European Guideline on IgG4-related digestive disease – UEG and SGF evidence-based recommendations. United Eur Gastroenterol J 2020;8:637–666.
[34] Trampert DC, van de Graaf SFJ, Jongejan A, et al. Hepatobiliary acid-base homeostasis: insights from analogous secretory epithelia. J Hepatol 2021;74:428–441.

[35] **Zimmer AM**, **Pan YK**, Chandrapalan T, et al. Loss-of-function approaches in comparative physiology: is there a future for knockdown experiments in the era of genome editing? J Exp Biol 2019;222.
[36] El-Brolosy MA, Stainier DYR. Genetic compensation: a phenomenon in search of mechanisms. Plos Genet 2017;13:e1006780.
[37] **Kersten R**, **Trampert DC**, Herta T, et al. IgG4-related cholangitis – a mimicker of fibrosing and malignant cholangiopathies. J Hepatol 2023;79:1502–1523.
[38] Motallebnejad P, Azarin SM. Chemically defined human vascular laminins for biologically relevant culture of hiPSC-derived brain microvascular endothelial cells. Fluids Barriers CNS 2020;17:54.
[39] Kangwantas K, Pinteaux E, Penny J. The extracellular matrix protein laminin-10 promotes blood-brain barrier repair after hypoxia and inflammation in vitro. J Neuroinflammation 2016;13:25.
[40] Wu C, Ivars F, Anderson P, et al. Endothelial basement membrane laminin alpha5 selectively inhibits T lymphocyte extravasation into the brain. Nat Med 2009;15:519–527.
[41] **Ko SBH**, **Mizuno N**, Yatabe Y, et al. Corticosteroids correct aberrant CFTR localization in the duct and regenerate acinar cells in autoimmune pancreatitis. Gastroenterology 2010;138:1988–1996.
[42] Yoshikawa T, Minaga K, Hara A, et al. Disruption of the intestinal barrier exacerbates experimental autoimmune pancreatitis by promoting the translocation of *Staphylococcus sciuri* into the pancreas. Int Immunol 2022;34:621–634.
[43] Min S-N, Wu L-L, Zhang Y-Y, et al. Disruption of tight junction structure contributes to secretory dysfunction in IgG4-related sialadenitis. J Mol Histol 2020;51:33–46.
[44] Abe A, Takano K, Kojima T, et al. Interferon-gamma increased epithelial barrier function via upregulating claudin-7 expression in human submandibular gland duct epithelium. J Mol Histol 2016;47:353–363.
[45] Omokawa A, Komatsuda A, Hirokawa M, et al. Membranous nephropathy with monoclonal IgG4 deposits and associated IgG4-related lung disease. Clin Kidney J 2014;7:475–478.

Journal of Hepatology, Volume *6*

Supplemental information

Laminin 511-E8, an autoantigen in IgG4-related cholangitis, contributes to cholangiocyte protection

David C. Trampert, Remco Kersten, Dagmar Tolenaars, Aldo Jongejan, Stan F.J. van de Graaf, and Ulrich Beuers

Laminin 511-E8, an autoantigen in IgG4-related cholangitis, contributes to cholangiocyte protection

David C. Trampert, Remco Kersten, Dagmar Tolenaars, Aldo Jongejan,

Stan F.J. van de Graaf, Ulrich Beuers

Table of contents

Supplementary methods……………………………………………………………….…....2

Supplementary figures…………………………………………………………….….….7

Supplementary tables…………………………………………………………………..…20

Supplementary references……………………………………………………………..…25

Supplementary methods

Descriptive statistics for IRC patient cohort and clinical characteristics of anti-laminin 511-E8 positive patients

For the entire IRC patient cohort, clinical characteristics were collected from the hospital electronic health record. In addition, for the entire IRC patient cohort, history of HPB surgery and biopsies including pathology reports containing the details for histological IRC assessment were collected from the hospital's electronic health record. Histological IRC diagnosis was "highly suggestive" when 2 out of 3 major criteria were met with >10 $IgG4^+$ cells / HPF in resection specimens or biopsies [1-2]. Histological IRC diagnosis was "probable" when 1 out of 3 major criteria was met with >10 $IgG4^+$ cells / HPF, otherwise cases had "insufficient evidence".

For IRC patients who were positive for anti-laminin 511-E8 autoantibodies, detailed collected parameters included gender, age at disease onset, time to diagnosis, organ involvement, profession including > 1 year of potential blue-collar work, positivity to other autoantibodies, liver biochemistry, history of malignancies or major hepatopancreatobiliary surgery and therapeutic management of IgG4-RD. To illustrate the disease courses of anti-laminin 511-E8 positive IRC patients, liver parameters (total bilirubin, ALP, gGT, AST and ALT) and IRC-associated therapy (ERCP, PTC drainage, hepatopancreatobiliary surgery, ursodeoxycholic acid, prednisolone, 6-thioguanine, azathioprine) were extracted from the patient's electronic health record and graphed accordingly.

Cell culture and differentiation of LX2

The human hepatic stellate cell line LX2, described by Xu et al. was treated with 10 ng/ml human TGFβ for 3 days to transdifferentiate cells into activated myofibroblasts as previously described [3-4]. LX2 culture medium consisted of Dulbecco's Modified Eagle Medium (DMEM) supplemented with 10% FBS and 37.5 U/ml (1%) penicillin, 37.5 µg/ml (1%) streptomycin.

RNA sequencing

H69 cholangiocytes were seeded at a density of 100.000 cells in 12-well plates and grown for 48 hours to reach confluency. Then treatment commenced with 0.25 $\mu g/cm^2$ recombinant human laminin 511-E8 for 48 hours. Growth media with treatment was

refreshed 24 hours after the start of treatment. RNA was isolated from four treated and four untreated wells after 48 hours of treatment using QIAzol lysis reagent (Qiagen) according to the spin column miRNeasy Mini Kit (Qiagen) as previously used for total RNA isolation and downstream bulk RNA sequencing [5]. RNA concentration and quality were assessed by the Core Facility Genomics (Amsterdam UMC) using the RNA ScreenTape. Concentrations were in the range of 100-300 ng/µl and RNA integrity scores (RIN) were 9-10. Three biological replicates with the best RIN score and concentration range were sent in for library preparation by KAPA mRNA Hyperprep. The RNA samples were enriched for mRNA by poly(A) mRNA capture with a library length from 150 – 850 base pairs. Samples were pair-end sequenced (150bp) on the NovaSeq (Illumina) with a sequencing depth of 40 million reads per sample.

Raw FASTQ files were obtained and adapter sequences were trimmed using Trimmomatic v0.39 [6]. Sequences were aligned against the human reference genome (GRCh38) using HISAT2 (v2.2.1) [7]. Quality control was performed using FastQC v0.11.9 and dupRadar v1.12.1 [8]. Count tables were made using HTSeq v0.11.0 [9] and the corresponding GTF. Genes with more than 2 counts-per-million reads (CPM) in 2 or more of the samples were kept. Counts were normalized as previously described [10]. Genes were reannotated using Ensembl (v105) with biomaRt. Count data was transformed to log2-counts per million (logCPM). No clear sample outliers were identified based on library size or normalization factors. MDS plots were generated showing a clear separation based on the condition with more variation between the control samples than the laminin 511-E8 treated samples. Differential expression was assessed using an empirical Bayes moderated t-test within limma's linear model framework including the precision weights estimated by voom [11-12]. Cell line effects were corrected for by incorporating it in the design. Benjamini-Hochberg false discovery rate was used to correct for multiple testing of the resulting p-values. Analysis was performed using R v4.1.0 and Bioconductor v3.13. Gene set enrichment analysis (GSEA) was performed using the CAMERA [13] function as implemented in limma (using an inter-gene correlation of 0.01) with gene sets from MSigDB v7.4 (H,C1,C2,C3,C5,C6,C7,C8). RNA sequencing data was submitted to NCBI Gene Expression Omnibus (GEO) and is freely available under GEO accession number GSE221746.

Single cell RNA sequencing data on the human liver was obtained from GEO accession number GSE124395. Clustering was performed as described ending up with a mature cholangiocyte population (*EPCAM, KRT19, CFTR* +) in clusters 4 and 7, and a hepatocyte population (*ALB, ASGR1* +) in cluster 11 [14]. Within the mesenchymal cell population, periductal (myo)fibroblasts were identified in clusters 9 and 10 based on a recently described murine gene panel consisting of *Col15a1, Cd34, Thy1, Gli1, Clec3b, Fbln2, Entpd2* [15].

RNA isolation, cDNA synthesis, real-time quantitative PCR, primer design and validation

Total RNA was isolated from H69 cholangiocytes using TRIzol reagent (Sigma). With chloroform and centrifugation, the aqueous phase was isolated. RNA was recovered by precipitating with isopropyl alcohol. RNA pellets were washed with 70% ethanol and resuspended in diethylpyrocarbonate (DEPC) treated water. Quality (A260/A280 >1.8) and concentration of RNA samples were checked by spectrophotometry using the Nanodrop 1000 (Thermo Scientific, Waltham, MS).

2 µg of RNA was used as input and treated with DNase I (Promega) followed by reverse transcription into cDNA using Oligo-dT (Invitrogen), Random Hexamer primers (Promega) and Revertaid transcriptase (Fermentas) resulting in 20 µl cDNA. cDNA was diluted to 100 µl, after which 2 µl served as a template for real-time quantitative PCR with the SensiFAST SYBR No-ROX kit (Bioline). RT-qPCR plates were run on the Bio-Rad CXF96. Raw fluorescent values were exported and starting concentration (N0) and cycle quantification (Cq) values were obtained using LinRegPCR v.2013.0 (Academic Medical Center, Amsterdam) [16]. Expression levels were normalized to the geomean of human *36B4* (*RPLP0*) and *HPRT* reference genes.

Primers used for RT-qPCR were self-designed to cover all transcript variants of the gene of interest. To this end FASTA sequences of all transcript variants were obtained and aligned with a nucleotide count using Clustal Omega [17], a multiple sequence alignment tool (https://www.ebi.ac.uk/Tools/msa/clustalo/). NCBI PRIMER-BLAST was then used to design primers restricted to the sequence range identical for all transcript variants (https://www.ncbi.nlm.nih.gov/tools/primer-blast/index.cgi) [18].

Additional requirements were that primers must span an exon-exon junction and ideally the primer pair was separated by at least one intron on the corresponding genomic DNA. Subsequently primers were validated by assessing the linear relation between increasing cycle quantification (Cq) values with serial dilution of cDNA. Melting temperatures (Tm) of the designed primers were checked after the RT-qPCR run with a melting curve analysis. Finally, the amplified PCR product was loaded on an agarose gel and separated by electrophoresis alongside a standard ladder to confirm the size of the amplified product corresponded with the size of the target PCR product.

Intracellular pH measurement by 2',7'-bis-(2-carboxyethyl)-5-(and-6)-carboxyfluorescein acetoxymethyl ester (BCECF AM)

The principle of this assay is performed as previously described with minor custom changes [19]. H69 cholangiocytes were plated in 96-well black solid flat bottom tissue culture treated plates (Corning, Costar #3916) and grown until confluency. For one 96-well plate a working solution of 5 μM BCECF AM (Sigma, Invitrogen B1150) was prepared in 5 ml of 20 mM HEPES-buffered Hank's Balanced Salt Solution (HBSS) without phenol red at pH 7.4. After a series of washing steps with HEPES-buffered HBSS, cells were loaded with BCECF AM for 45 minutes at room temperature and protected from light. Three wells per condition / cell line were left unloaded for blank measurements. Excessive dye was removed and the plate was incubated for an additional 30 minutes in HEPES-buffered HBSS.

A pH standard was prepared from a high-potassium Nigericin (10 μM) solution supplemented with 30 mM HEPES covering the pH range from 5.8 to 8.6. The pH standards were placed in a water bath at 37 degrees Celsius, after which the pH was measured of each standard. BCECF AM loaded wells from the 96-well plate were filled with the pH standards and experimental solutions were added to the loaded and blank wells of each condition. The plate was placed in the CLARIOstar (BMG LABTECH, Ortenberg, Germany) set at 37 degrees and a gain adjustment for all wells was performed aiming for 50% of maximal fluorescent values. Ratiometric fluorescent measurement was performed at dual excitation wavelength: $\lambda Ex1$= 490-10 nm (pH-sensitive) / $\lambda Ex2$= 440-10 nm (pH-insensitive), emission was collected at λEm= 535-20 nm. pH standards were measured until a steady state was acquired. After this,

experimentally treated wells were measured at 37 degrees with 5% CO_2 inflow again until a steady state was acquired.

Raw data of the experiment were converted to fluorescent ratios. Prior to this, background fluorescence from the unloaded blank wells were subtracted from the corresponding BCECF AM loaded conditions at all time points. The fluorescent ratio R was calculated as F490/440. Fluorescent ratios were converted to a true pH using the fluorescent ratios from the high-potassium Nigericin pH standards. In brief, pH values were obtained by performing nonlinear least-square fitting according to the following equation as previously described [20-21]: $pH = pK_a - \log\frac{R - R_A}{R_B - R} \times \frac{F_{A(\lambda 2)}}{F_{B(\lambda 2)}}$, where R stands for F490/440, R_A and R_B represent fluorescent ratios at extreme acidic and basic conditions respectively, $F_{A(\lambda 2)}$ and $F_{B(\lambda 2)}$ are the fluorescent values λEx2 440 nm at extreme acidic and basic conditions respectively. Baseline intracellular pH values were determined by averaging the pH values of the last eight timepoints.

Supplementary figures

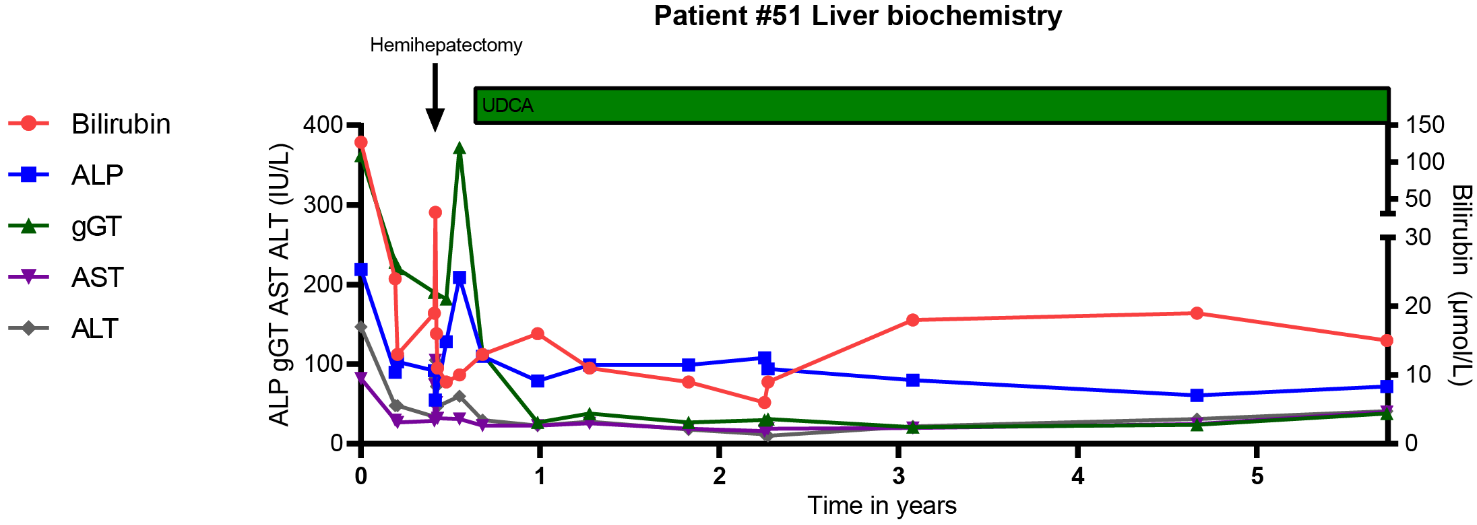

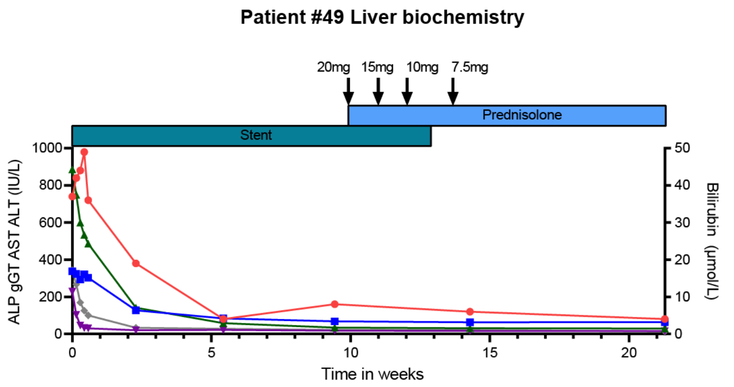

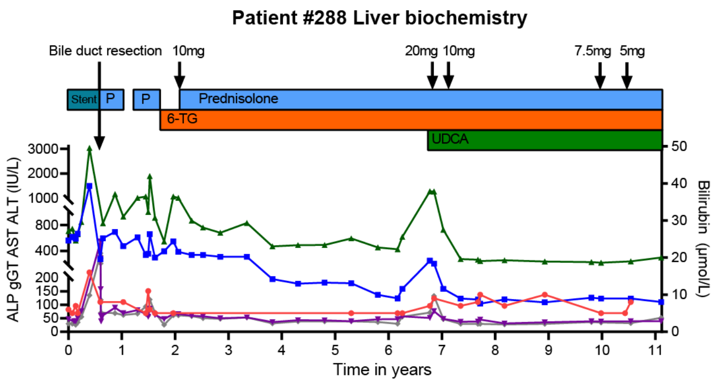

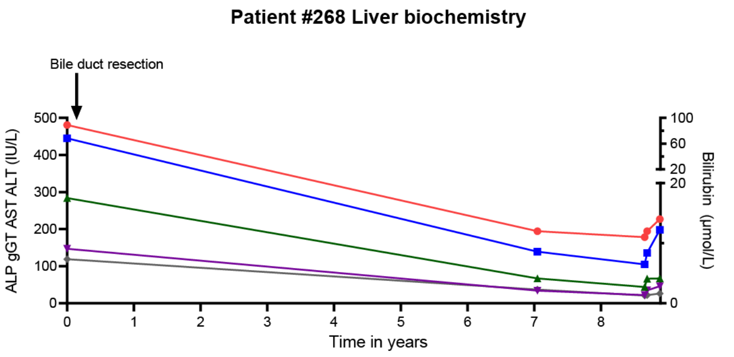

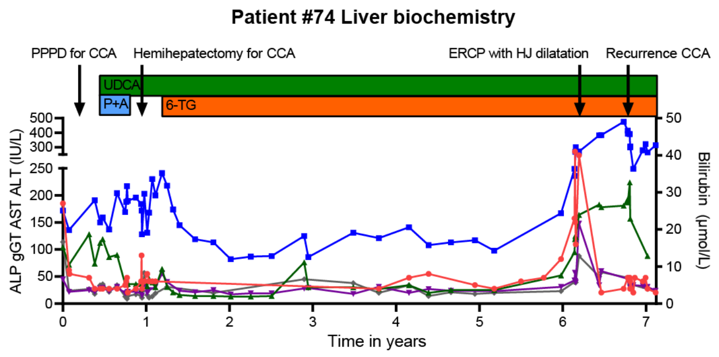

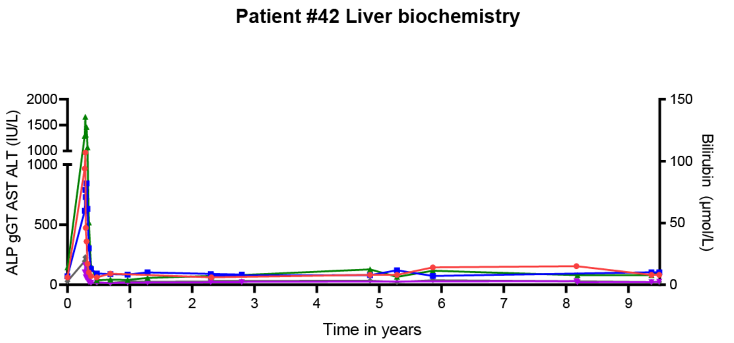

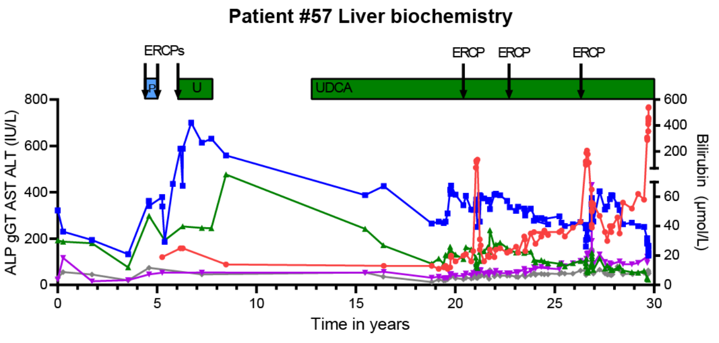

Fig. S1. Liver biochemistry and response to IRC directed therapy in patients positive for anti-laminin 511-E8 autoantibodies. ALP, gGT, AST, ALT left Y-axis. Bilirubin right Y-axis. Therapeutic interventions are indicated with bars and arrows above the graphs. Patient # corresponds to ELISA patient numbers in Figure 1 of the main manuscript. Abbreviations: 6-TG, 6-thioguanine; A, azathioprine; ALP, alkaline phosphatase; ALT, alanine aminotransferase; AST, aspartate aminotransferase; CCA, cholangiocarcinoma; ERCP, endoscopic retrograde cholangiopancreaticography; gGT, gamma-glutamyl transferase; HJ, hepaticojejunostomy; P, prednisolone; PPPD, pylorus-preserving pancreatoduodenectomy; UDCA, ursodeoxycholic acid.

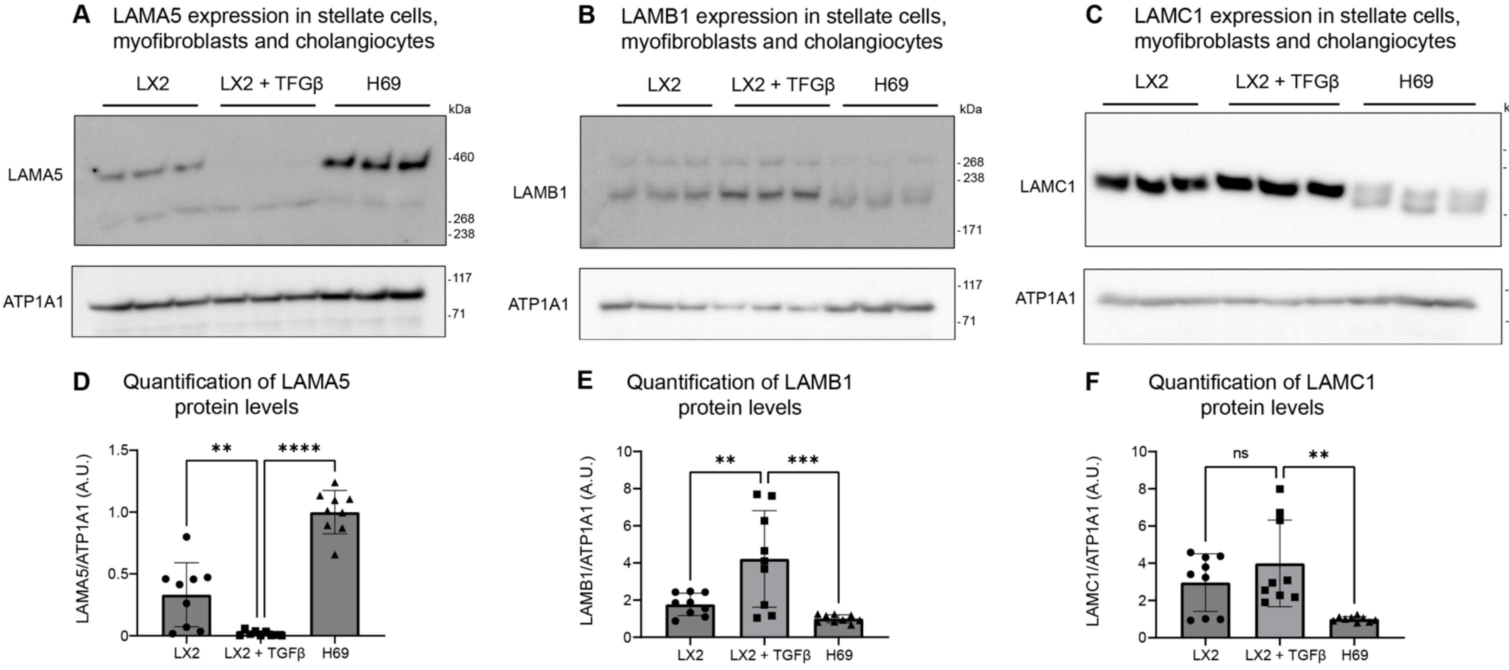

Fig. S2. Laminin 511 constituent protein expression in fibroblast models and cholangiocytes. (A) LAMA5 (400 kDa), (B) LAMB1 (198 kDa), and (C) LAMC1 (178 kDa) expression in naive LX2 (stellate cells), activated LX2 (myofibroblasts), and H69 cholangiocytes. Quantification of (D) LAMA5, (E) LAMB1, and (F) LAMC1 protein levels normalized by ATP1A1 (112 kDa), (9 cell samples from n=3 independent experiments). Data are represented as mean with standard deviation. Levels of significance: (E) – (F) ns = not significant, ** $p < 0.01$, *** $p < 0.001$, **** $p < 0.0001$; unpaired t-test.

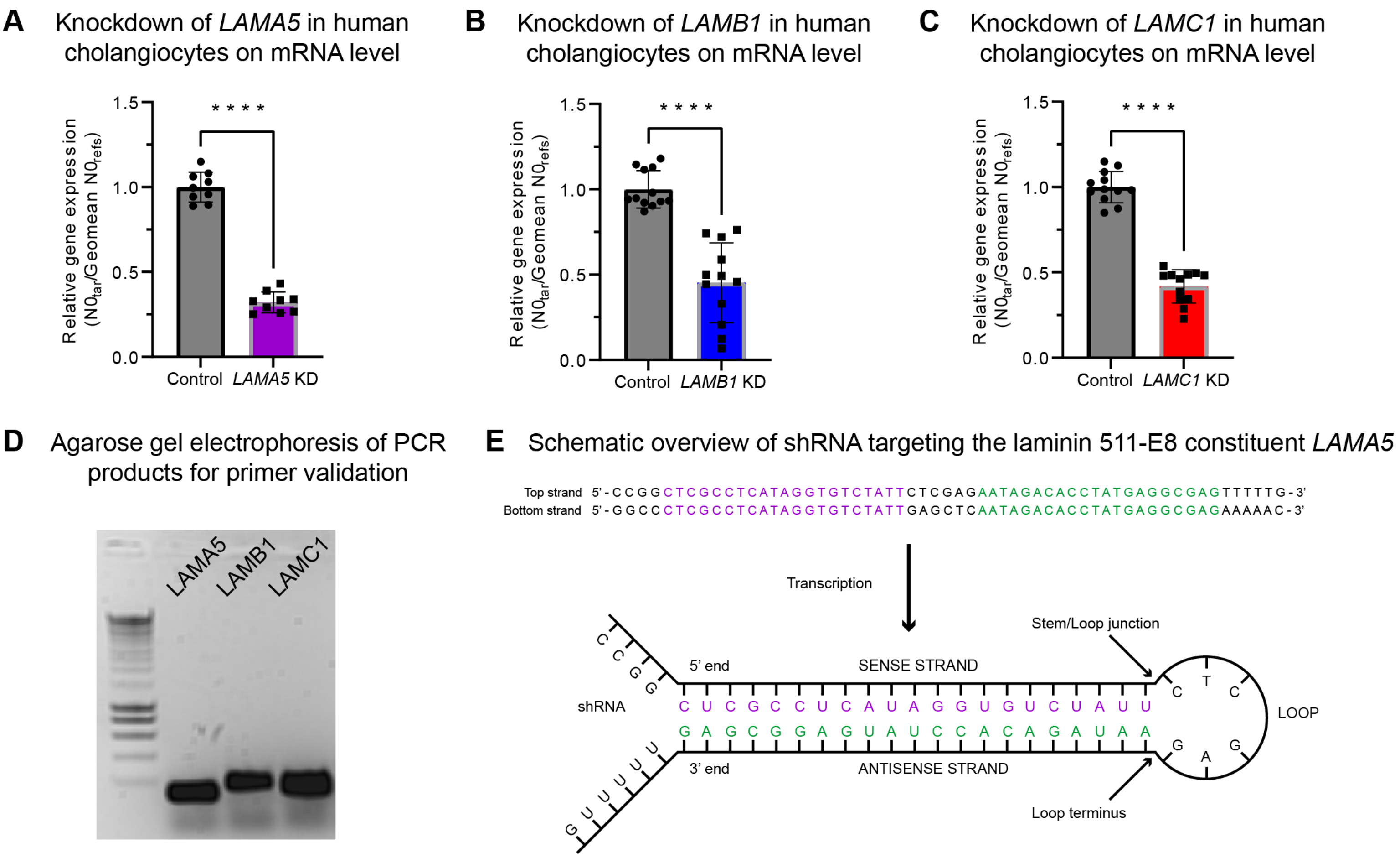

Fig. S3. Knockdown validation of laminin 511 constituents *LAMA5*, *LAMB1*, *LAMC1* by RT-qPCR. Relative mRNA expression in (A) *LAMA5*, (B) *LAMB1*, and (C) *LAMC1* shRNA knockdown human H69 cholangiocytes compared to control cholangiocytes (9-12 cell samples from n=3-4 independent experiments). (D) gel electrophoresis showing PCR products of *LAMA5*, *LAMB1*, and *LAMC1* as part of RT-qPCR primer validation. (E) Schematic of short hairpin RNA with the used sequence of *LAMA5* in place. Data are represented as starting concentration (N0) of target genes over the geomean of the reference genes *36B4* (*RPLP0*) and *HPRT* N0 values normalized to control. Levels of significance: (A) – (C) **** $p < 0.0001$; unpaired t-test. Abbreviations: Control, shRNA control; KD, knockdown; N0, starting concentration; shRNA, short hairpin RNA.

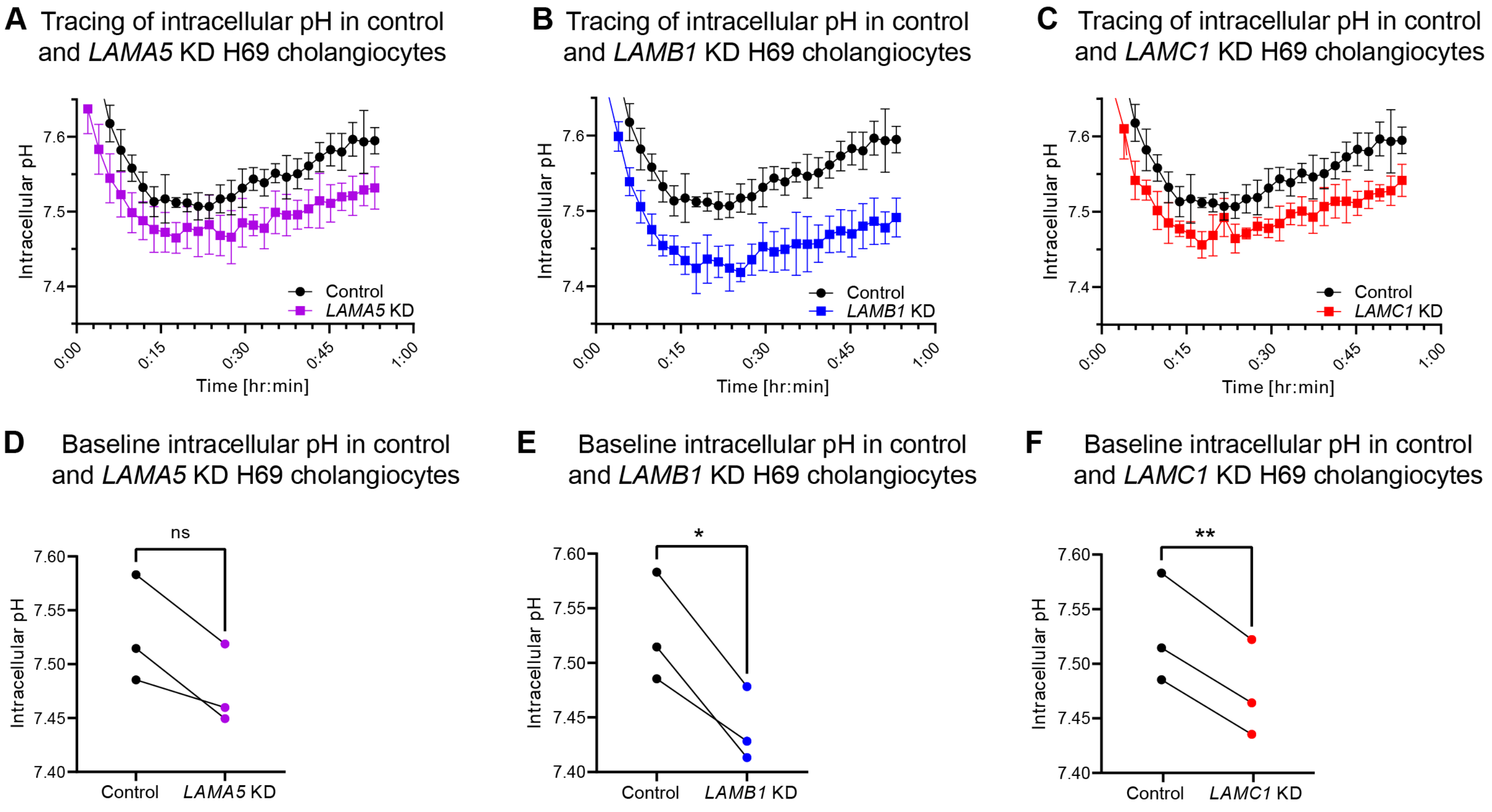

Fig. S4. Knockdown of laminin 511 constituents in H69 cholangiocytes decreases baseline intracellular pH. Intracellular pH tracing in control, (A) *LAMA5*, (B) *LAMB1,* and (C) *LAMC1* knockdown H69 cholangiocytes (representative experiment of n=3). Baseline intracellular pH in control, (D) *LAMA5*, (E) *LAMB1,* and (F) *LAMC1* knockdown cholangiocytes (3 average baseline values from n=3 independent experiments). Data are represented as mean with standard deviation. Levels of significance: (D) – (F) ns = not significant, * $p < 0.05$, ** $p < 0.01$; paired t-test. Abbreviations: Control, shRNA control; KD, knockdown.

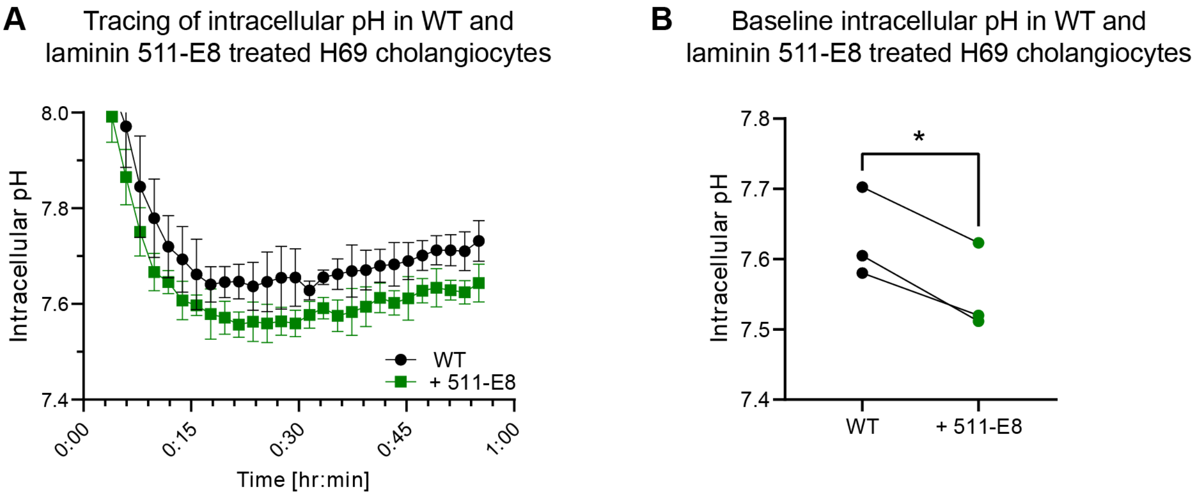

Fig. S5. Recombinant laminin 511-E8 treatment of H69 cholangiocytes decreases baseline intracellular pH. (A) Intracellular pH tracing in wild-type and recombinant laminin 511-E8 treated H69 cholangiocytes (representative tracing from n=3 independent experiments). (B) Baseline intracellular pH in wild-type and recombinant laminin 511-E8 treated H69 cholangiocytes (3 average baseline values from n=3 independent experiments). Data are represented as mean with standard deviation. Levels of significance: (B) * $p < 0.05$; paired t-test. Abbreviations: KD, knockdown; WT, wild-type.

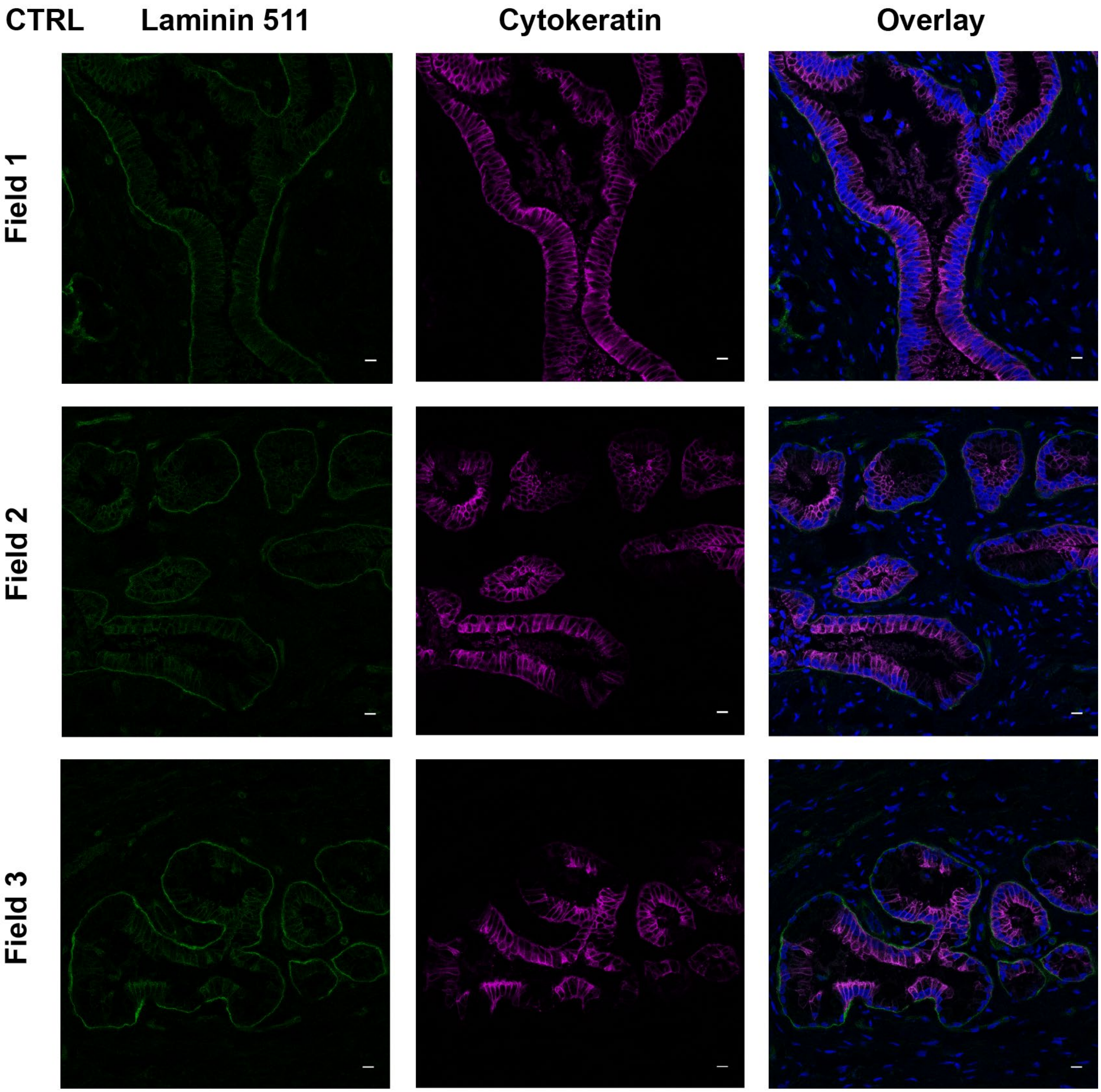
CTRL
Laminin 511
Cytokeratin
Overlay
Field 1
Field 2
Field 3

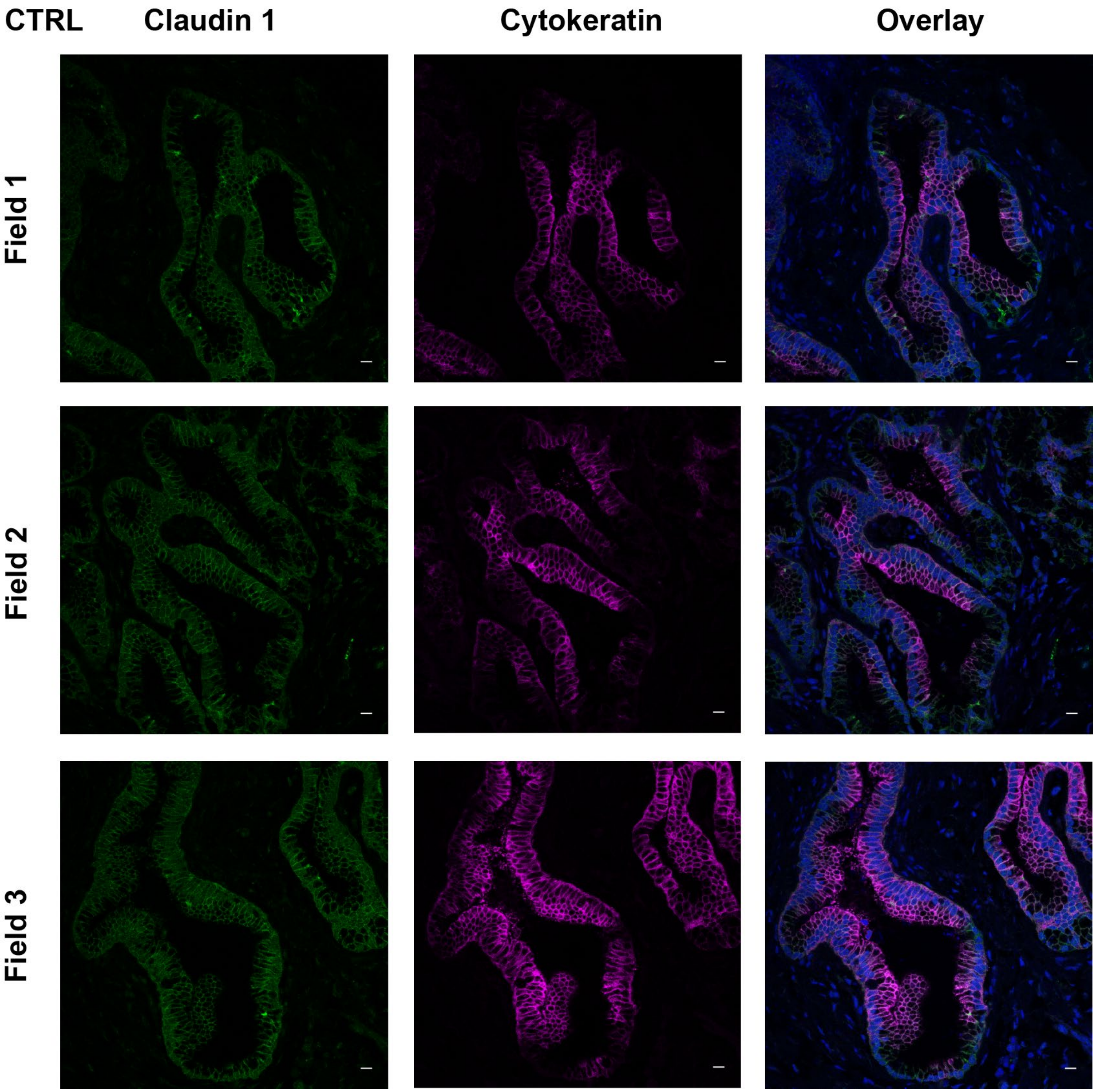
CTRL
Claudin 1
Cytokeratin
Overlay
Field 1
Field 2
Field 3

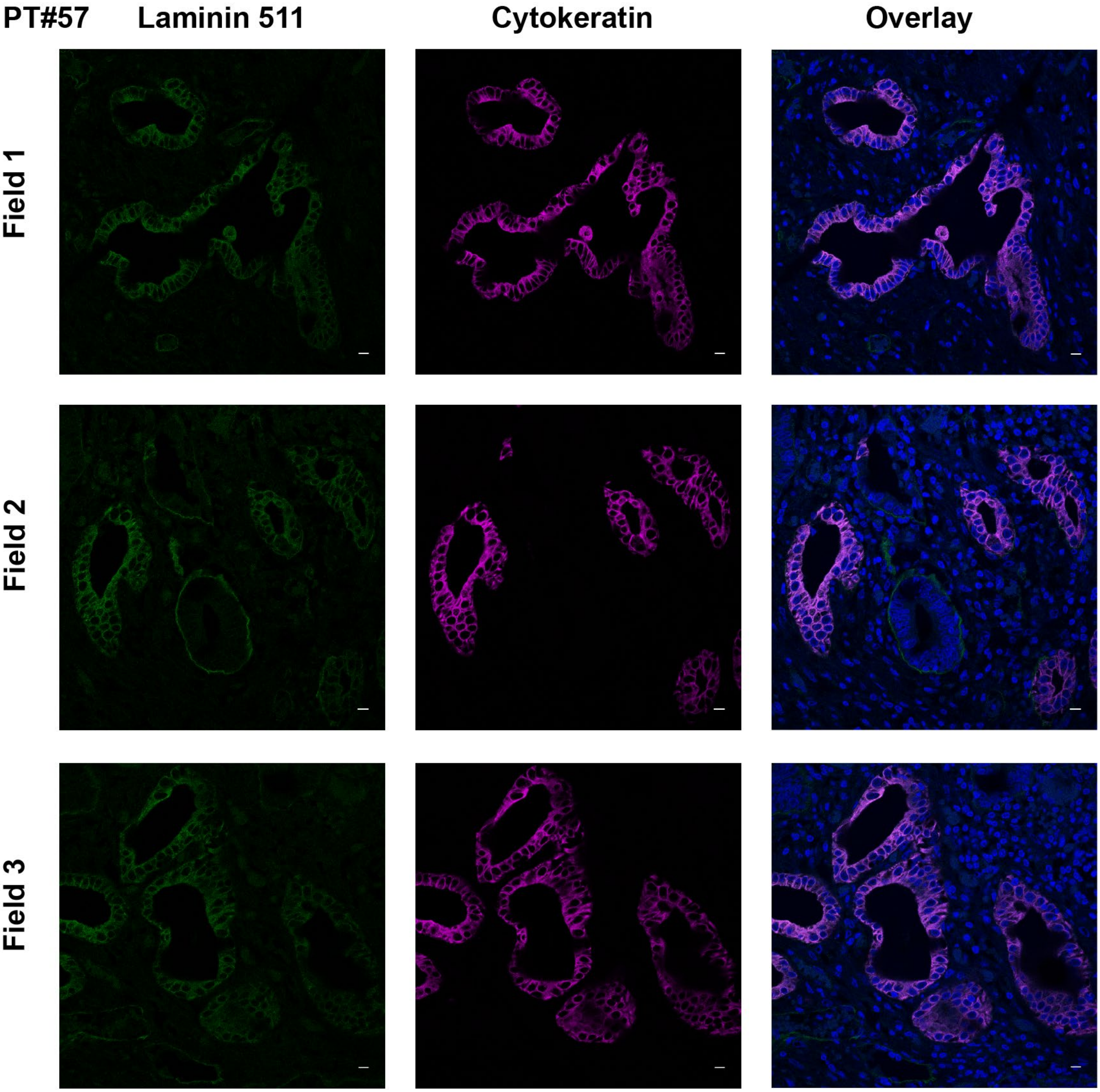

PT#57
Laminin 511
Cytokeratin
Overlay
Field 1
Field 2
Field 3

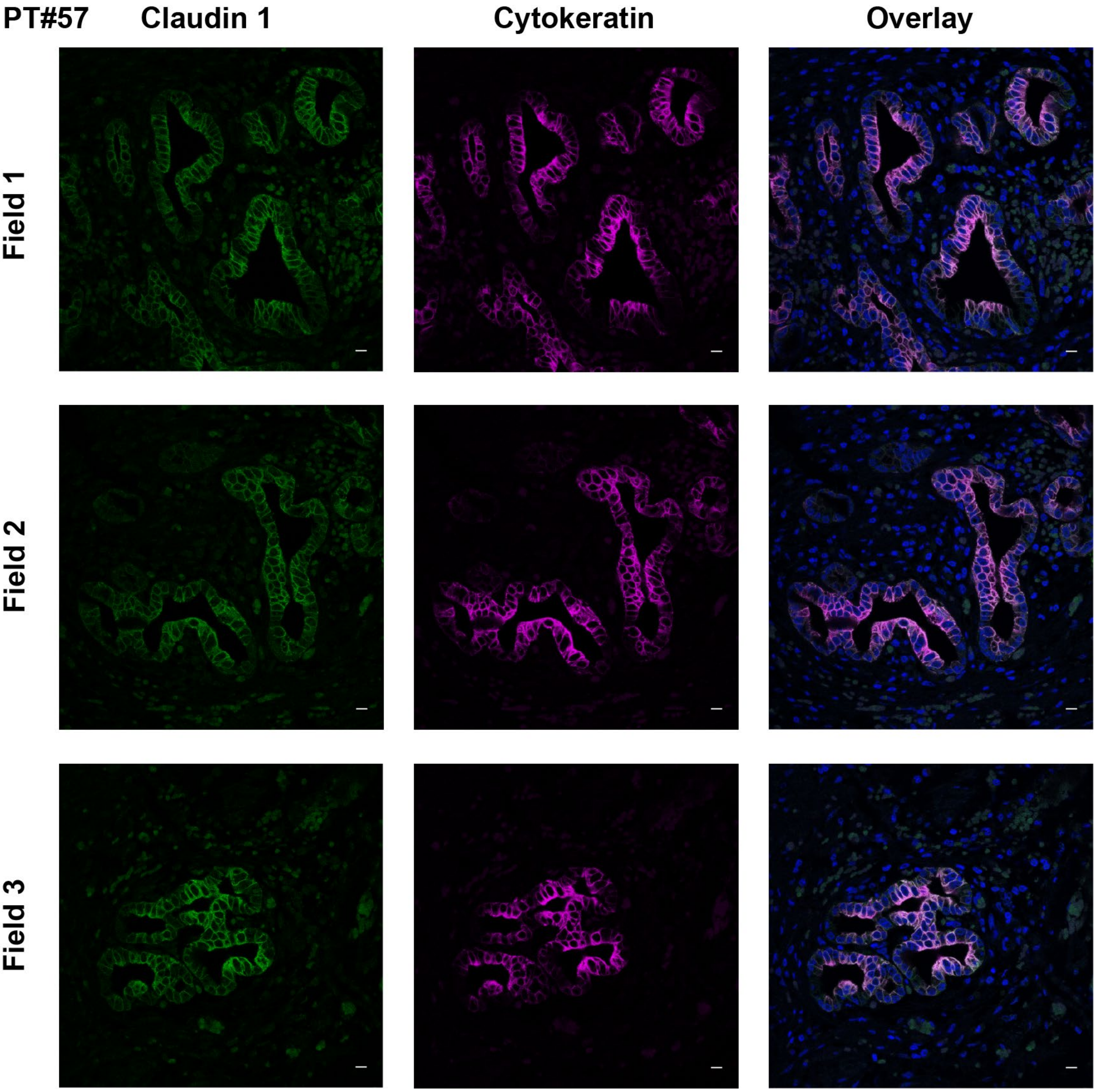
PT#57
Claudin 1
Cytokeratin
Overlay
Field 1
Field 2
Field 3

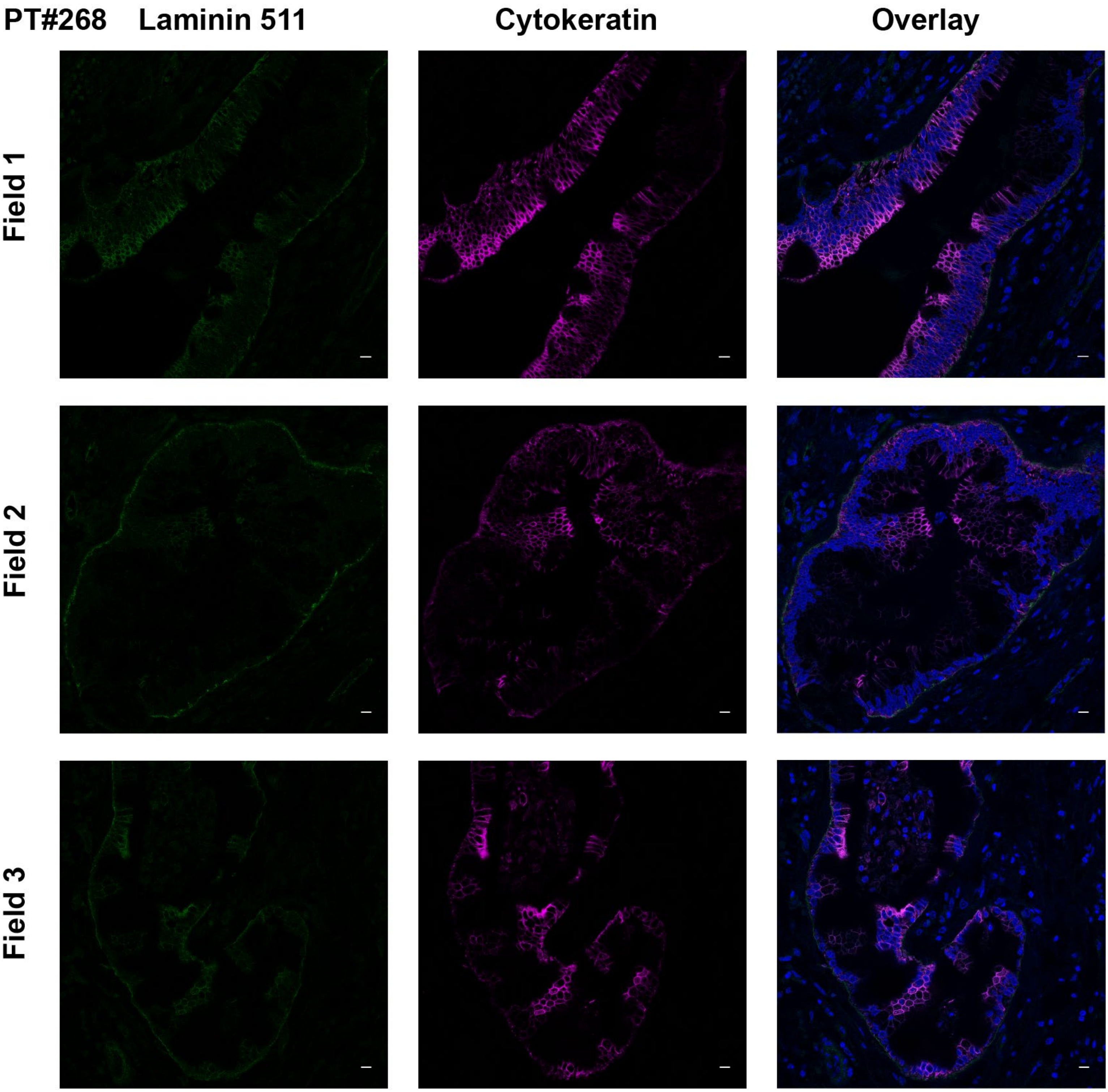
PT#268
Laminin 511
Cytokeratin
Overlay
Field 1
Field 2
Field 3

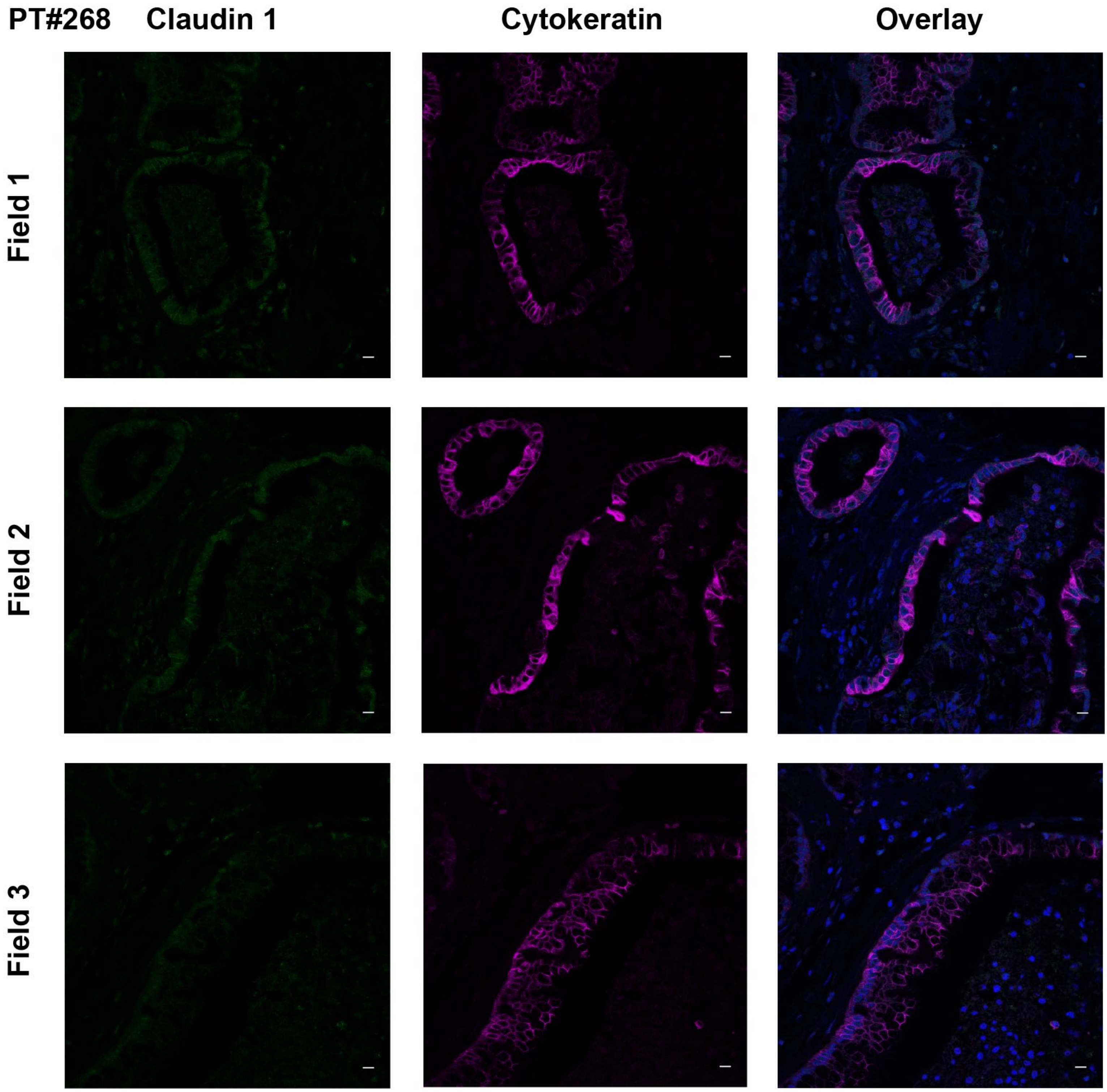

PT#268
Claudin 1
Cytokeratin
Overlay
Field 1
Field 2
Field 3

Fig. S6. Immunofluorescent stainings of laminin 511 and claudin 1 in extrahepatic bile duct sections of control and of IRC patients #57 and #268. In a blinded experiment, three fields of each condition were imaged with a confocal microscope using identical imaging settings. In green laminin 511 and claudin 1 are displayed, in magenta a cytokeratin 7 staining is displayed to identify the biliary tissue. The overlay image contains a nuclear Hoechst staining. Note that there is one structure in field 2 of patient #57 showing a basolateral laminin 511 staining, which is negative for cytokeratin 7, likely being a vascular structure. Representative fields were used for Figure 8 of the main manuscript**.** Scale bar is 10 μm. Abbreviations: CTRL, control; PT, patient.

Supplementary tables

Table S1. Surgical history and histological assessment in IRC patient cohort

Patient #	511-E8 positivity	HPB surgery	Timing of HPB surgery	HPB Biopsy	Indication for surgery/biopsy	Histological IRC criteria assessed	IRC Histological assessment	Lympho-Plasmacellular infiltrate	Obliterative phlebitis	Storiform fibrosis	$IgG4^{+}$/HPF
**74**	**Yes**			Liver biopsy	Suspected CCA with PSC	Yes	Highly suggestive	Yes	No	Yes	>10
		PPPD	Before diagnosis		Suspected CCA	Yes	Highly suggestive	Yes	No	Yes	>10
		Hemihepatectomy	After diagnosis		CCA in background of IRC	No	NA	NA	NA	NA	NA
**51**	**Yes**	Hemihepatectomy	Before diagnosis	No	Suspected CCA	Yes	Highly suggestive	Yes	Yes	Yes	>20
**288**	**Yes**			Liver biopsy	Suspected CCA	No	NA	NA	NA	NA	NA
		Bile duct resection	Before diagnosis	No	Suspected CCA	Yes	Highly suggestive	Yes	Yes	Yes	>10
21	No			Liver biopsy	Suspected CCA	Yes	Insufficient evidence	Yes	No	No	<10
		Hemihepatectomy	Before diagnosis		Suspected CCA	Yes	Highly suggestive	Yes	No	Yes	>10
47	No	Hilar resection	Before diagnosis	No	Suspected CCA	Yes	Highly suggestive	Yes	No	Yes	>75
44	No	Hemihepatectomy	Before diagnosis	No	Suspected CCA	Yes	Highly suggestive	Yes	No	Yes	52
50	No			Liver biopsy		No	NA	NA	NA	NA	NA
		Cholecystectomy	Before diagnosis		Suspected CCA	Yes	Highly suggestive	Yes	No	Yes	>50
148	No	Hemihepatectomy	Before diagnosis	No	Suspected CCA	Yes	Highly suggestive	Yes	No	Yes	20
225	No	Hemihepatectomy	Before diagnosis	No	Suspected CCA	Yes	Highly suggestive	Yes	Yes	Yes	100
269	No			Liver biopsy	Suspected CCA	No	NA	NA	NA	NA	NA
		Bile duct resection	Before diagnosis			Yes	Highly suggestive	Yes	No	Yes	22
262	No	Hilar resection	Before diagnosis	No	Suspected CCA	Yes	Highly suggestive	Yes	No	Yes	13
271	No	Hemihepatectomy	Before diagnosis	No	Suspected CCA	Yes	Highly suggestive	Yes	No	Yes	13

Patient #	511-E8 positivity	HPB surgery	Timing of HPB surgery	HPB Biopsy	Indication for surgery/biopsy	Histological IRC criteria assessed	IRC Histological assessment	Lympho-plasmacellular infiltrate	Obliterative phlebitis	Storiform fibrosis	IgG4+/HPF
22	No	No	NA	Liver biopsy	Elevated liver enzymes e.c.i.	Yes	Probable IRC	No	No	Yes	>10
52	No	Biliodigestive anastomosis	Before diagnosis	No	Suspected pancreatic carcinoma	Yes	Probable IRC	Yes	No	No	>50
**268**	**Yes**	Bile duct resection	Before diagnosis	No	Suspected CCA	Yes	Probable IRC	Yes	No	Yes	8
58	No	No	NA	Liver biopsy	Suspected metastasis, unknown primary tumour	Yes	Insufficient evidence*	Yes	No	Yes	Unreliable staining
4	No	No	NA	Liver biopsy	Suspected CCA, DDx IRC	Yes	Insufficient evidence	No	No	No	Unreliable staining
28	No	No	NA	Liver biopsy	Liver cirrhosis e.c.i.	Yes	Insufficient evidence*	Yes	No	Yes	Not enough material
24	No	No	NA	Ampulla of Vater biopsy	Suspected CCA, DDx IRC	Yes	Insufficient evidence	No	No	No	<10
95	No	No	NA	Liver biopsy	Elevated liver enzymes e.c.i.	No	NA	NA	NA	NA	NA
				Liver biopsy	Elevated liver enzymes, DDx IRC	Yes	Insufficient evidence	NA	NA	NA	<10
54	No	No	NA	Ampulla of Vater biopsy	Bile duct stenosis, DDx IRC	Yes	Insufficient evidence	No	No	No	None
270	No	Hemihepatectomy	Before diagnosis	No	Suspected CCA	Yes	Insufficient evidence	Yes	No	No	3
75	No	No	NA	Liver biopsy	Elevated liver enzymes e.c.i.	Yes	Insufficient evidence	No	No	No	None
233	No	No (autopsy)	After diagnosis	No	NA	Yes	Insufficient evidence	Yes	No	No	13
53	No	Hemihepatectomy	After diagnosis	NA	Liver abscess	NA	NA	NA	NA	NA	NA
6	No	Biliodigestive anastomosis	Before diagnosis	No	Suspected pancreatic tumour	NA	NA	NA	NA	NA	NA
223	No	Biliodigestive anastomosis	Before diagnosis	No	Suspected pancreatic tumour	NA	NA	NA	NA	NA	NA
		PPPD	Before diagnosis	No	Suspected pancreatic tumour	NA	NA	NA	NA	NA	NA
5	No	No	NA	Liver biopsy	Suspected CCA	No	NA	NA	NA	NA	NA
11	No	No	NA	No	NA	NA	NA	NA	NA	NA	NA

Patient #	511-E8 positivity	HPB surgery	Timing of HPB surgery	HPB Biopsy	Indication for surgery/biopsy	Histological IRC criteria assessed	IRC Histological assessment	Lympho-plasmacellular infiltrate	Obliterative phlebitis	Storiform fibrosis	IgG4⁺/HPF
**57**	**Yes**	Cholecystectomy	Before diagnosis		Cholecystolithiasis	No	NA	NA	NA	NA	>20
				Liver biopsy	Elevated liver enzymes e.c.i.	No	NA	NA	NA	NA	NA
				Liver biopsy	DDx PSC, study biopsy	No	NA	NA	NA	NA	NA
				Liver biopsy	DDx PSC, study biopsy	No	NA	NA	NA	NA	NA
80	No	No	NA	Ampulla of Vater biopsy	Suspected metastasis esophageal carcinoma	No	NA	NA	NA	NA	NA
65	No	No	NA	No	NA	NA	NA	NA	NA	NA	NA
67	No	No	NA	No	NA	NA	NA	NA	NA	NA	NA
87	No	No	NA	No	NA	NA	NA	NA	NA	NA	NA
144	No	No	NA	No	NA	NA	NA	NA	NA	NA	NA
147	No	No	NA	No	NA	NA	NA	NA	NA	NA	NA
158	No	No	NA	No	NA	NA	NA	NA	NA	NA	NA
160	No	No	NA	No	NA	NA	NA	NA	NA	NA	NA
170	No	No	NA	No	NA	NA	NA	NA	NA	NA	NA
172	No	No	NA	No	NA	NA	NA	NA	NA	NA	NA
157	No	No	NA	No	NA	NA	NA	NA	NA	NA	NA
17	No	No	NA	No	NA	NA	NA	NA	NA	NA	NA
177	No	No	NA	No	NA	NA	NA	NA	NA	NA	NA
213	No	No	NA	No	NA	NA	NA	NA	NA	NA	NA
311	No	No	NA	No	NA	NA	NA	NA	NA	NA	NA
257	No	No	NA	No	NA	NA	NA	NA	NA	NA	NA
263	No	No	NA	No	NA	NA	NA	NA	NA	NA	NA
**49**	**Yes**	No	NA	No	NA	NA	NA	NA	NA	NA	NA
**42**	**Yes**	No	NA	No	NA	NA	NA	NA	NA	NA	NA
23	No	No	NA	No	NA	NA	NA	NA	NA	NA	NA
39	No	No	NA	No	NA	NA	NA	NA	NA	NA	NA
9	No	No	NA	No	NA	NA	NA	NA	NA	NA	NA

Table S1. * IRC histological assessment was scored as “insufficient evidence” due to a lack of adequate material for $IgG4^+$ staining. Abbreviations: CCA, cholangiocarcinoma; DDx, differential diagnosis; e.c.i., e causa ignota; HPB, hepatopancreatobiliary; HPF, high power field; IRC, IgG4-related cholangitis; NA, not applicable; PSC, primary sclerosing cholangitis; PPPD, pylorus-preserving pancreatoduodenectomy.

Table S2. Human H69 cholangiocyte culture medium composition for 500 ml volume

Material / Compound	Cat. No.	Supplier	Volume / Concentration
DMEM	31600-083	Gibco	375 mL
Ham's F-12 Nutrient Mix	N6760	Sigma	125 mL
Sodium Bicarbonate	144-55-8	Merck	1.794 g/L
HEPES-NaOH, pH 7.4	H3375	Sigma	20 mM
L-glutamine	BE-17-605E	Lonza	3.25 mM
Penicillin / Streptomycin	15140-122	Gibco	37.5 U/ml / 37.5 µg/ml
Fetal Bovine Serum	S00EW10003 Batch: BDC-S00EW1	Bodinco	10% (v/v)
Adenine	A2786	Sigma	180 µM
Insulin	I1882	Sigma	865 nM
Transferrin	T8158	Sigma	62.5 nM
Triiodothyronine (T3)	T3697	Sigma	2 nM
Hydrocortisone	H0888	Sigma	1.1 µM
Epinephrine	E4250	Sigma	5.5 µM
Epidermal Growth Factor	E9644	Sigma	1.67 nM

Table S3. Formulation of homemade HBSS for intracellular pH measurements by BCECF AM under 5% CO_2

Component	Final concentration (mM)	Osmolarity (mosm/L)
$CaCl_2 * 2H_2O$	1.27	3.80
KCl	4.56	9.12
$NaH_2PO_4 * 2H_2O$	0.45	0.90
$MgSO_4 * 7H_2O$	0.41	0.81
$MgCl_2 * 6H_2O$	0.39	1.18
NaCl	107.80	215.61
$NaHCO_3^-$	21.43	42.85
$Na_2HPO_4 * 2H_2O$	0.34	1.01
HEPES-NaOH, pH 7.4	20	30
Glucose	5.55	5.55

Supplementary references

Author names in bold designate shared co-first authorship.

1. **Roos E, Hubers LM**, Coelen RJS, Doorenspleet ME, de Vries N et al. IgG4-Associated Cholangitis in Patients Resected for Presumed Perihilar Cholangiocarcinoma: a 30-Year Tertiary Care Experience. Am J Gastroenterol. 2018; 113: 765-772. doi: 10.1038/s41395-018-0036-5.
2. Deshpande V, Zen Y, Chan JKC, Yi EE, Sato Y et al. Consensus statement on the pathology of IgG4-related disease. Mod Pathol. 2012; 25: 1181-92. doi: 10.1038/modpathol.2012.72.
3. Xu L, Hui AY, Albanis E, Arthur MJ, O'Byrne SM et al. Human hepatic stellate cell lines, LX-1 and LX-2: new tools for analysis of hepatic fibrosis. Gut. 2005; 54: 142-51. doi: 10.1136/gut.2004.042127.
4. Smith-Cortinez N, Fagundes RR, Gomez V, Kong D, de Waart DR et al. Collagen release by human hepatic stellate cells requires vitamin C and is efficiently blocked by hydroxylase inhibition. FASEB J. 2021; 35: e21219. doi: 10.1096/fj.202001564RR.
5. Verstegen MMA, Roos FJM, Burka K, Gehart H, Jager M, de Wolf M et al. Human extrahepatic and intrahepatic cholangiocyte organoids show region-specific differentiation potential and model cystic fibrosis-related bile duct disease. Sci Rep. 2020; 10: 21900. doi: 10.1038/s41598-020-79082-8.
6. Bolger AM, Lohse M, Usadel B. Trimmomatic: a flexible trimmer for Illumina sequence data. Bioinformatics. 2014; 30: 2114-20. doi: 10.1093/bioinformatics/btu170.
7. Kim D, Langmead B, Salzberg SL. HISAT: a fast spliced aligner with low memory requirements. Nat Methods Nat Methods. 2015; 12: 357-60. doi: 10.1038/nmeth.3317.
8. Sayols S, Scherzinger D, Klein H. dupRadar: a Bioconductor package for the assessment of PCR artifacts in RNA-Seq data. BMC Bioinformatics. 2016; 17: 428. doi: 10.1186/s12859-016-1276-2.
9. Anders S, Pyl PT, Huber W. HTSeq-A Python framework to work with high-throughput sequencing data. Bioinformatics. 2015; 31: 166-9. doi: 10.1093/bioinformatics/btu638.

10. Robinson MD, Oshlack A. A scaling normalization method for differential expression analysis of RNA-seq data. Genome Biol. 2010; 11: R25. doi: 10.1186/gb-2010-11-3-r25.
11. **Robinson MD, McCarthy DJ**, Smyth GK. edgeR: A Bioconductor package for differential expression analysis of digital gene expression data. Bioinformatics. 2010; 26: 139-40. doi: 10.1093/bioinformatics/btp616.
12. Ritchie ME, Phipson B, Wu D, Hu Y, Law CW, Shi W et al. Limma powers differential expression analyses for RNA-sequencing and microarray studies. Nucleic Acids Res. 2015; 43: e47. doi: 10.1093/nar/gkv007.
13. Wu D, Smyth GK. Camera: a competitive gene set test accounting for inter-gene correlation. Nucleic Acids Res. 2012; 40: e133. doi: 10.1093/nar/gks461.
14. Aizarani N, **Saviano A, Sagar**, Mailly L, Durand S, Herman JS et al. A human liver cell atlas reveals heterogeneity and epithelial progenitors. Nature. 2019; 572: 199-204. doi: 10.1038/s41586-019-1373-2.
15. Lei L, Bruneau A, El Mourabit H, Guégan J, Folseraas T, Lemoinne S et al. Portal fibroblasts with mesenchymal stem cell features form a reservoir of proliferative myofibroblasts in liver fibrosis. Hepatology. 2022; 76 :1360-1375. doi: 10.1002/hep.32456.
16. Ramakers C, Ruijter JM, Deprez RHL, Moorman AFM. Assumption-free analysis of quantitative real-time polymerase chain reaction (PCR) data. Neurosci Lett 2003;339:62–6. https://doi.org/10.1016/s0304-3940(02)01423-4
17. **Madeira F, Pearce M**, Tivey ARN, Basutkar P, Lee J, Edbali O et al. Search and sequence analysis tools services from EMBL-EBI in 2022. Nucleic Acids Res. 2022; 50: W276-W279. doi: 10.1093/nar/gkac240.
18. Jian Ye J, Coulouris G, Zaretskaya I, Cutcutache I, Rozen S, Madden TL. Primer-BLAST: a tool to design target-specific primers for polymerase chain reaction. BMC Bioinformatics. 2012; 13: 134. doi: 10.1186/1471-2105-13-134.
19. Chang JC, Go S, de Waart DR, Munoz-Garrido P, Beuers U, Paulusma, C, Oude Elferink, R. Soluble Adenylyl Cyclase Regulates Bile Salt-Induced Apoptosis in Human Cholangiocytes. Hepatology. 2016; 64: 522-34. doi: 10.1002/hep.28550.
20. Brown AM, A step-by-step guide to non-linear regression analysis of experimental data using a Microsoft Excel spreadsheet. Comput Methods Programs Biomed. 2001; 65:191-200. doi: 10.1016/s0169-2607(00)00124-3.

21. Kemmer G, Keller S. Nonlinear least-squares data fitting in Excel spreadsheets. Nat Protoc. 2010; 5: 267-81. doi: 10.1038/nprot.2009.182.
